# Supplementary figures and images for: Four-Types of IIT-Induced Group Integrity of Plecoglossus altivelis
Source: Entropy (Basel). 2020 Jun 30;22(7):726. doi: 10.3390/e22070726 (PMC7517268; doi:10.3390/e22070726)

$$\xi_{TR}=0 \text{ (rad/step)}$$

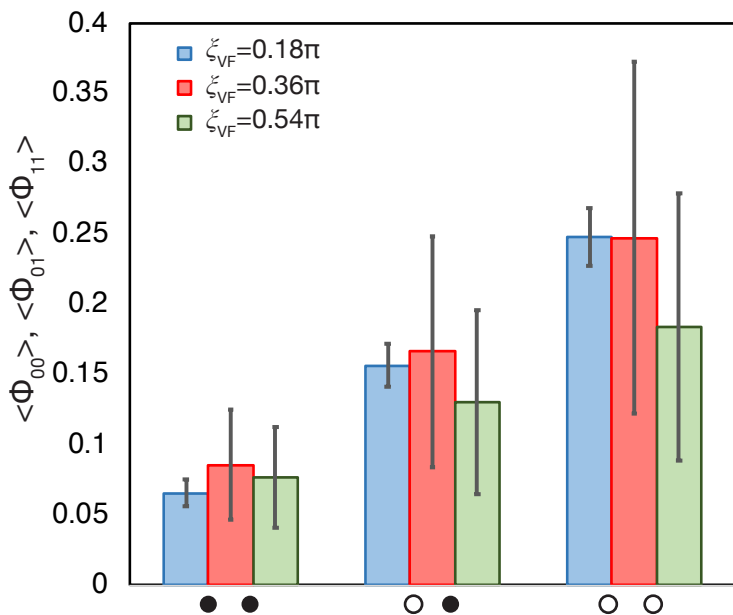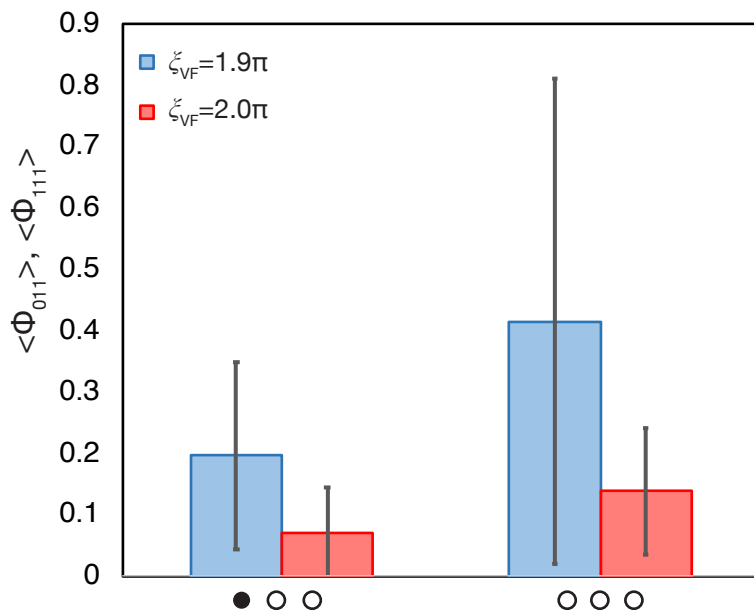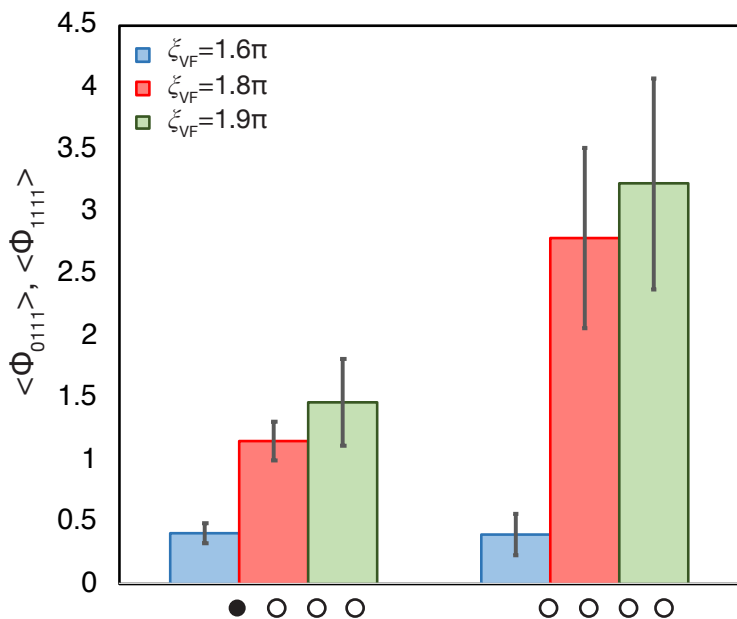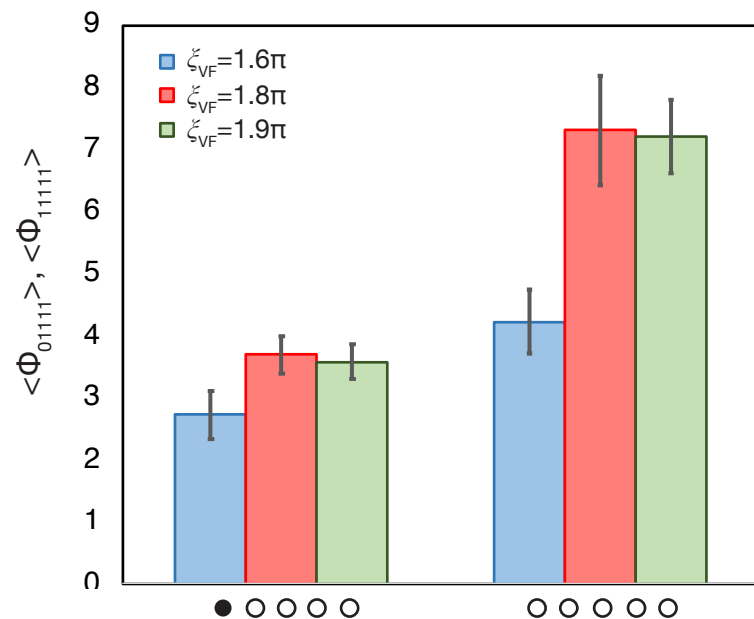

$$\xi_{TR}=0.005 \text{ (rad/step)}$$

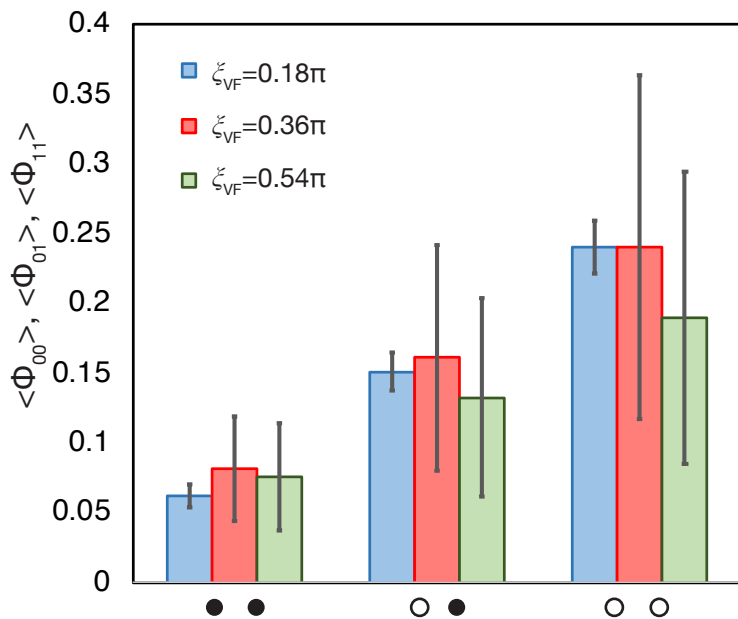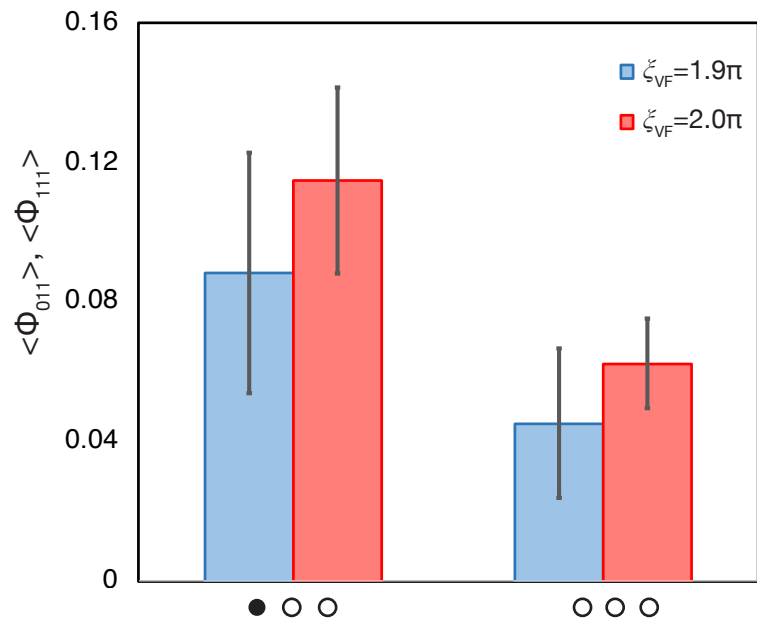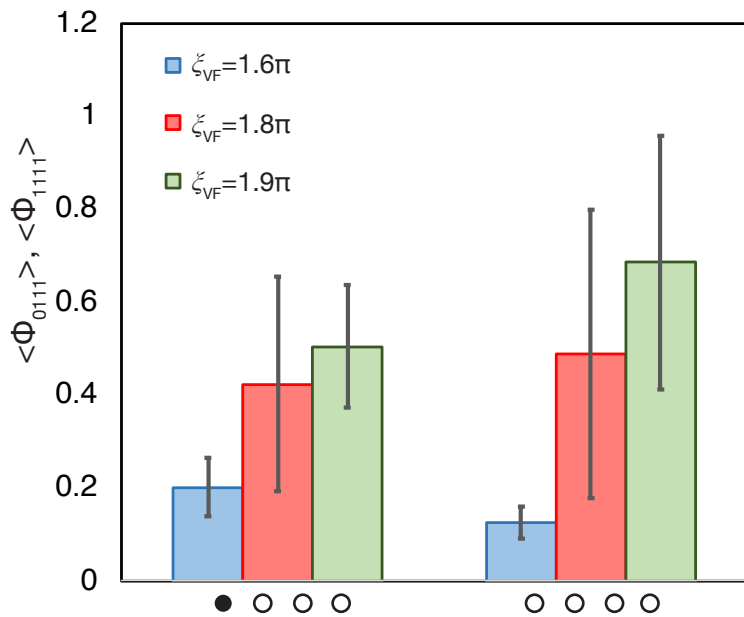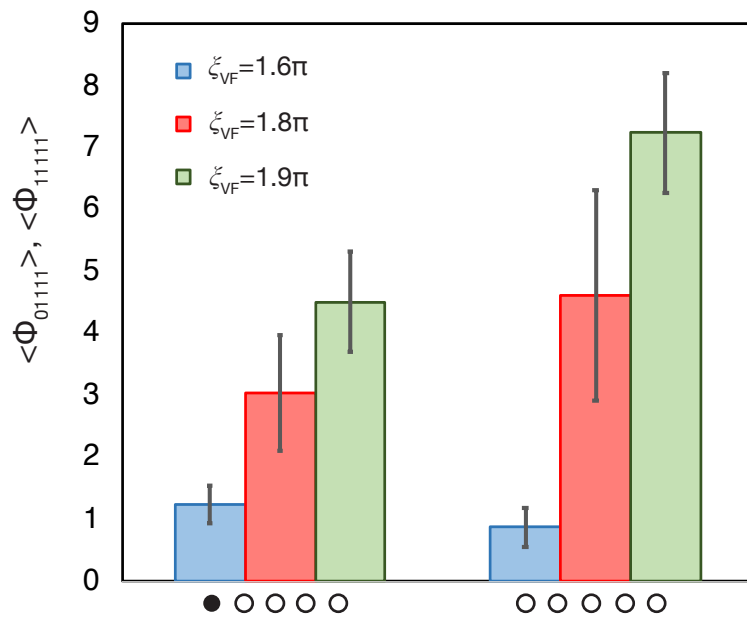

Supplement: Supplementary file 1 [file entropy-22-00726-s001.zip › Supporting Information/Figure_S9.pdf]

time : 700

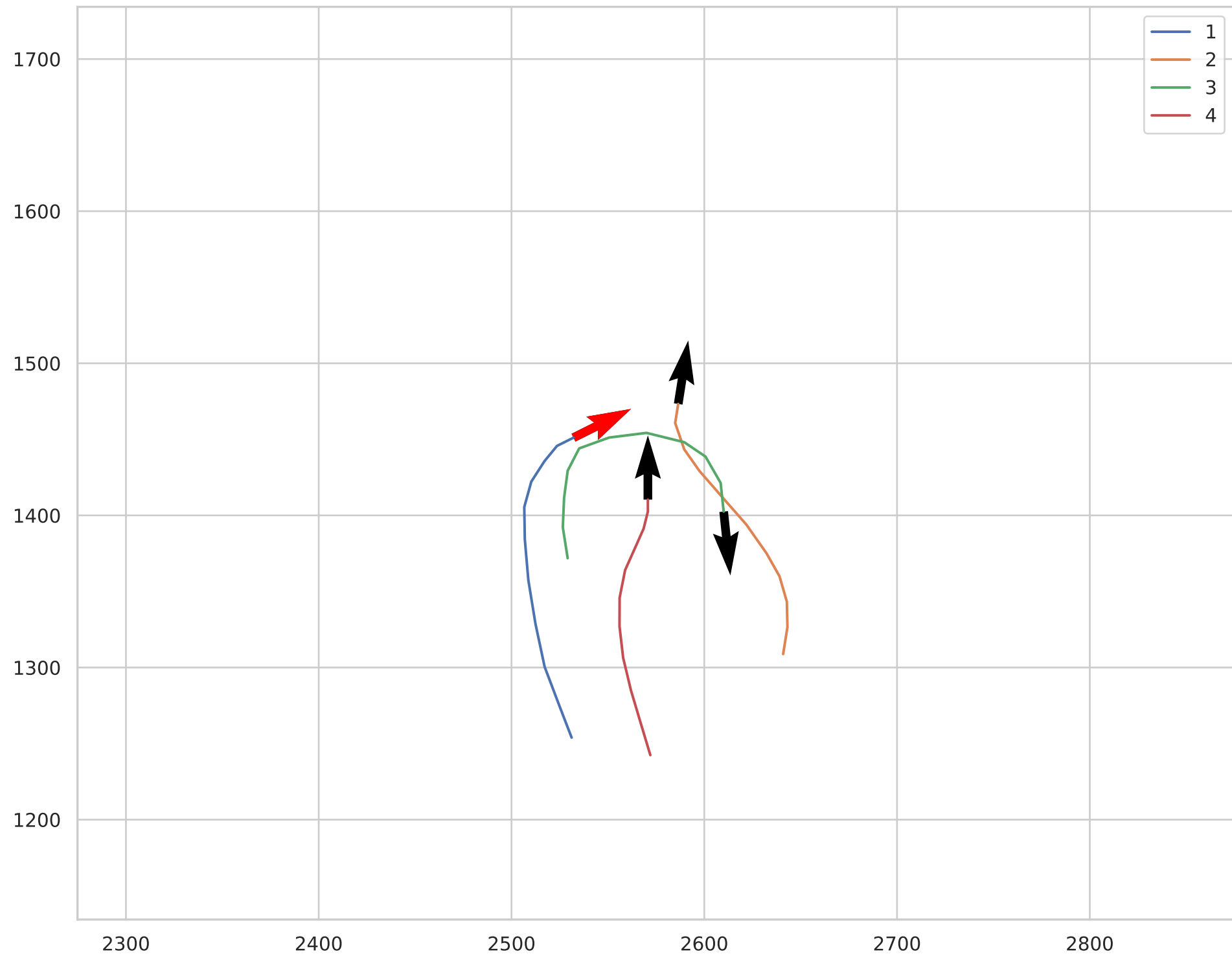

time : 1554

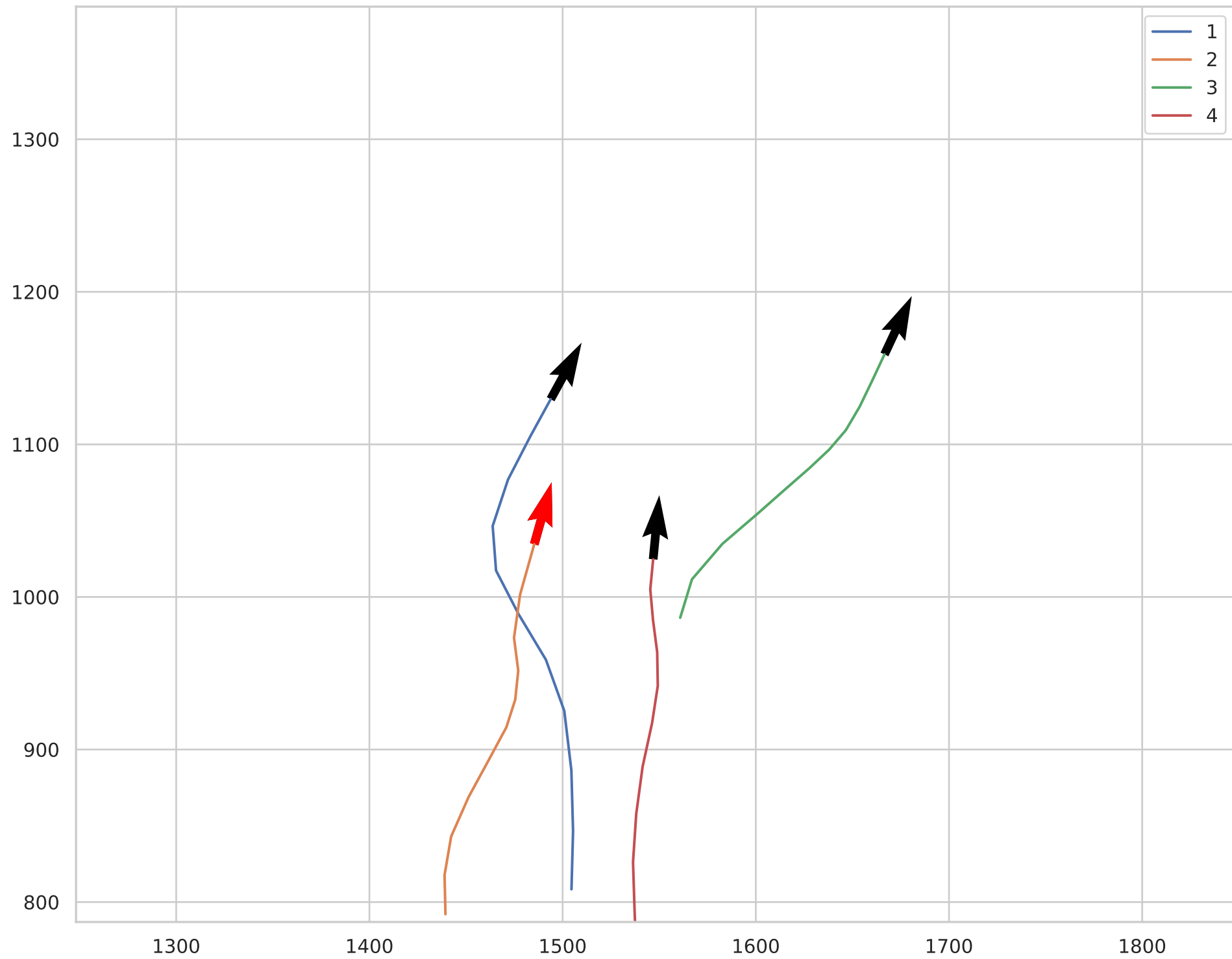

time : 3202

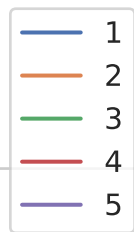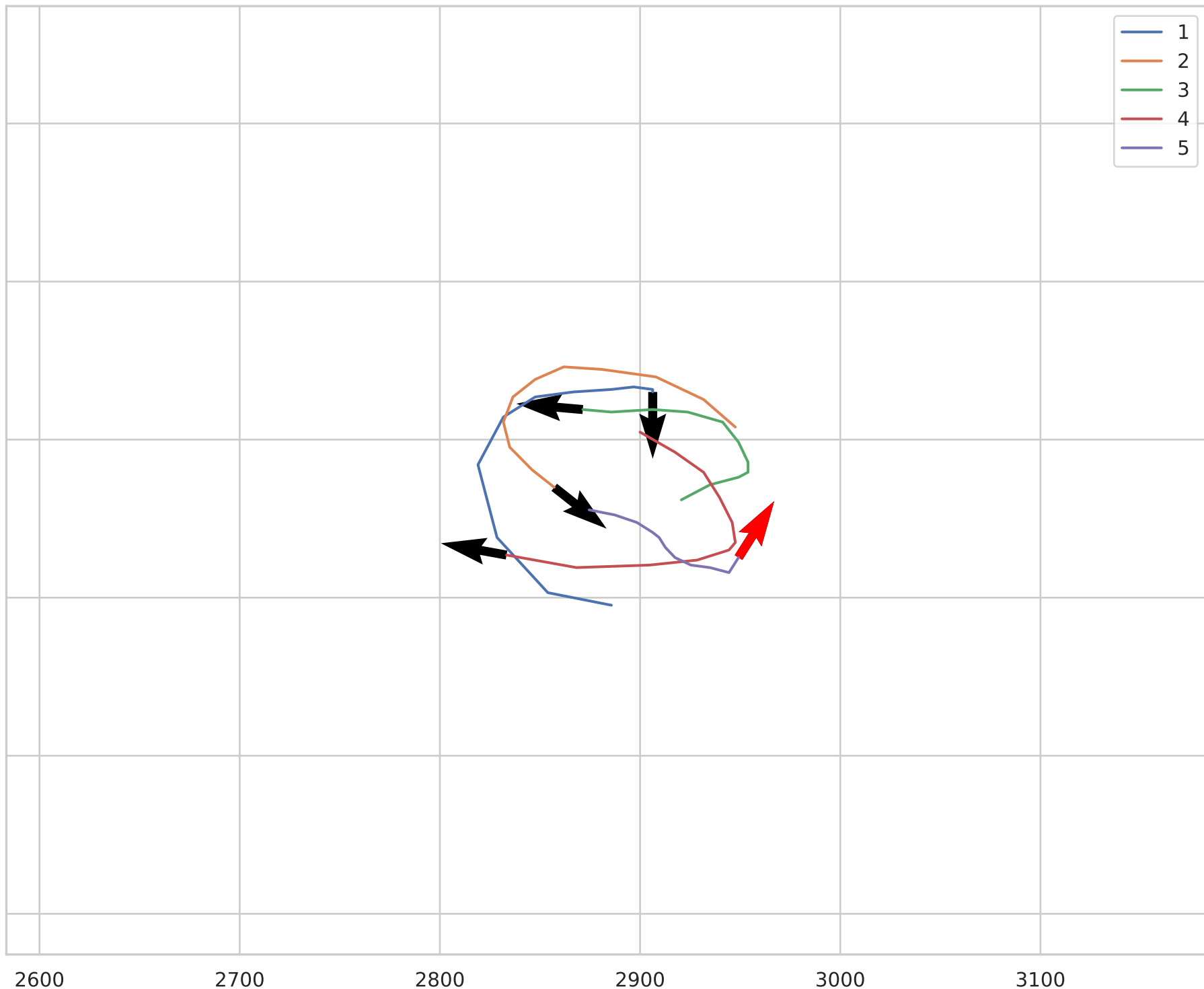

Supplement: Supplementary file 1 [file entropy-22-00726-s001.zip › Supporting Information/Figure_S8.pdf]

dt = 5/120 sec

N=2

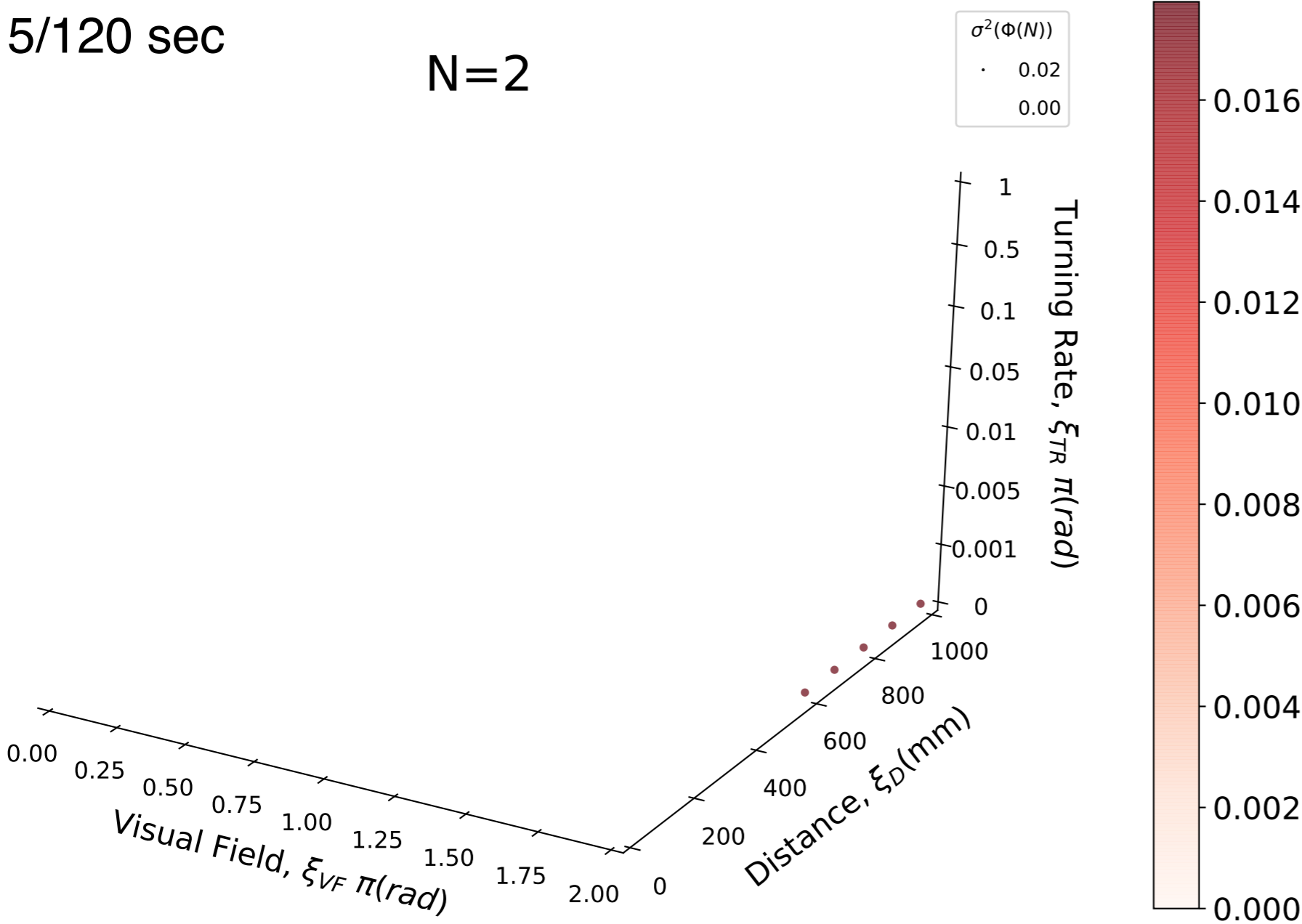

N=3

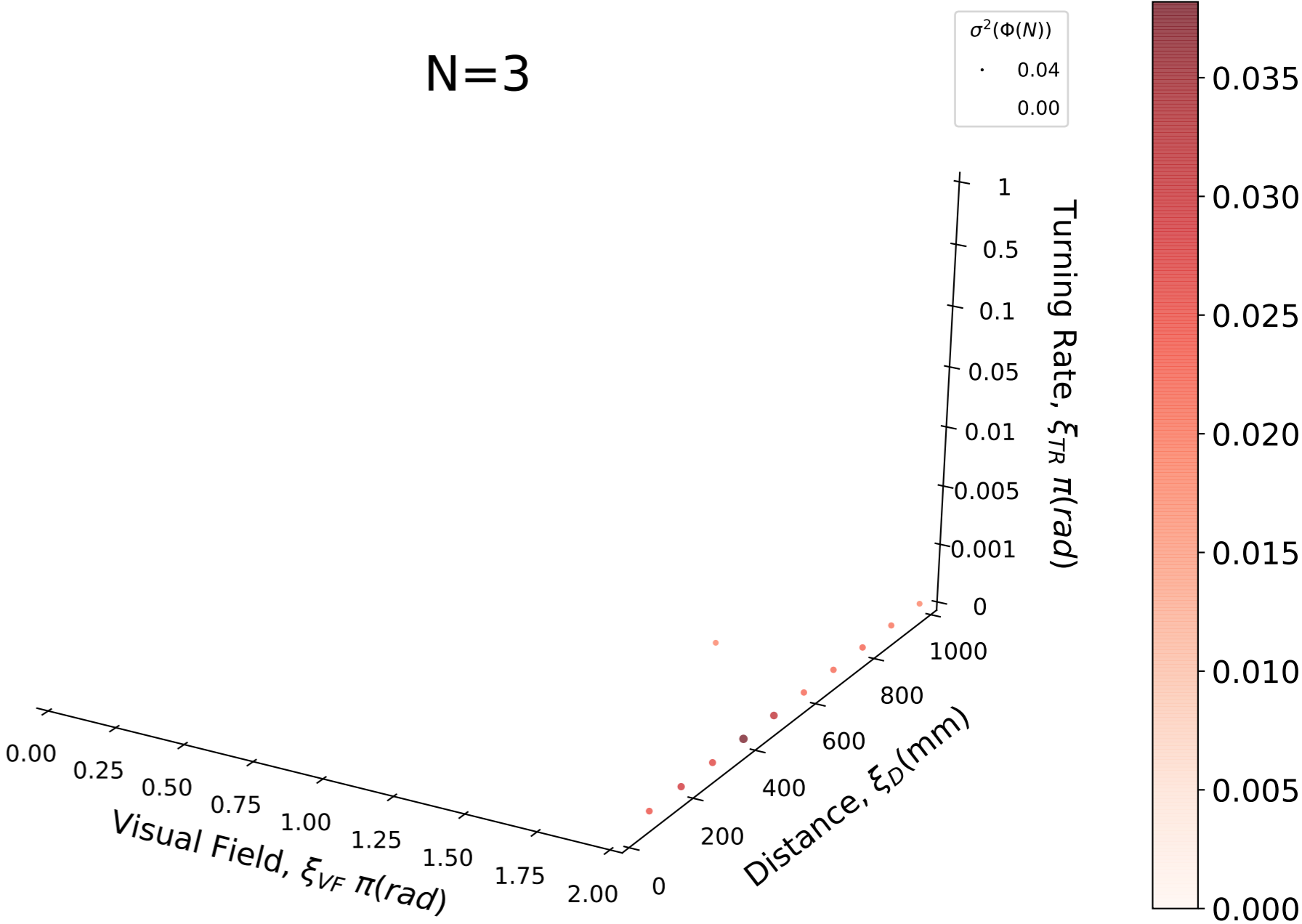

N=4

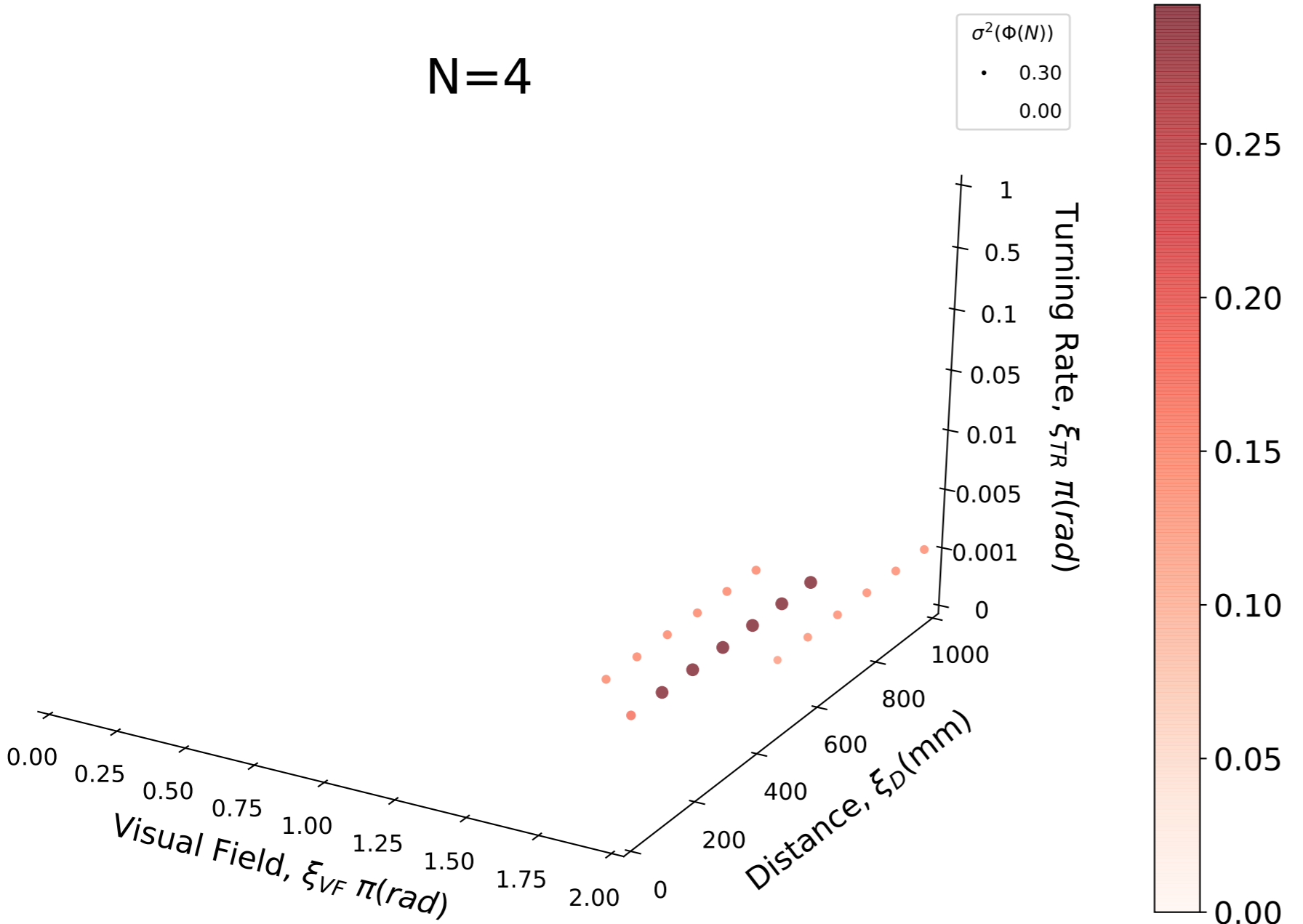

N=5

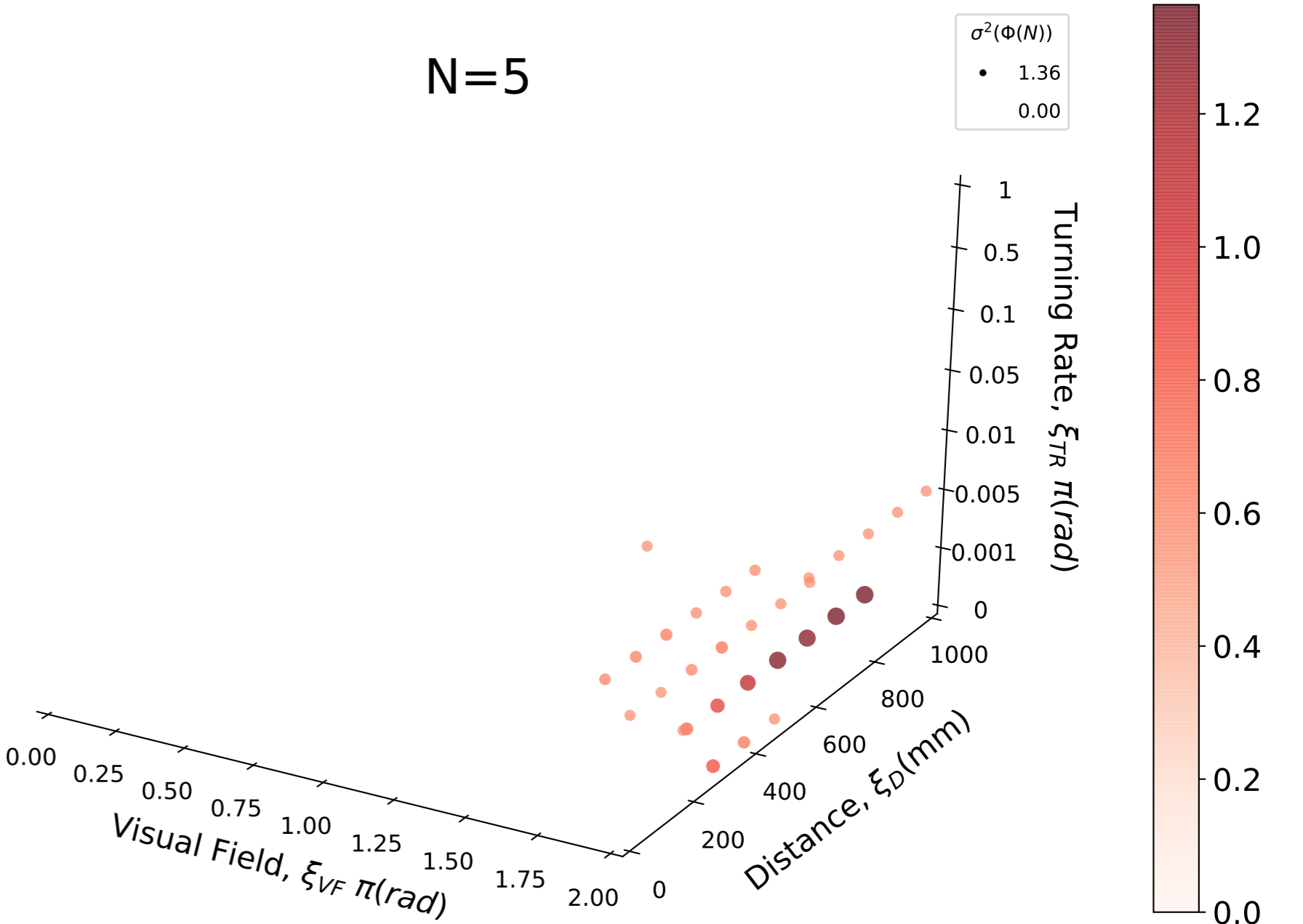

dt = 10/120 sec

N=2

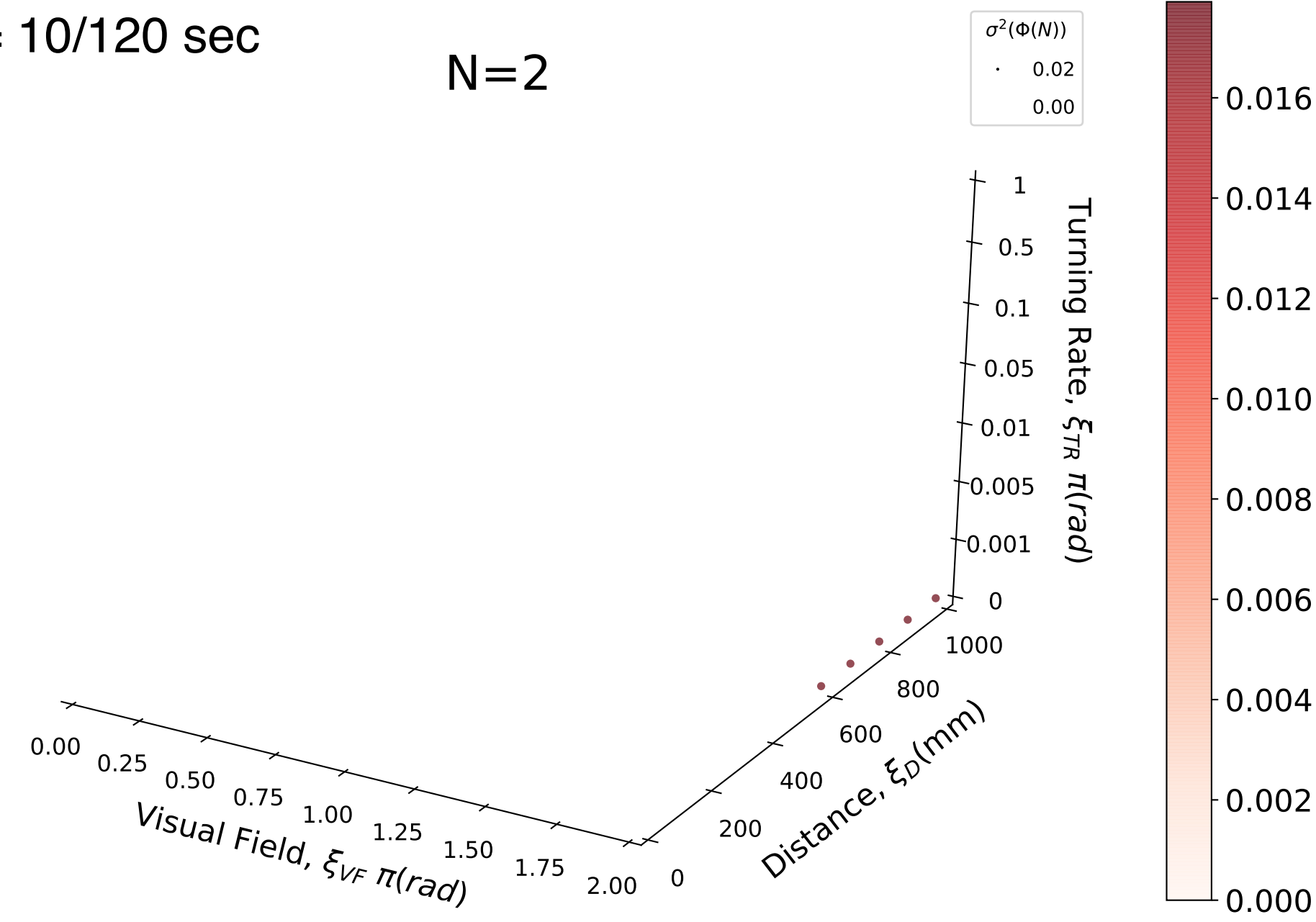

N=3

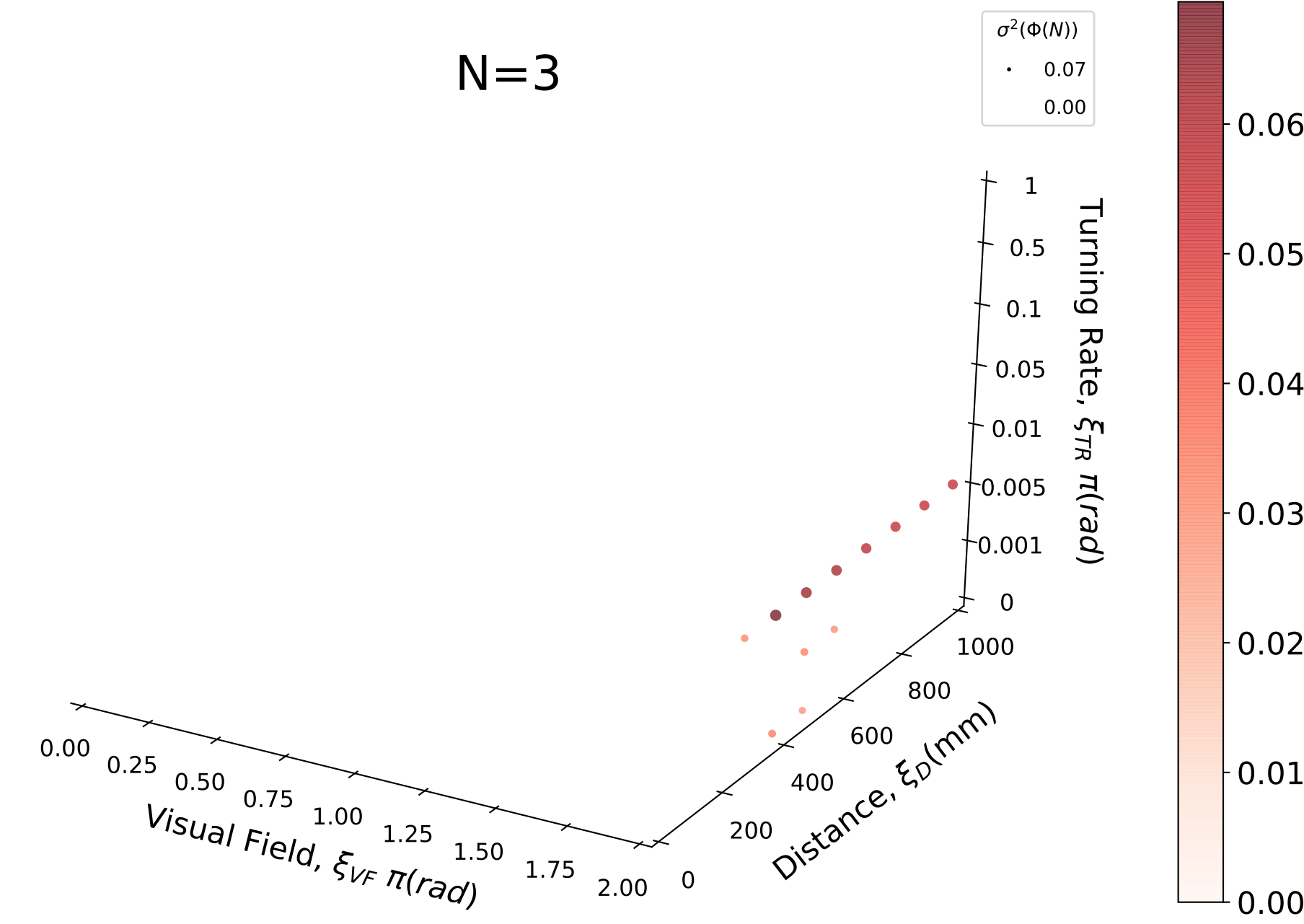

N=4

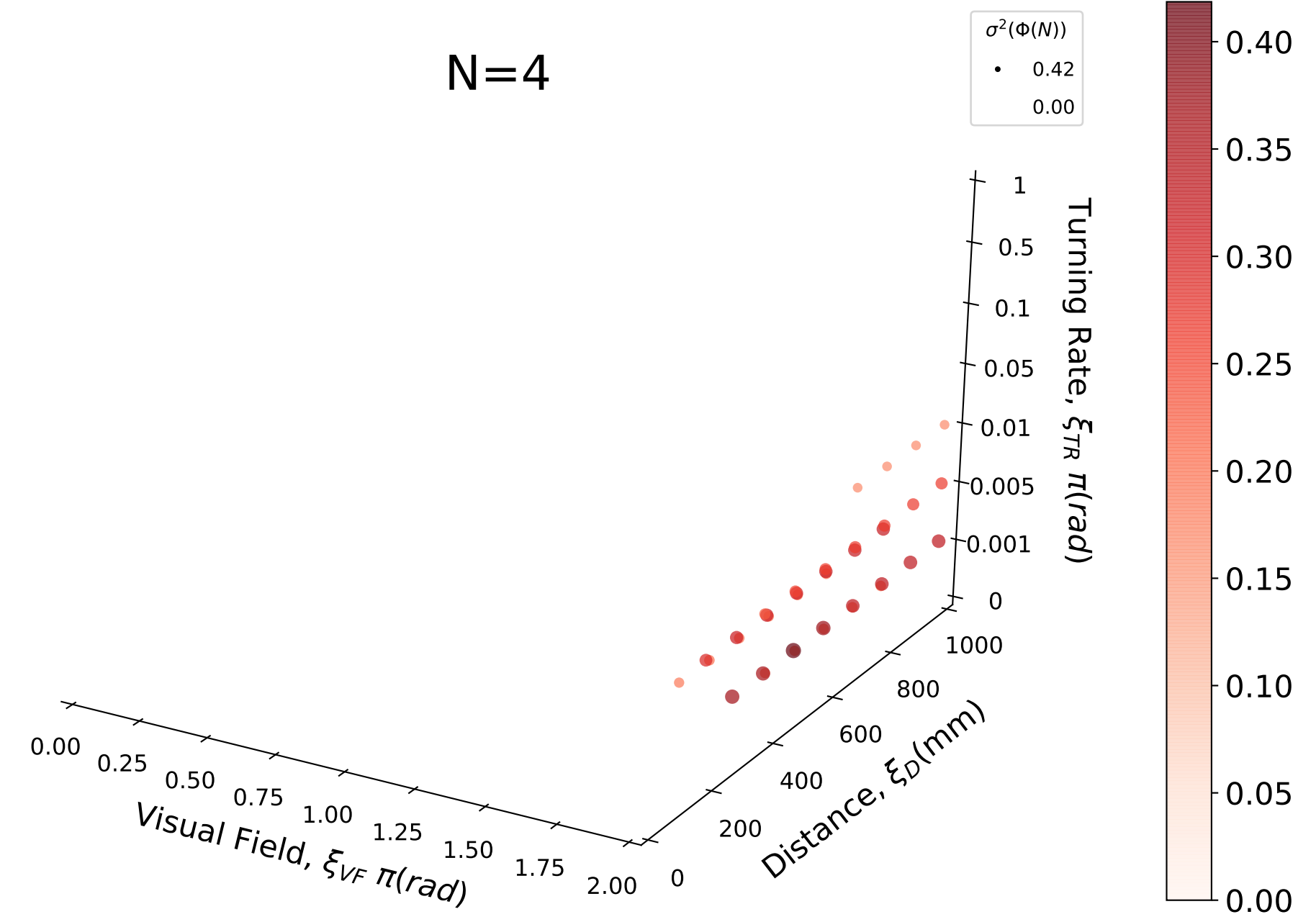

N=5

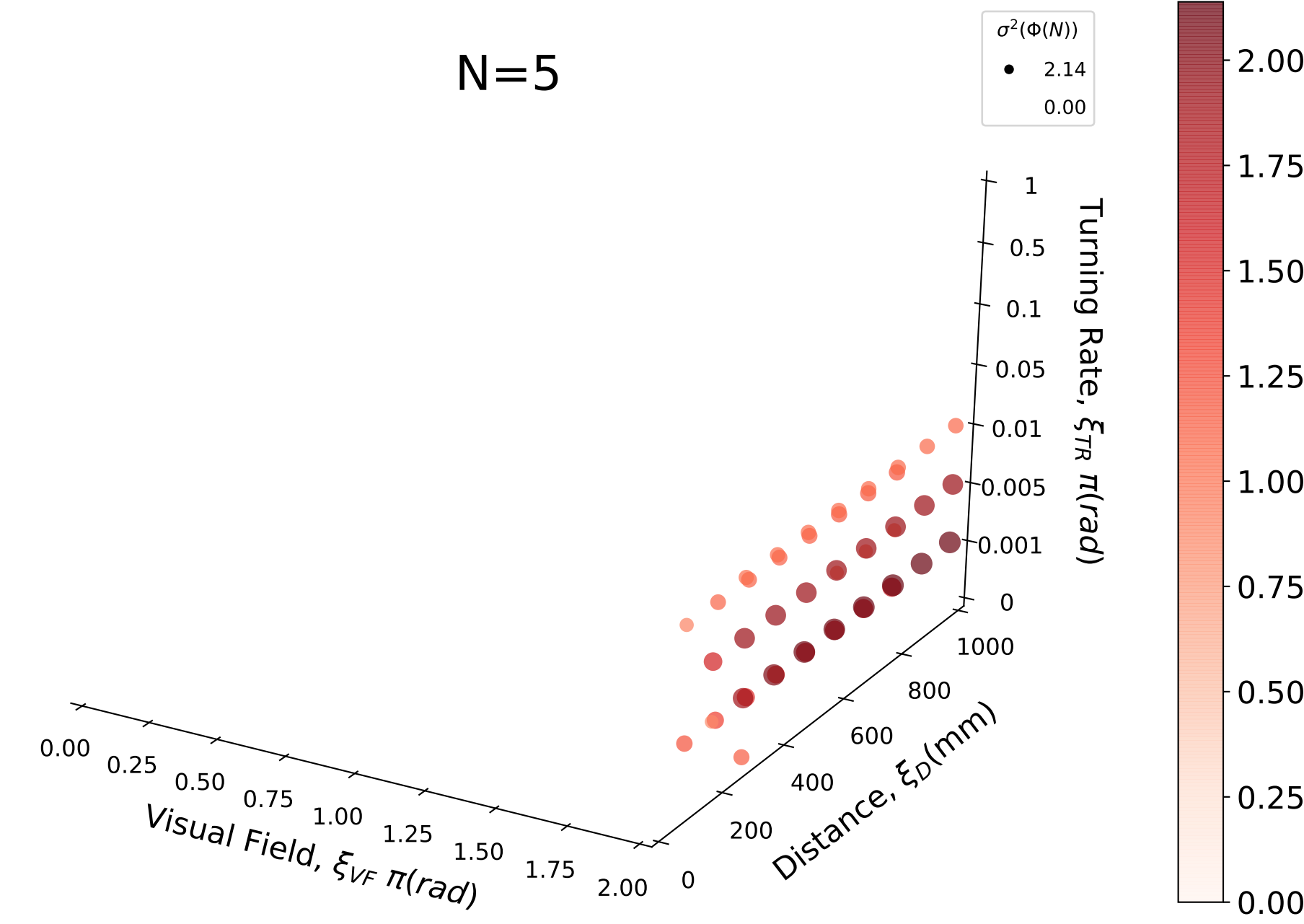

dt = 20/120 sec

N=2

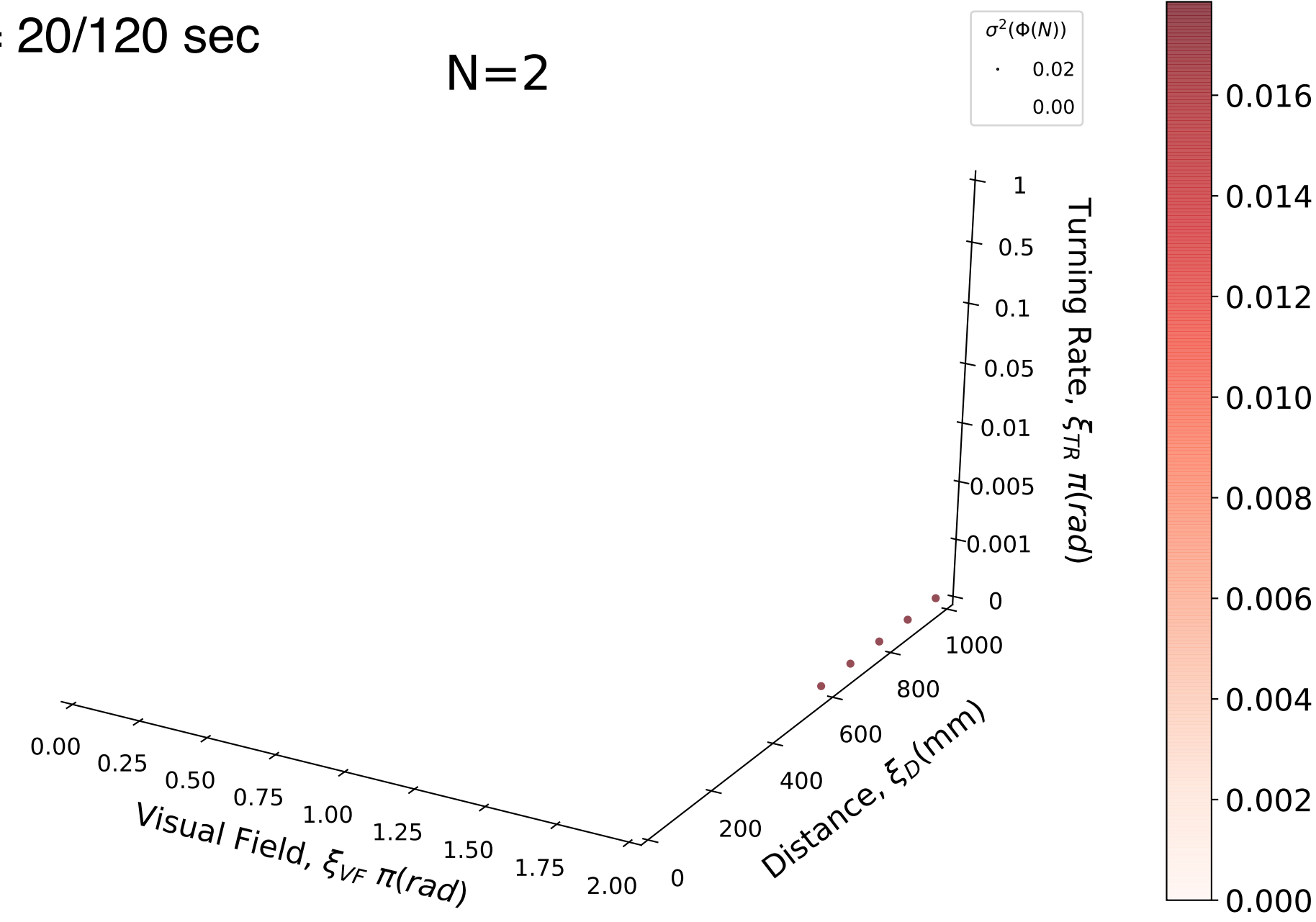

N=3

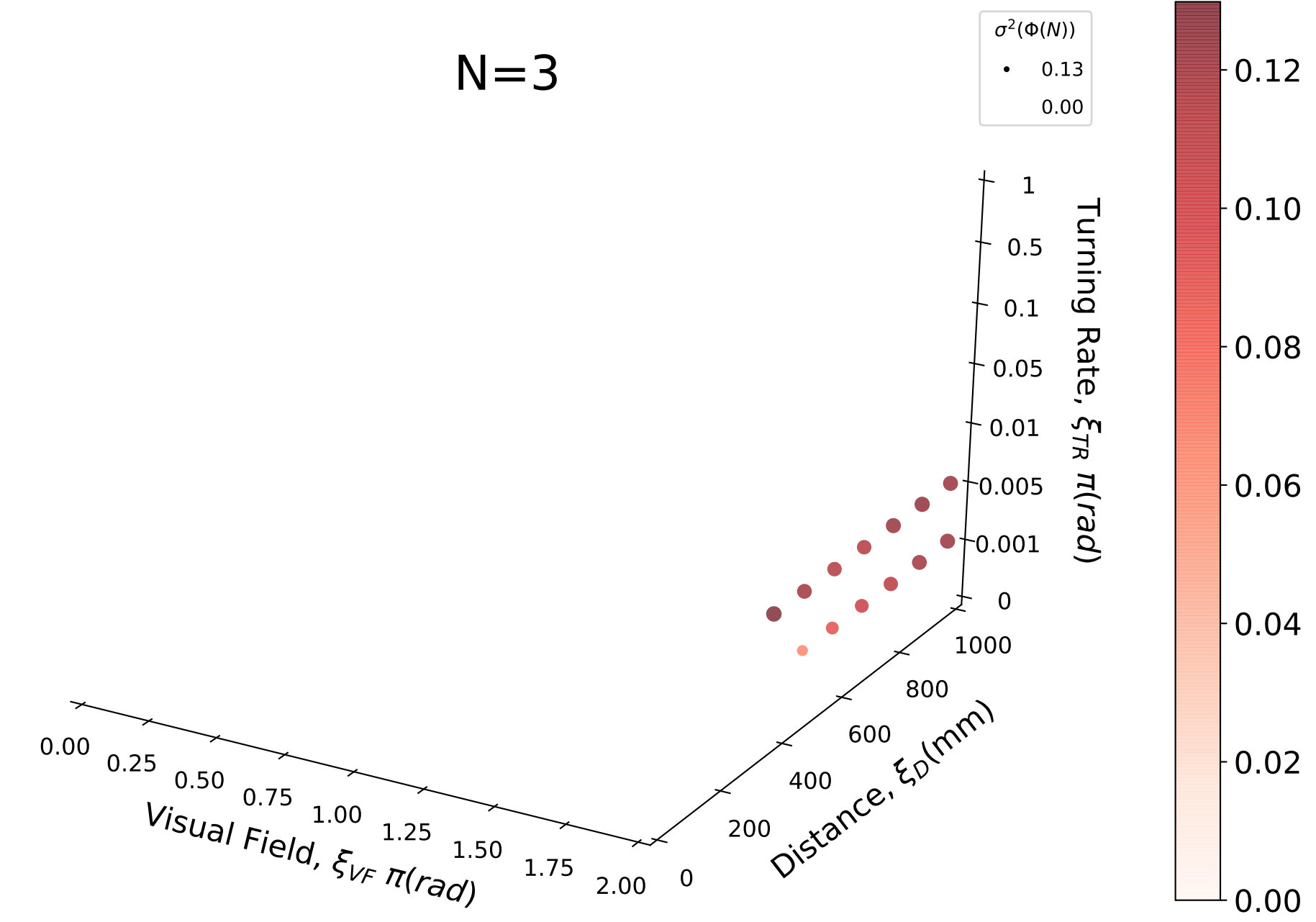

N=4

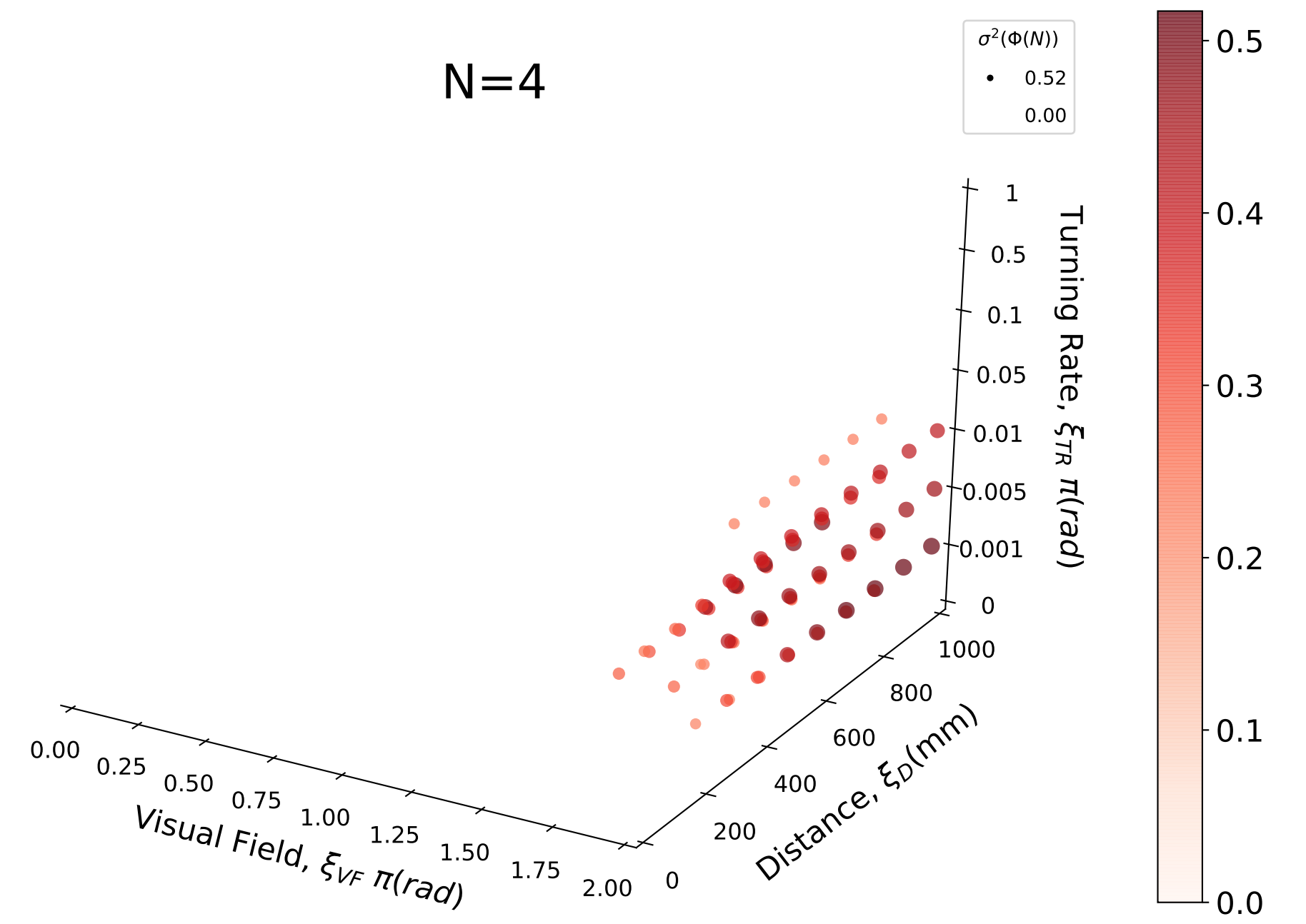

N=5

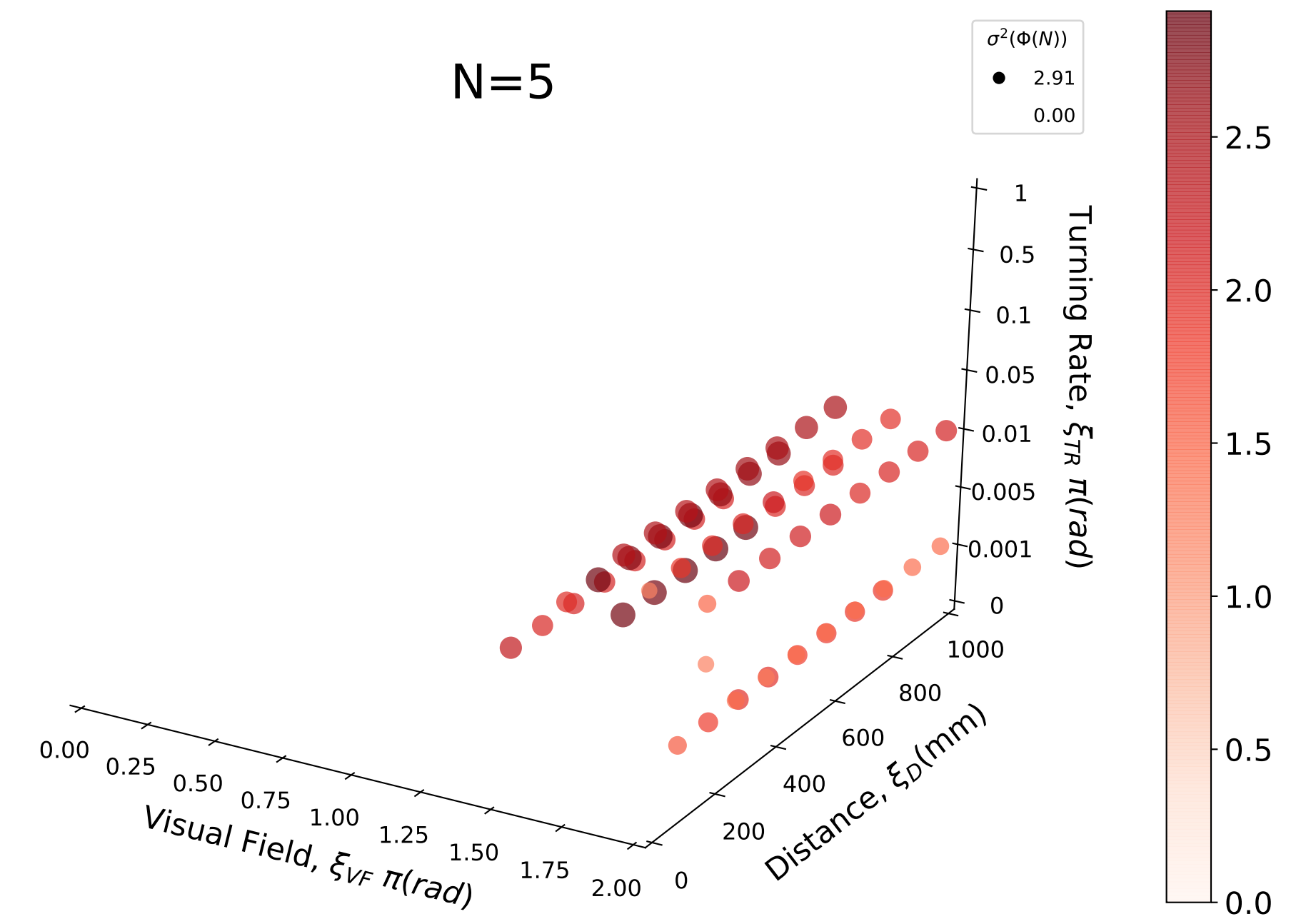

dt = 40/120 sec

N=2

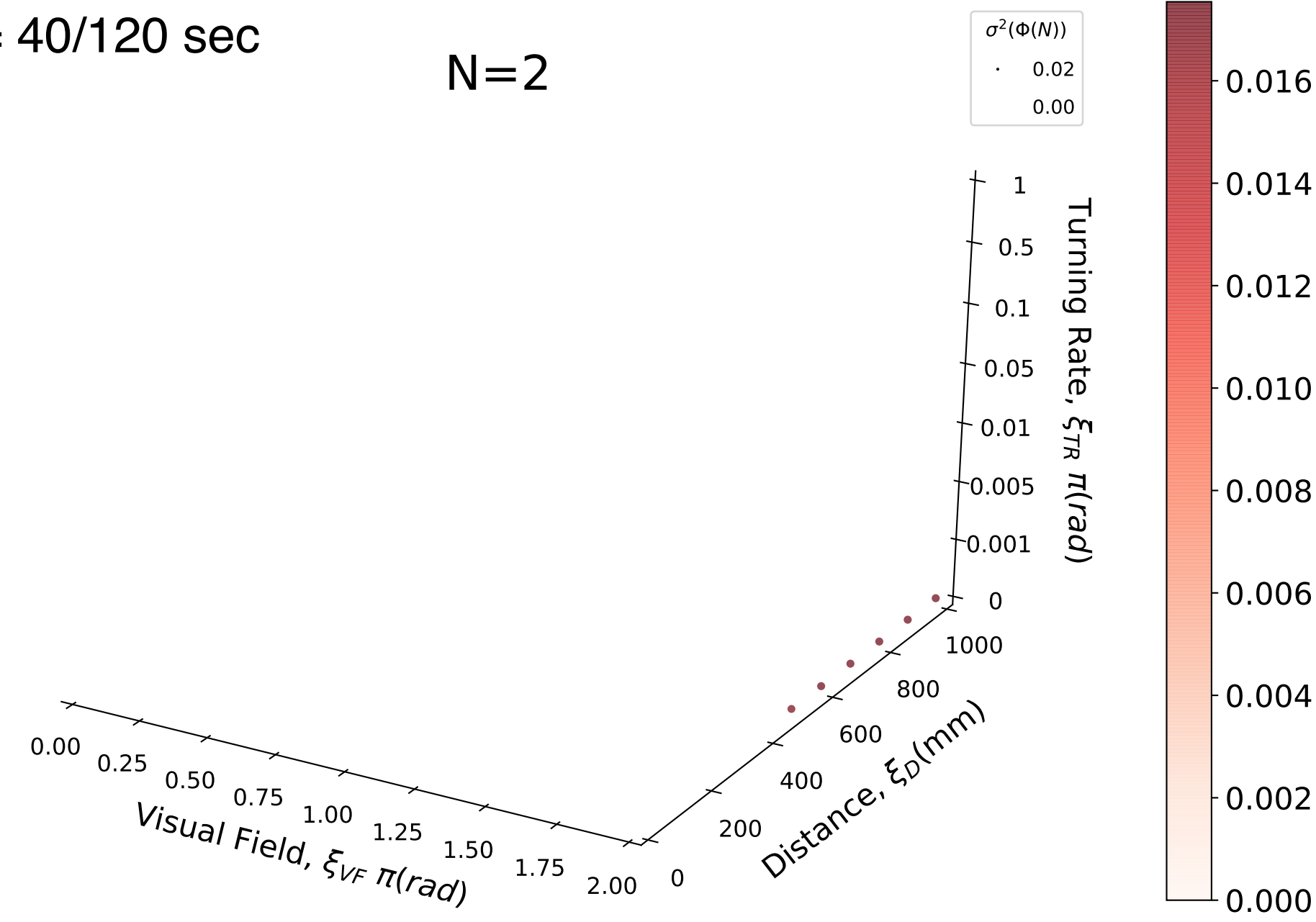

N=3

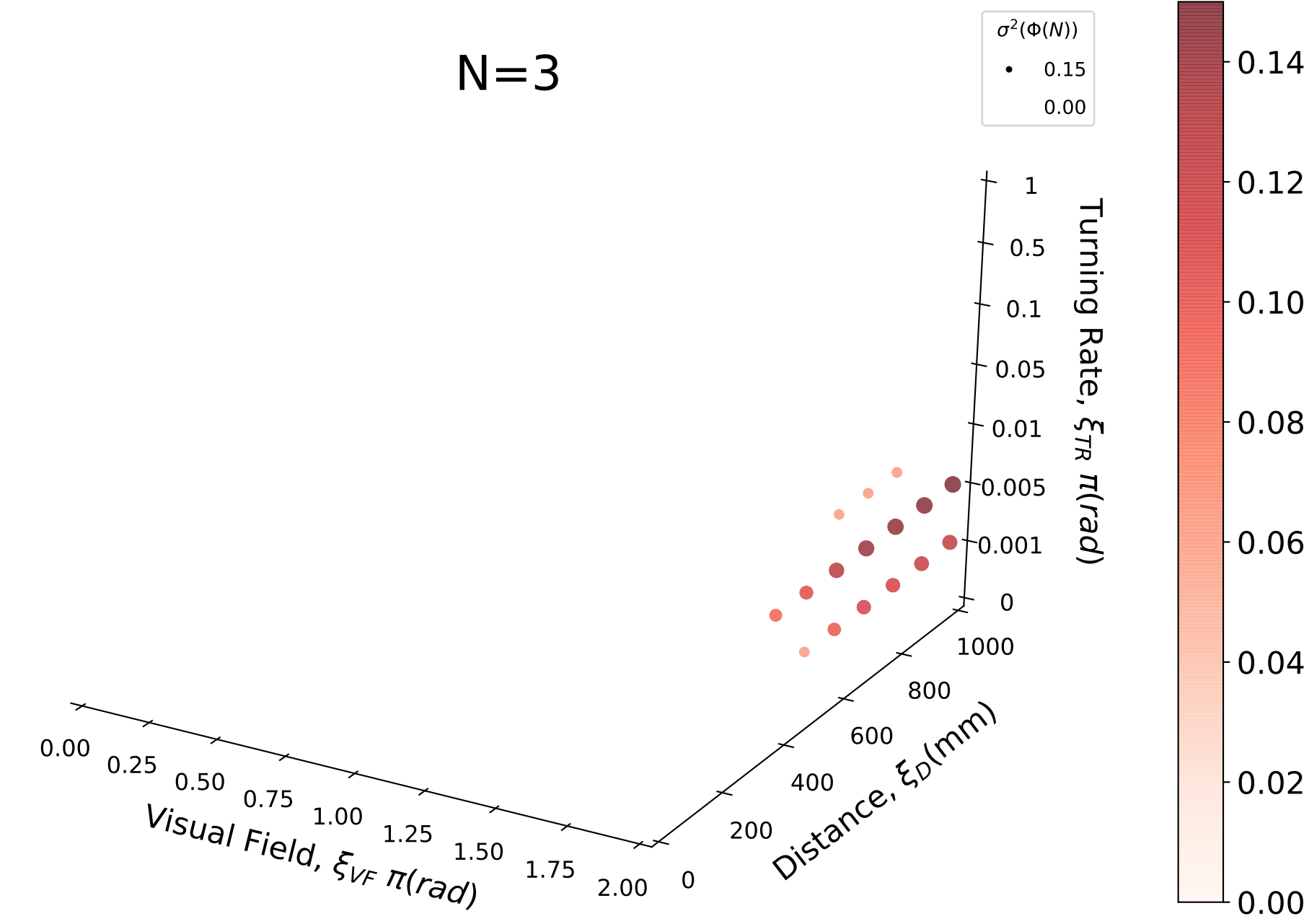

N=4

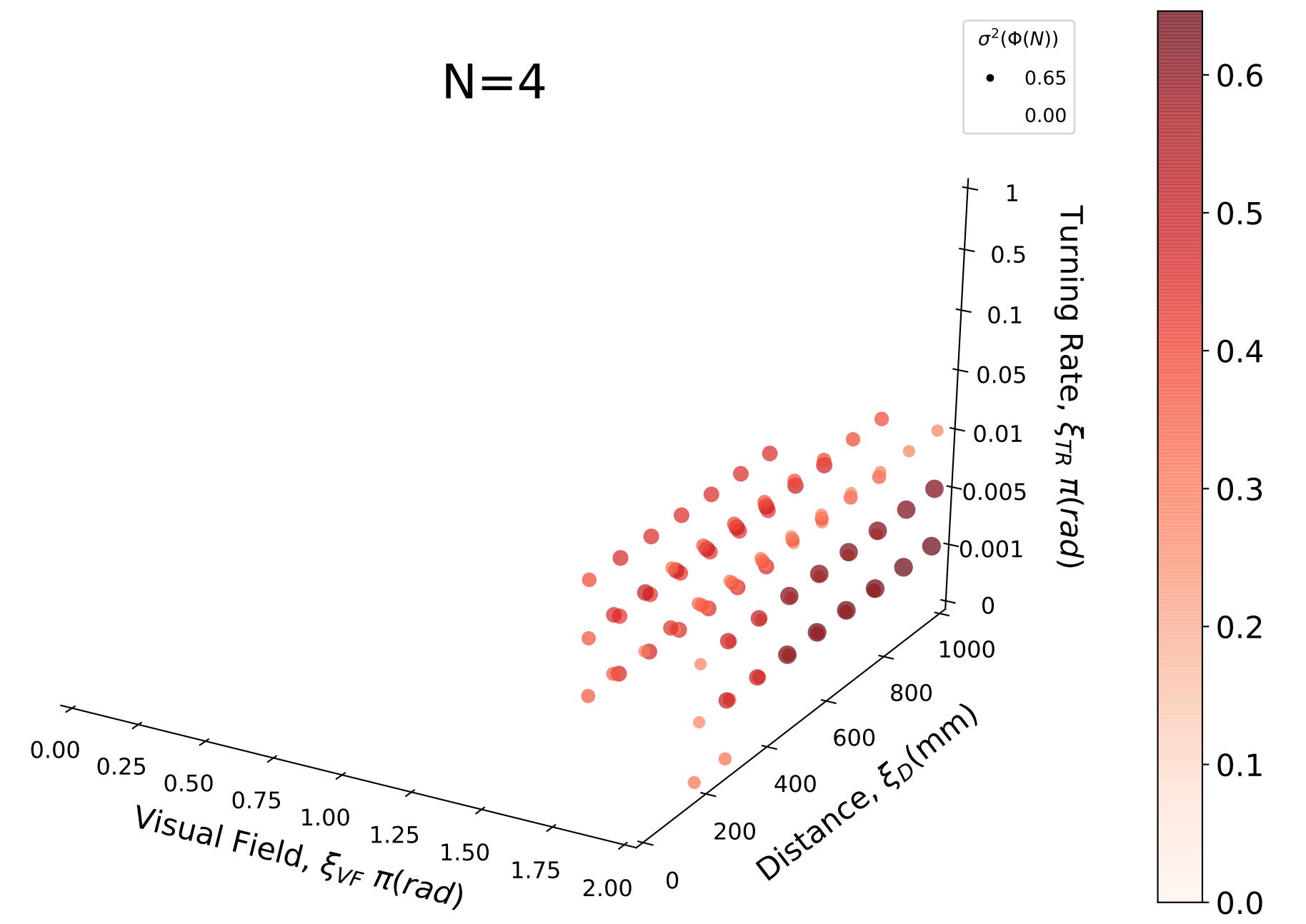

N=5

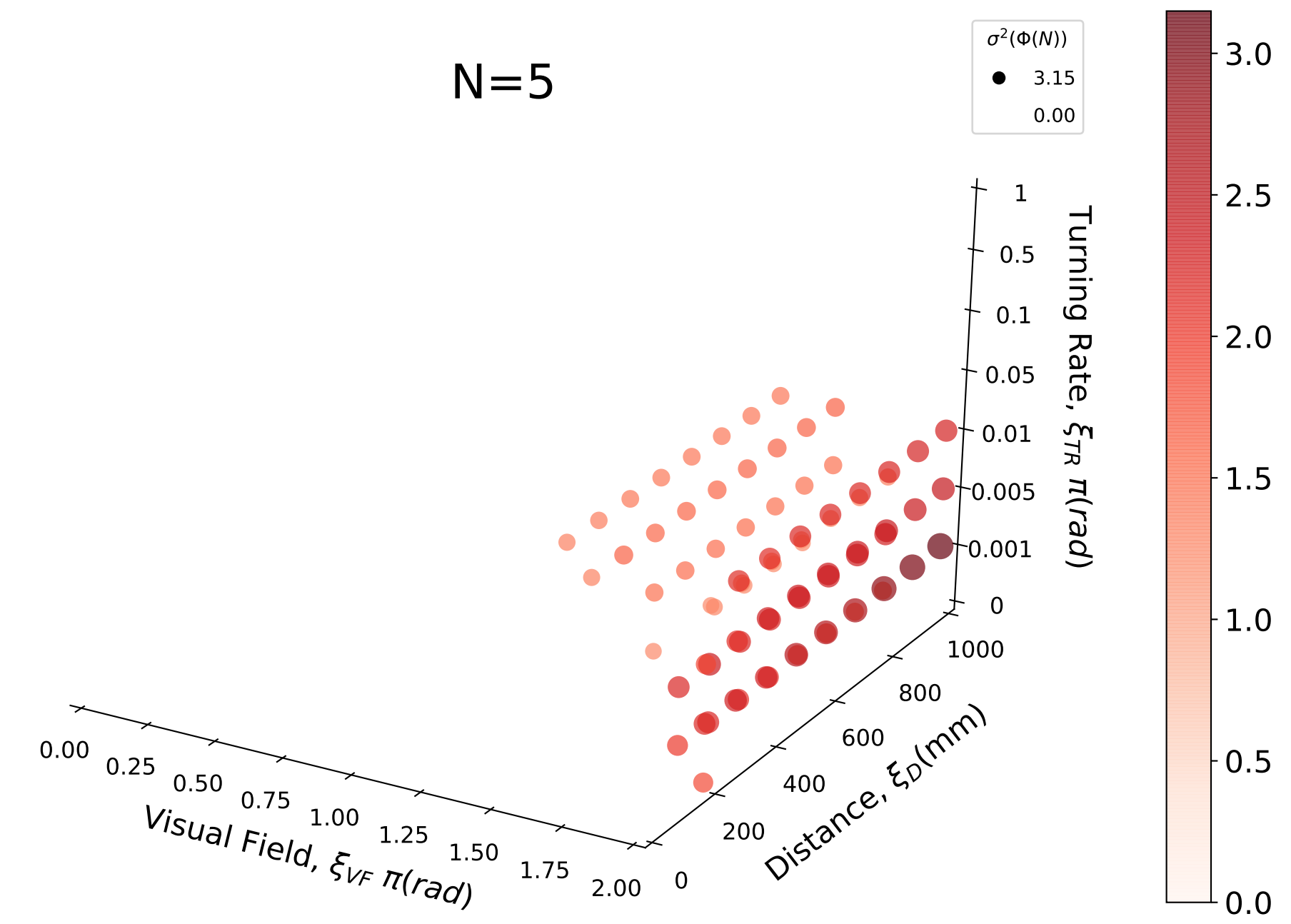

dt = 80/120 sec

N=2

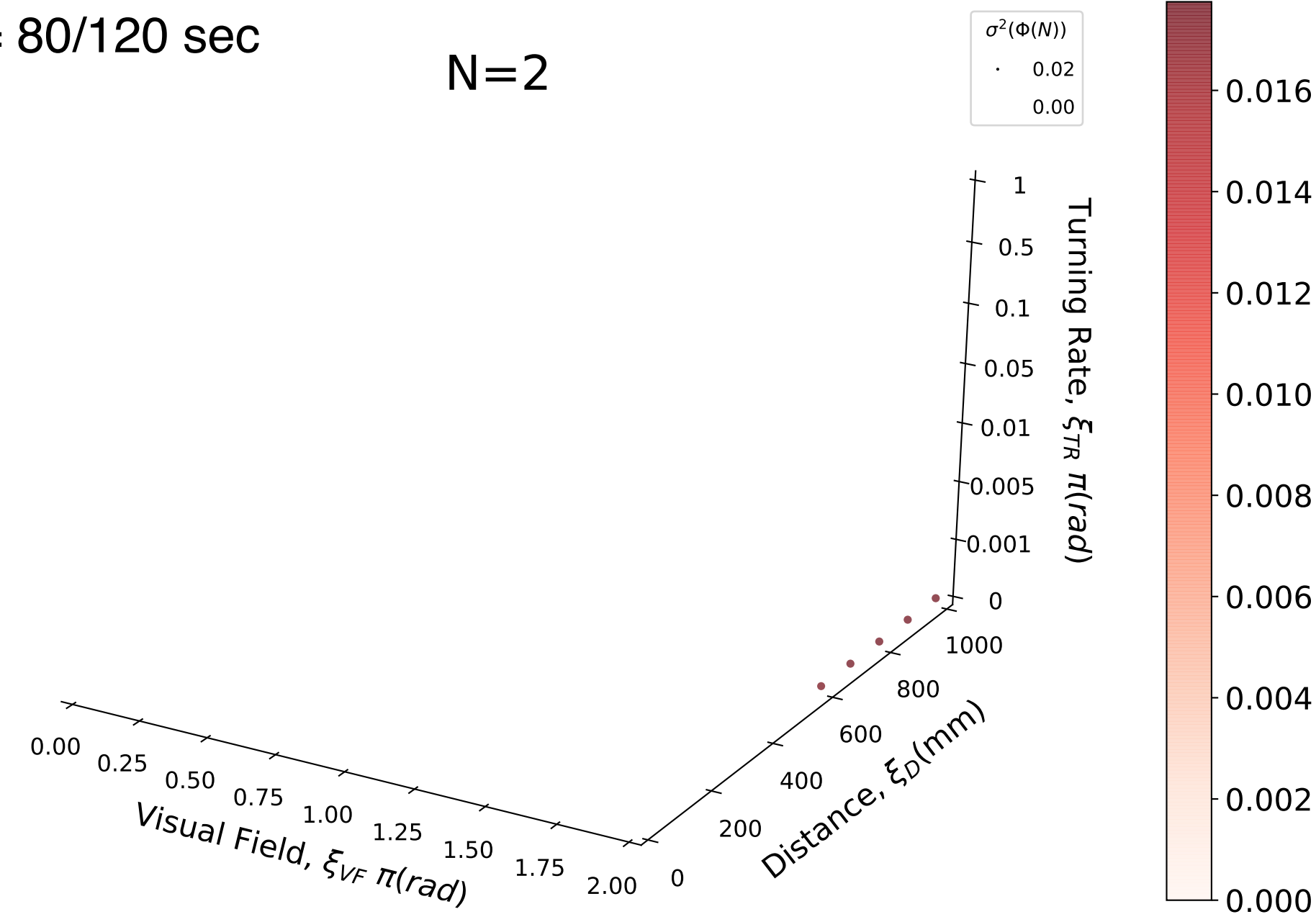

N=3

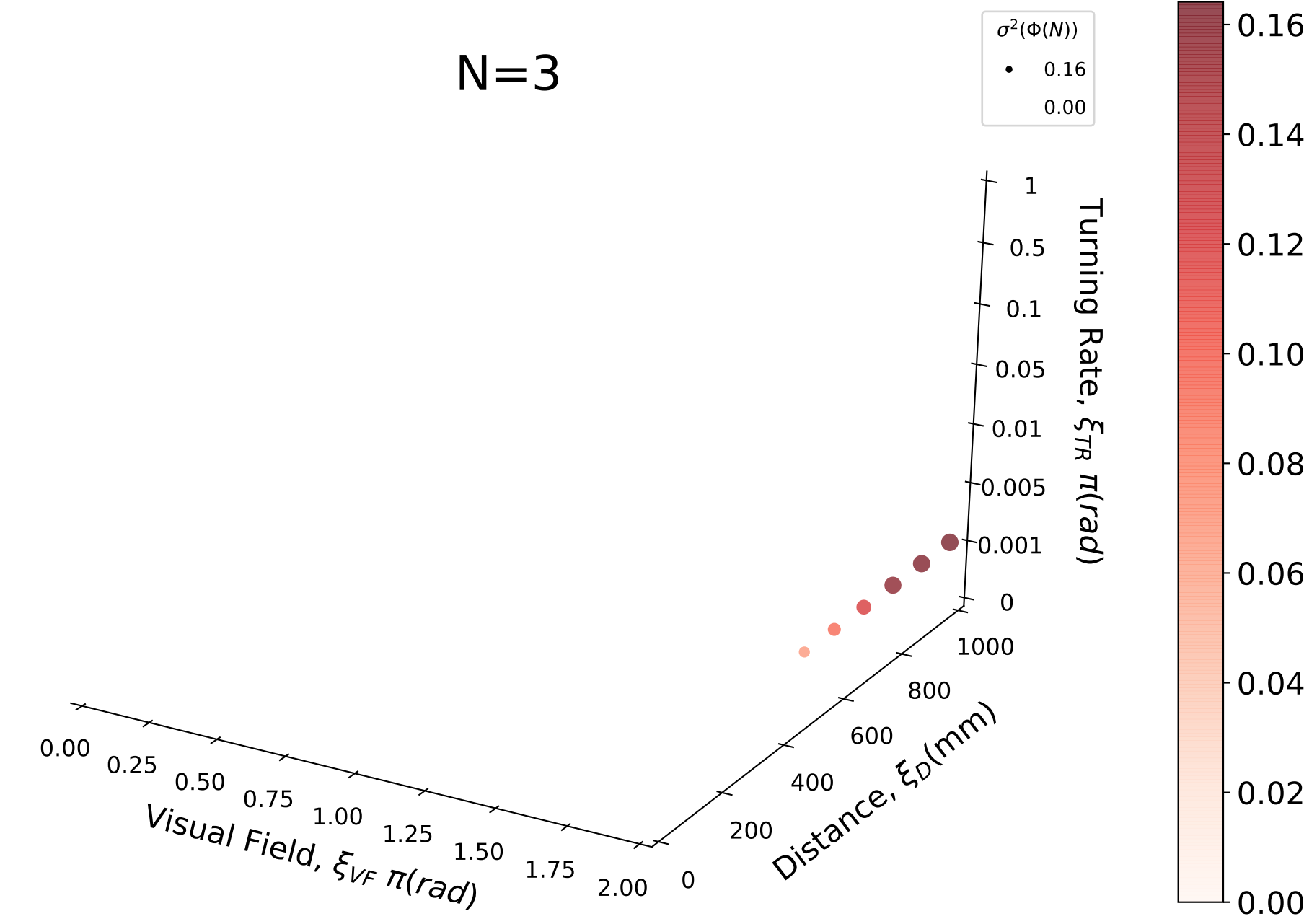

N=4

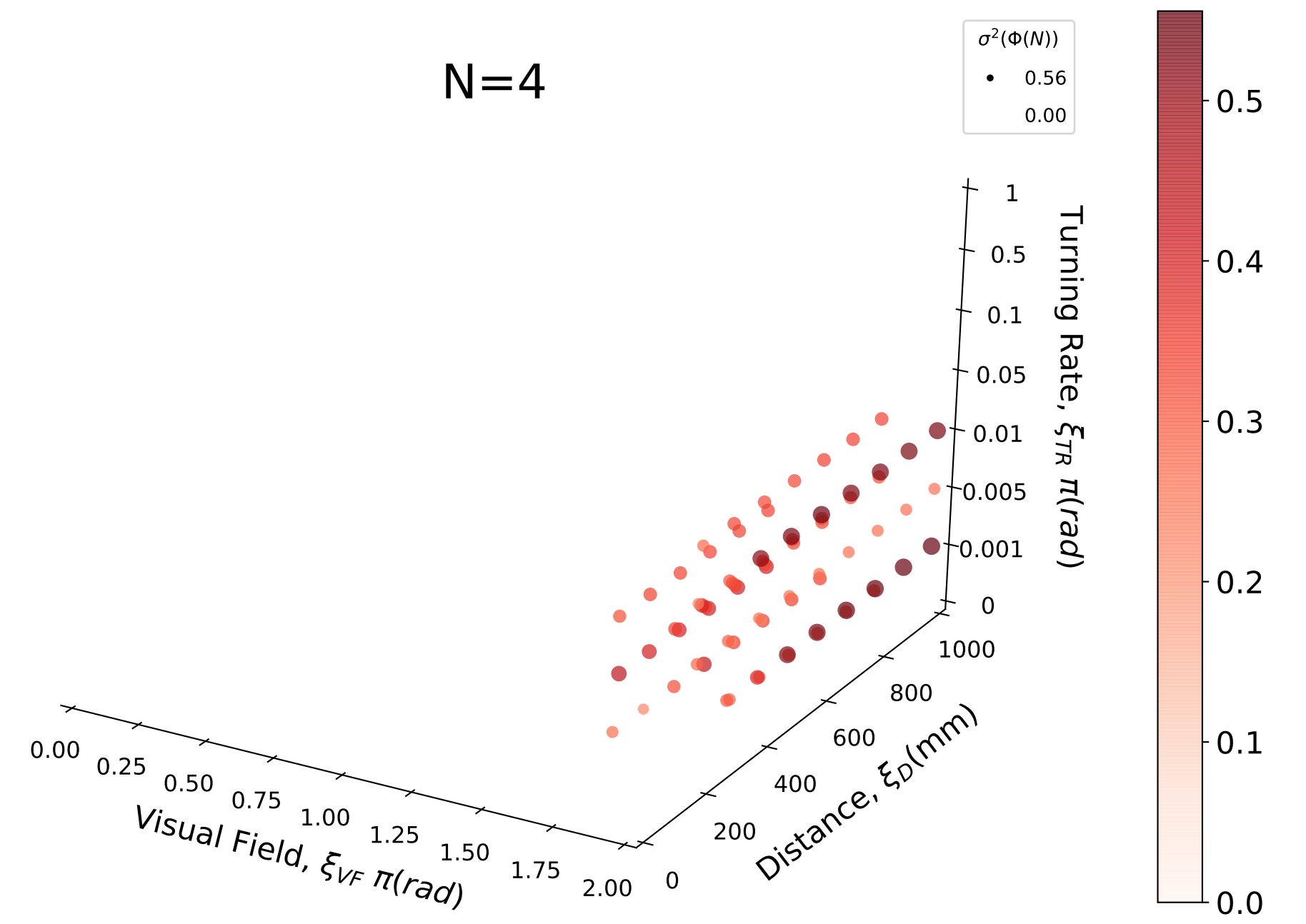

N=5

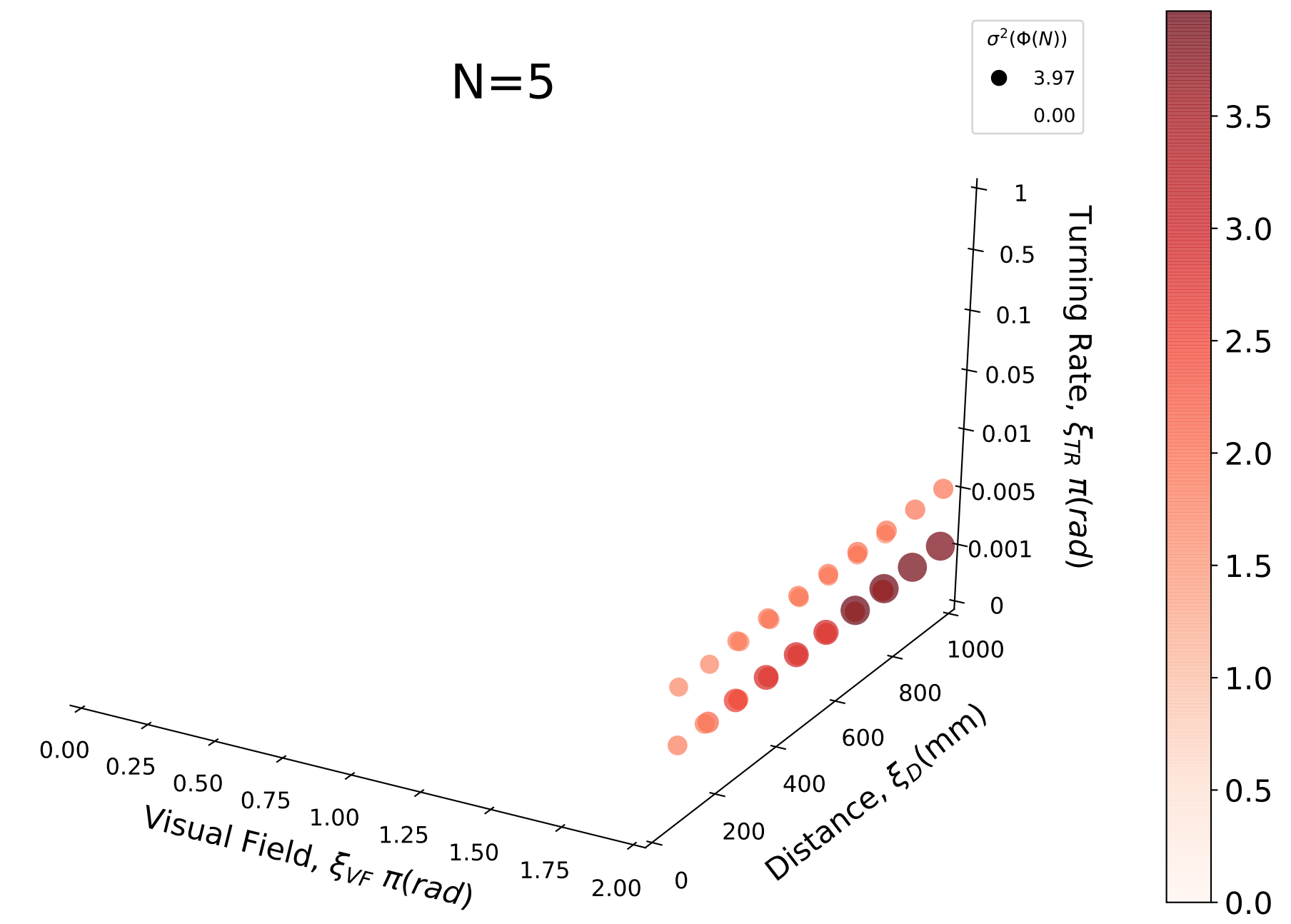

dt = 120/120 sec

N=2

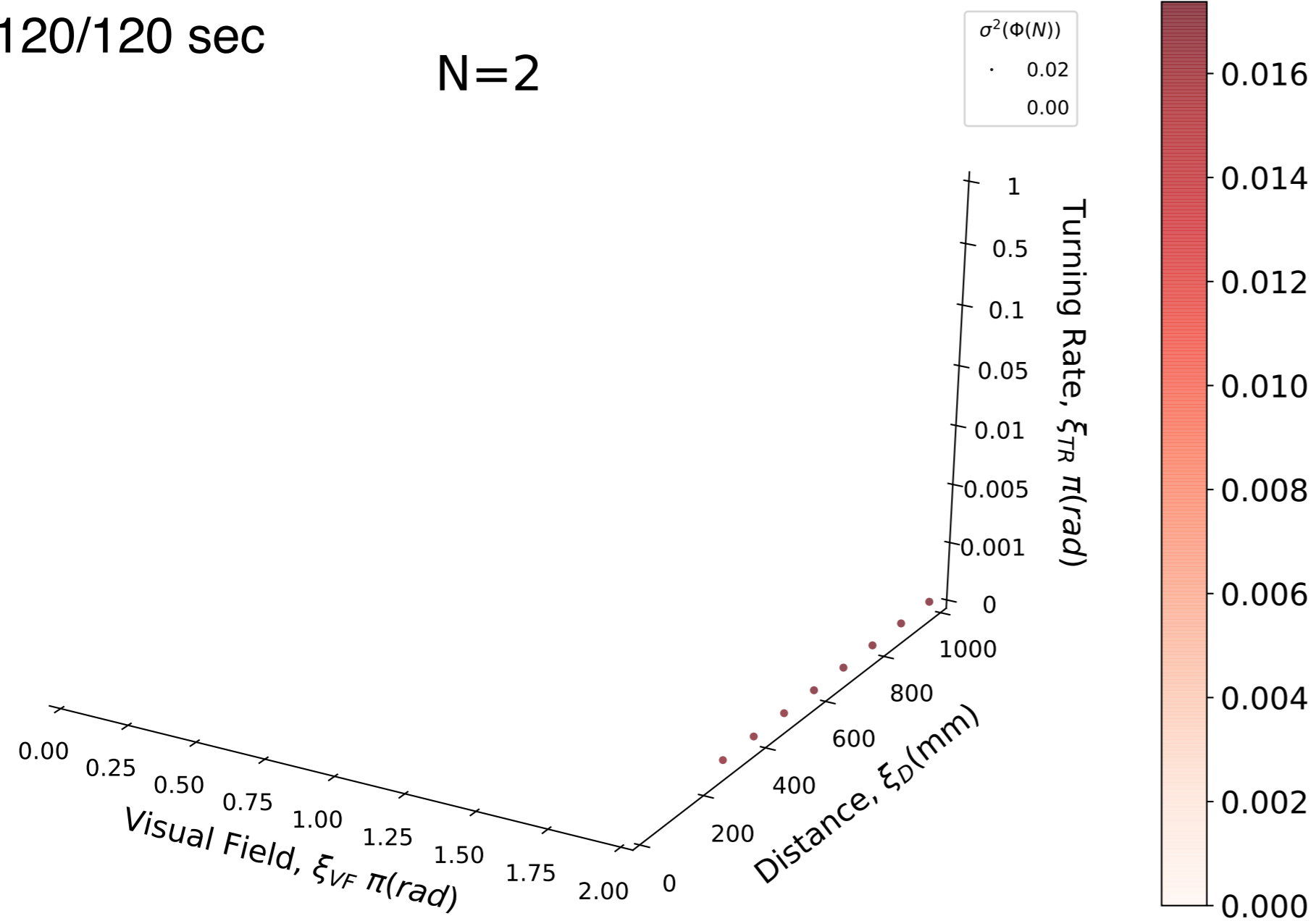

N=3

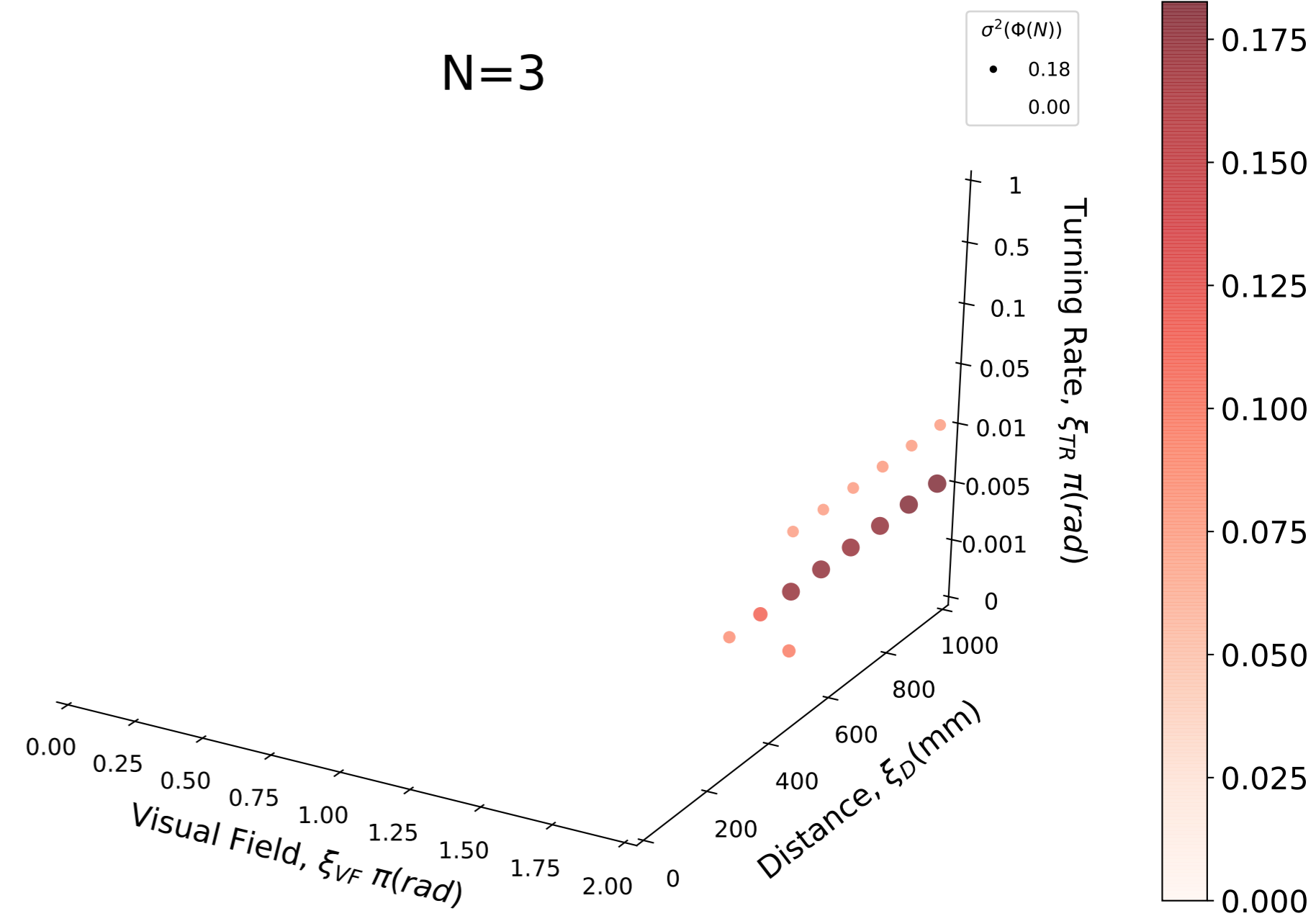

N=4

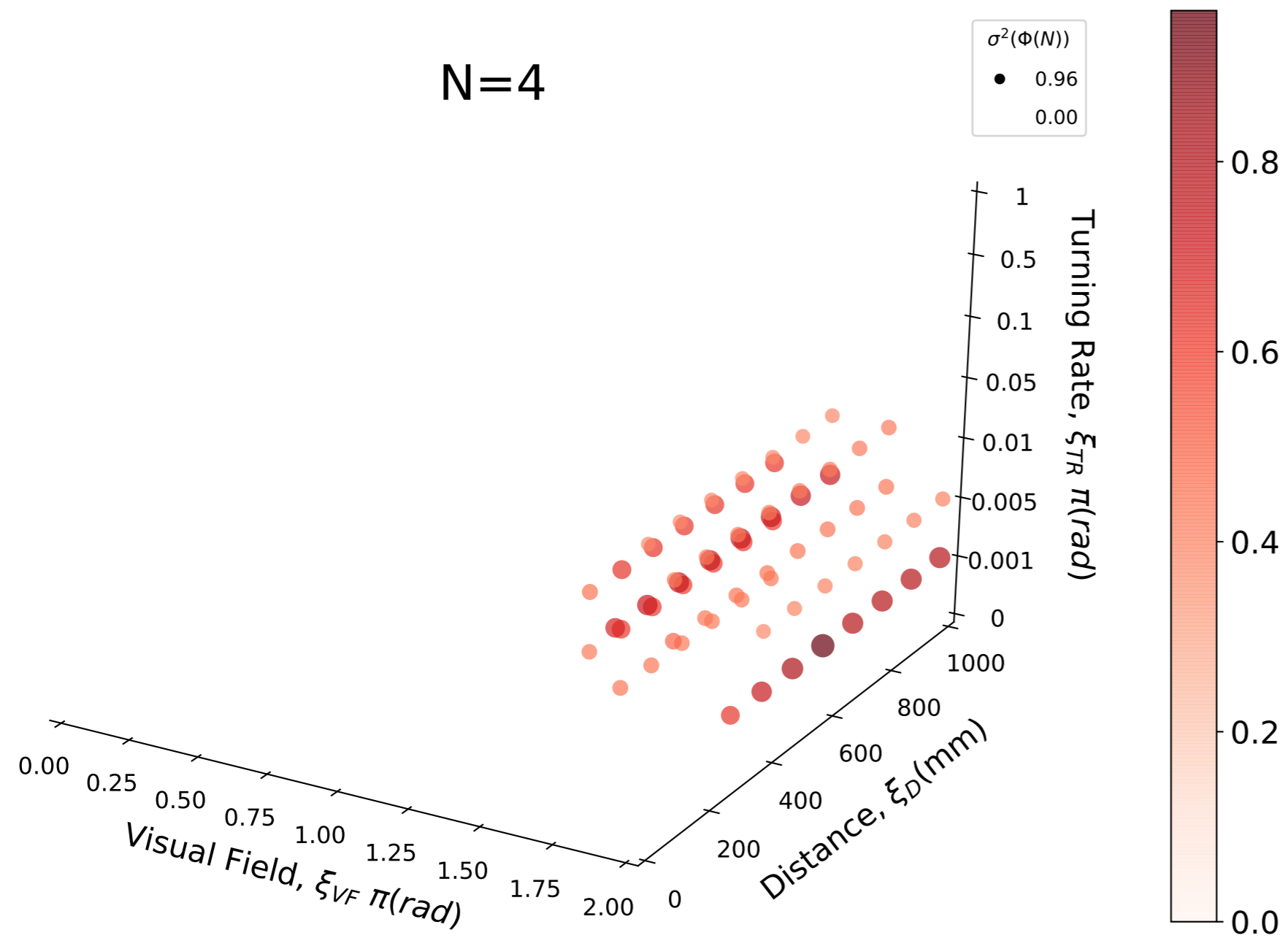

N=5

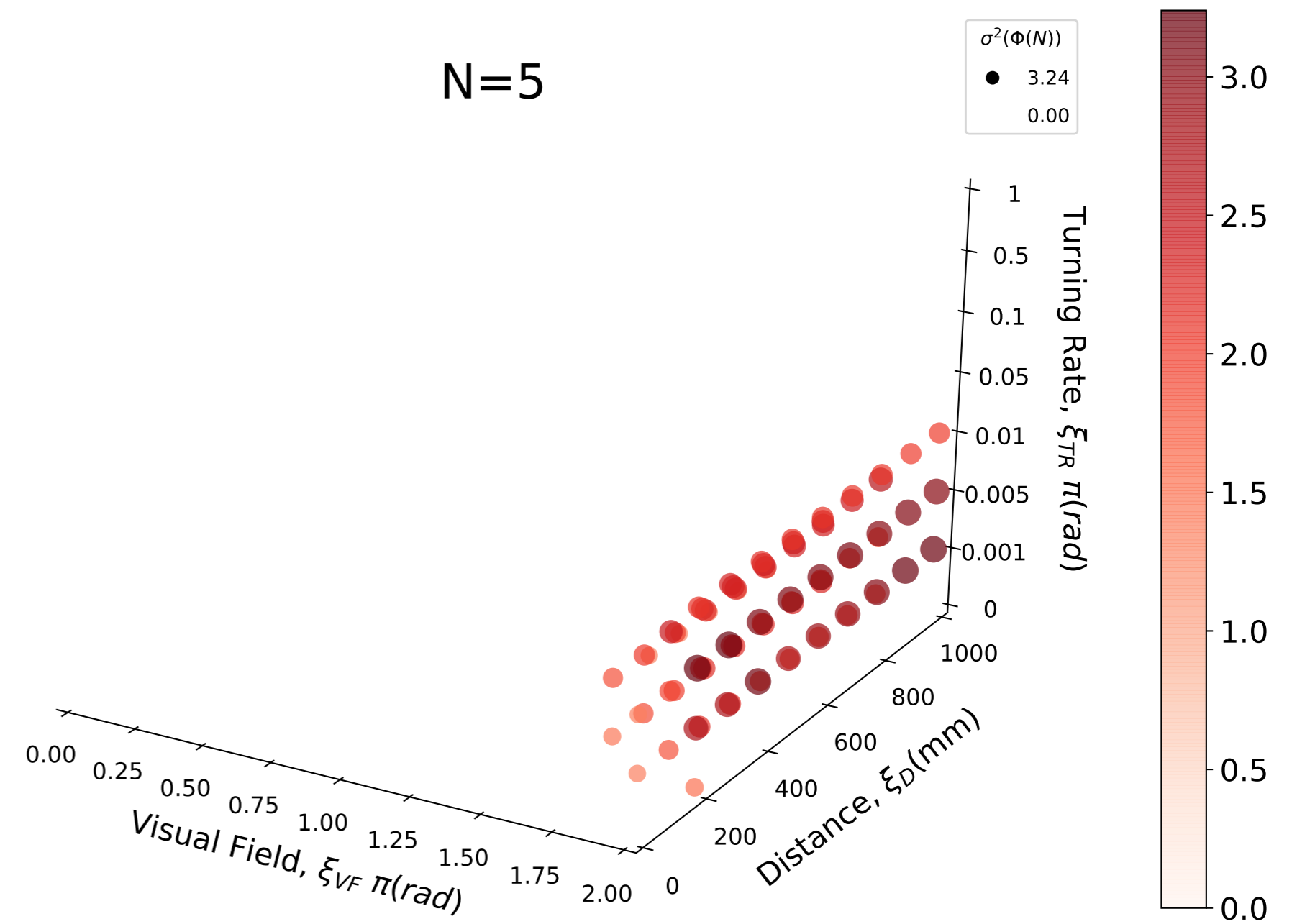

Supplement: Supplementary file 1 [file entropy-22-00726-s001.zip › Supporting Information/Figure_S2.pdf]

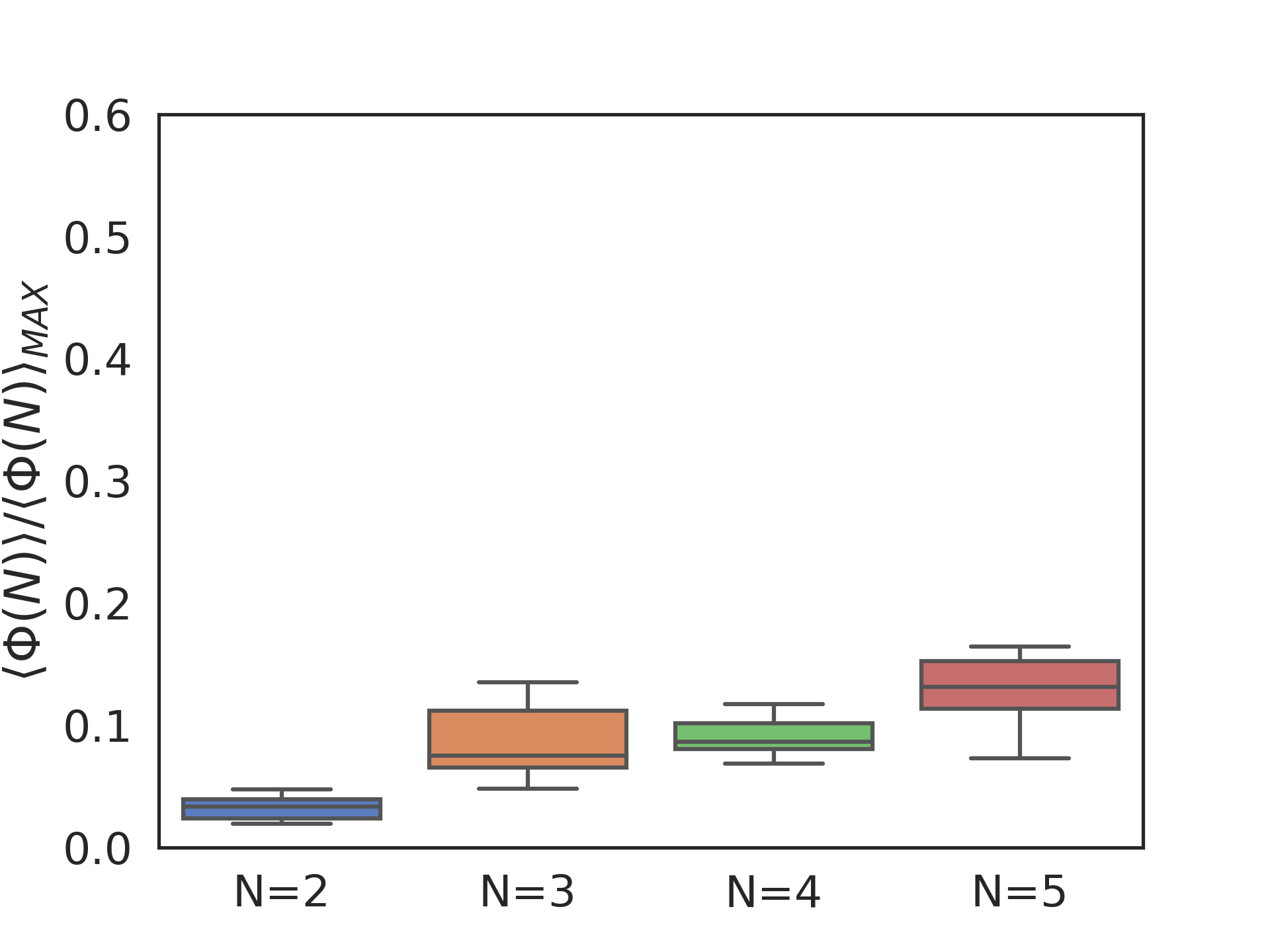

Supplement: Supplementary file 1 [file entropy-22-00726-s001.zip › Supporting Information/Figure_S3.png]

dt = 5/120 sec

N=2

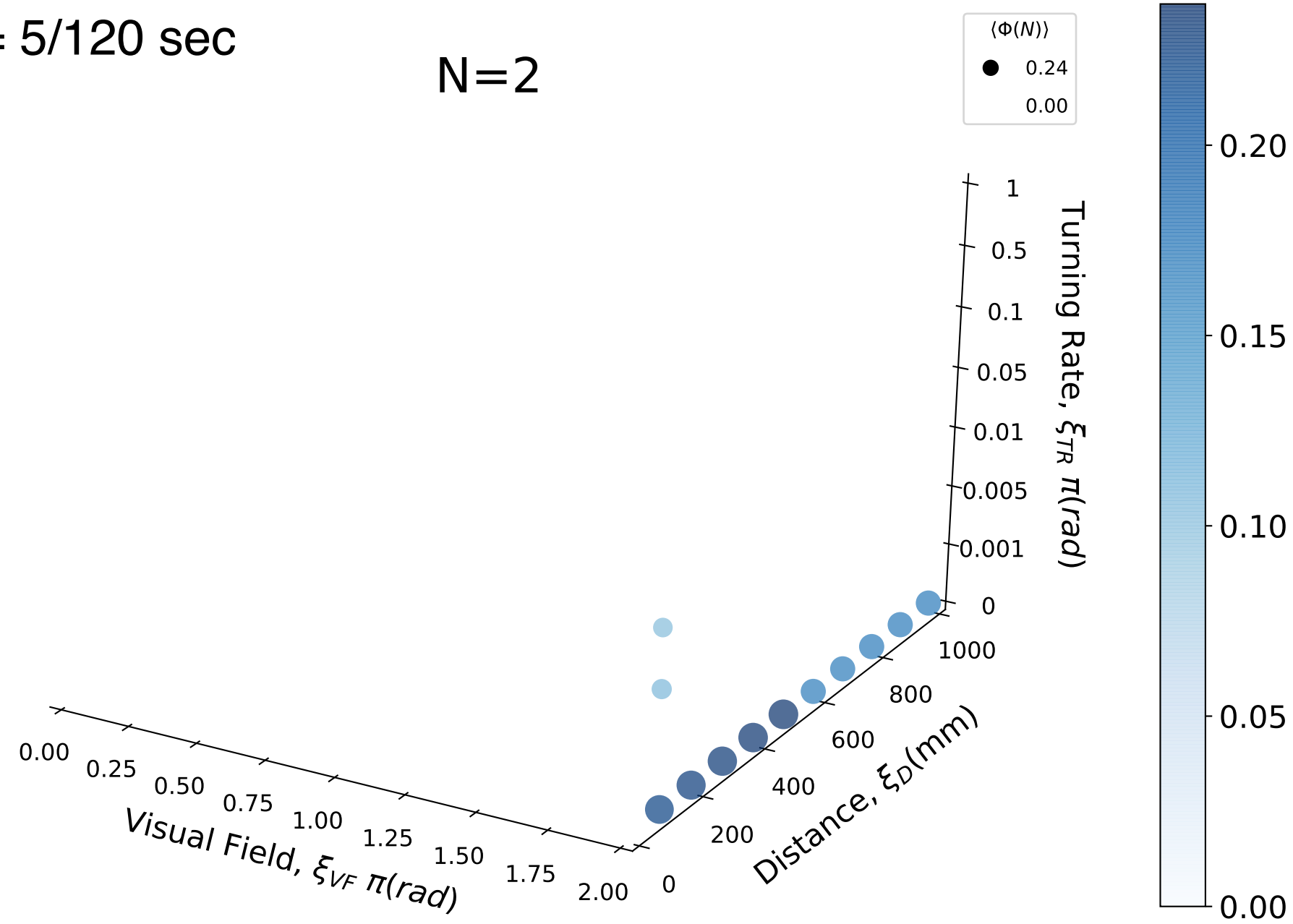

N=3

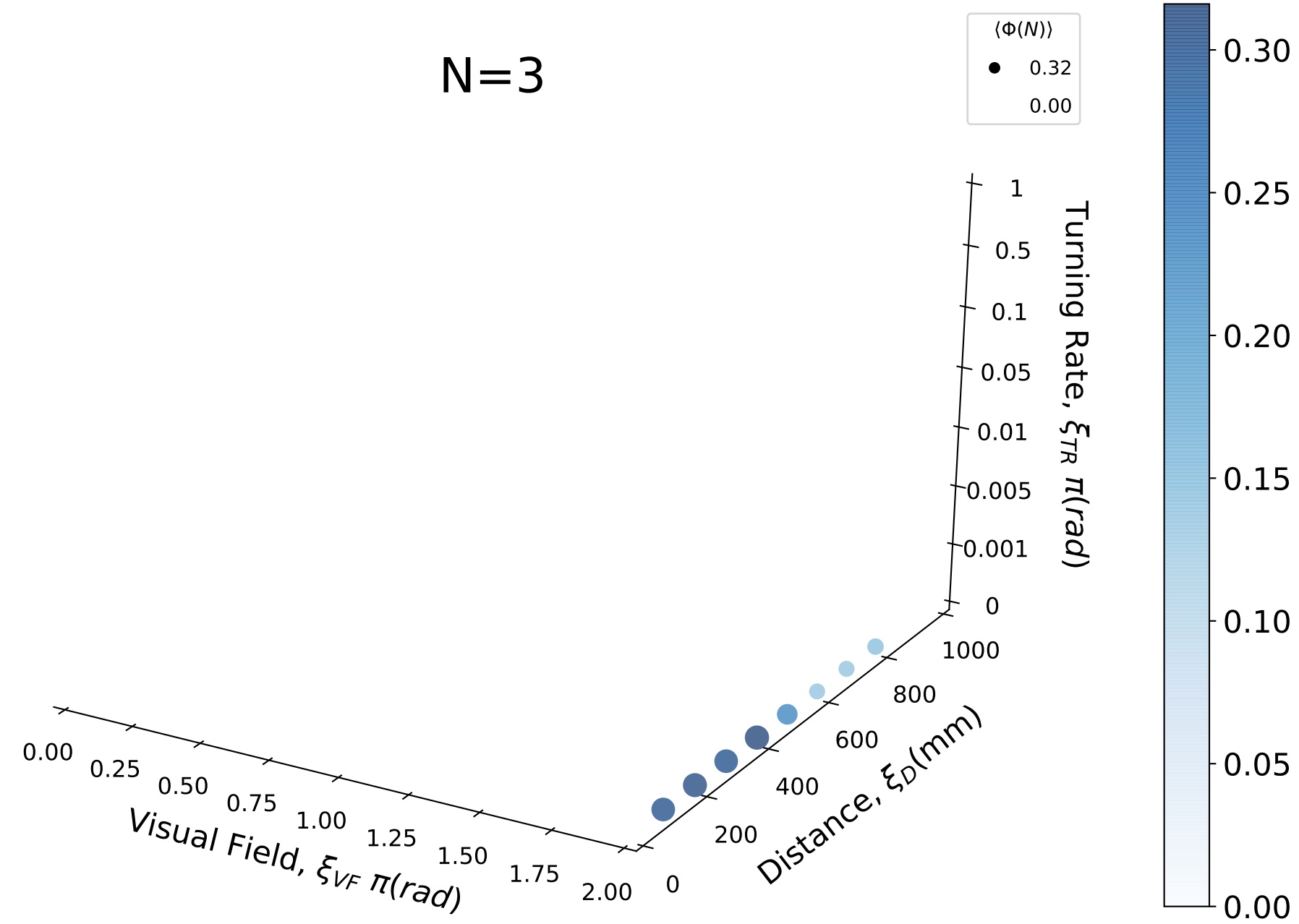

N=4

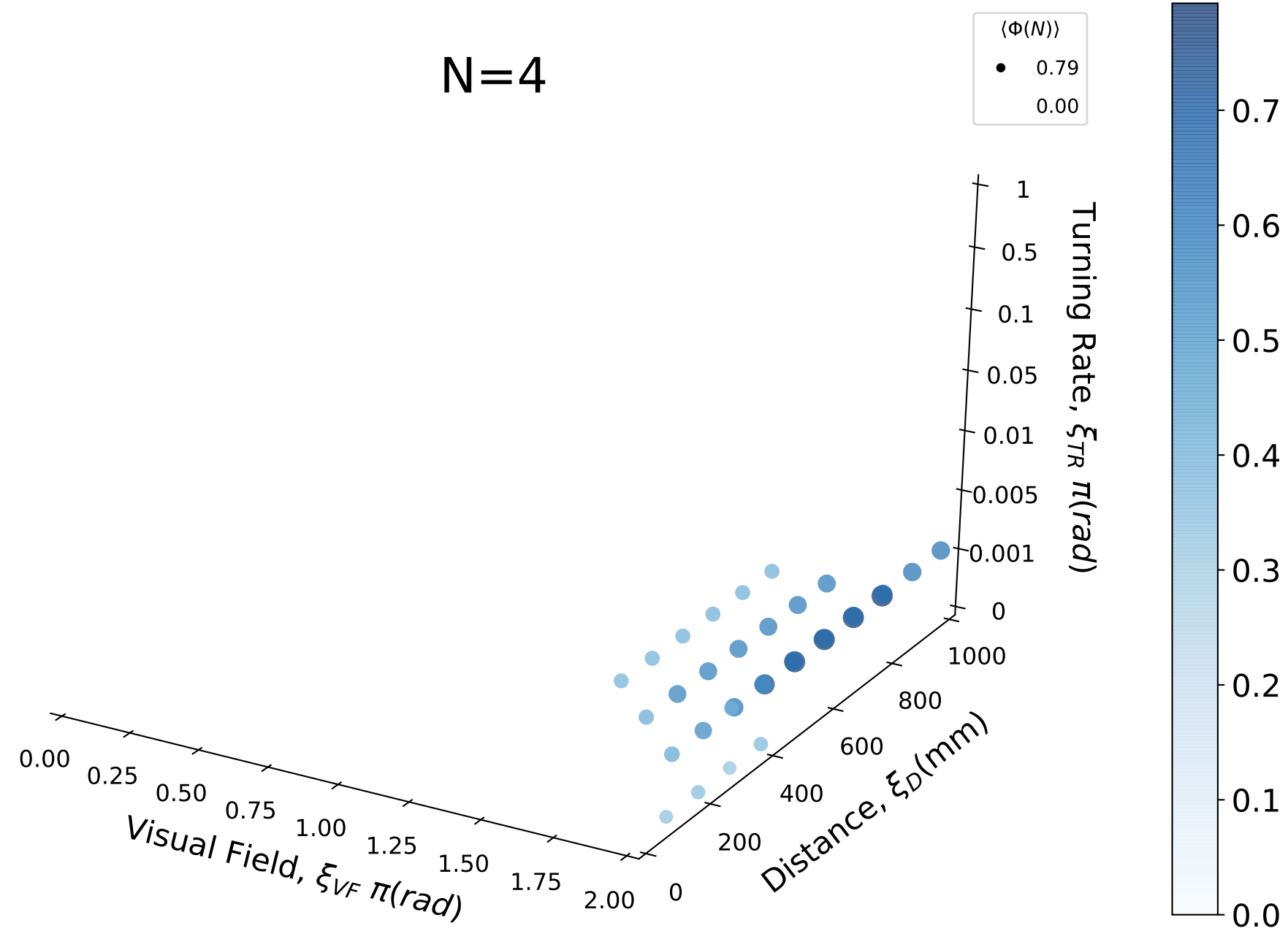

N=5

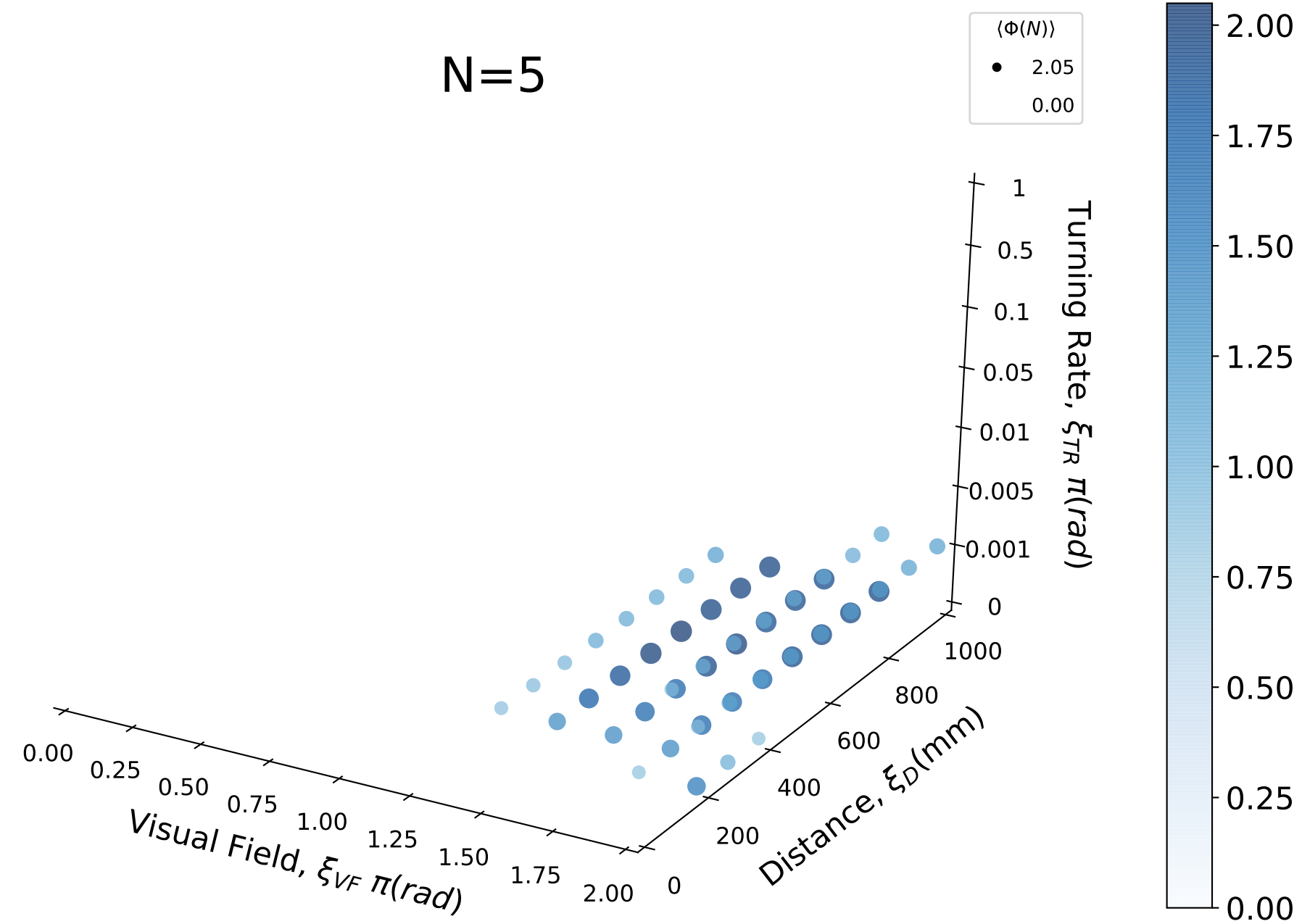

dt = 10/120 sec

N=2

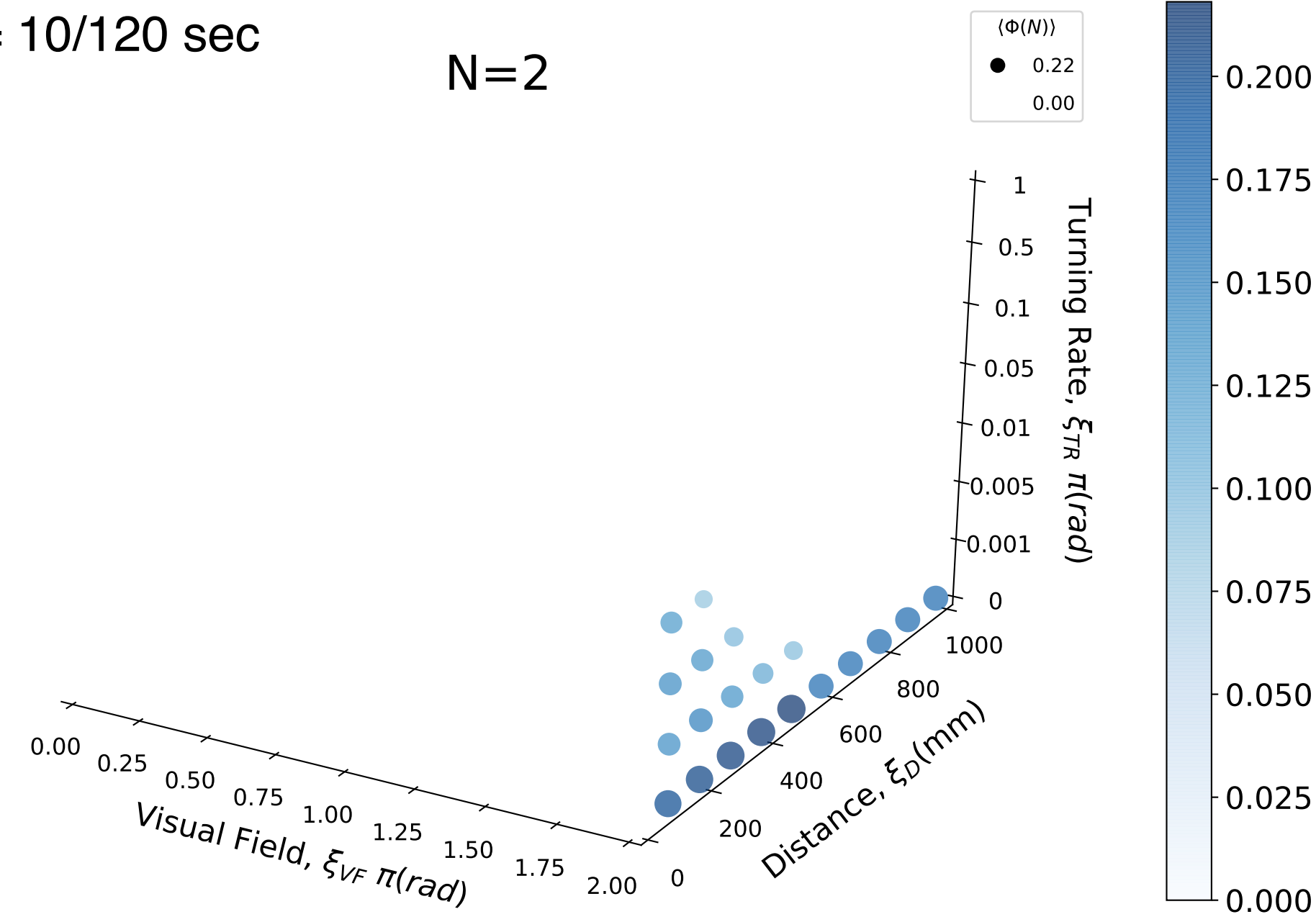

N=3

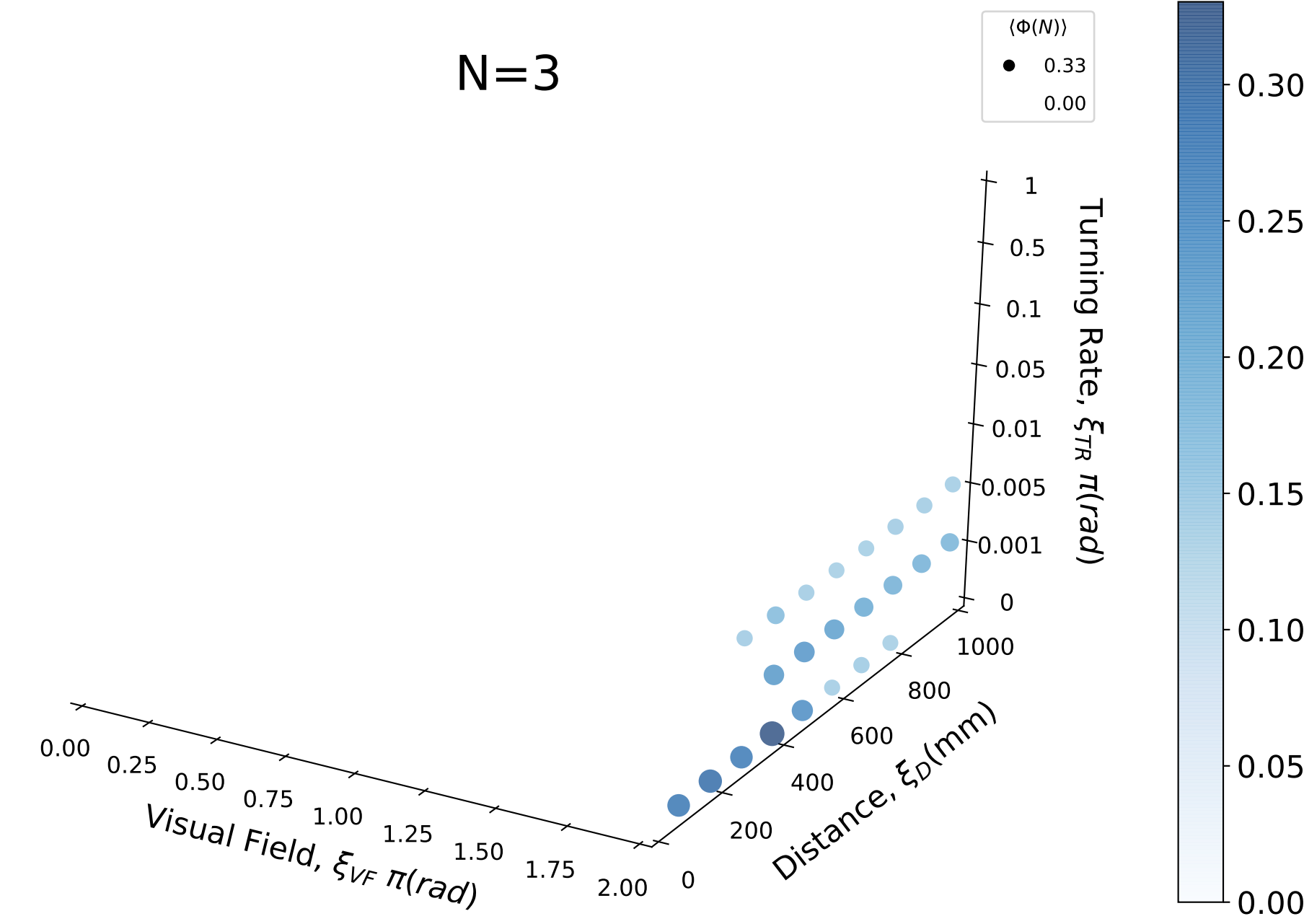

N=4

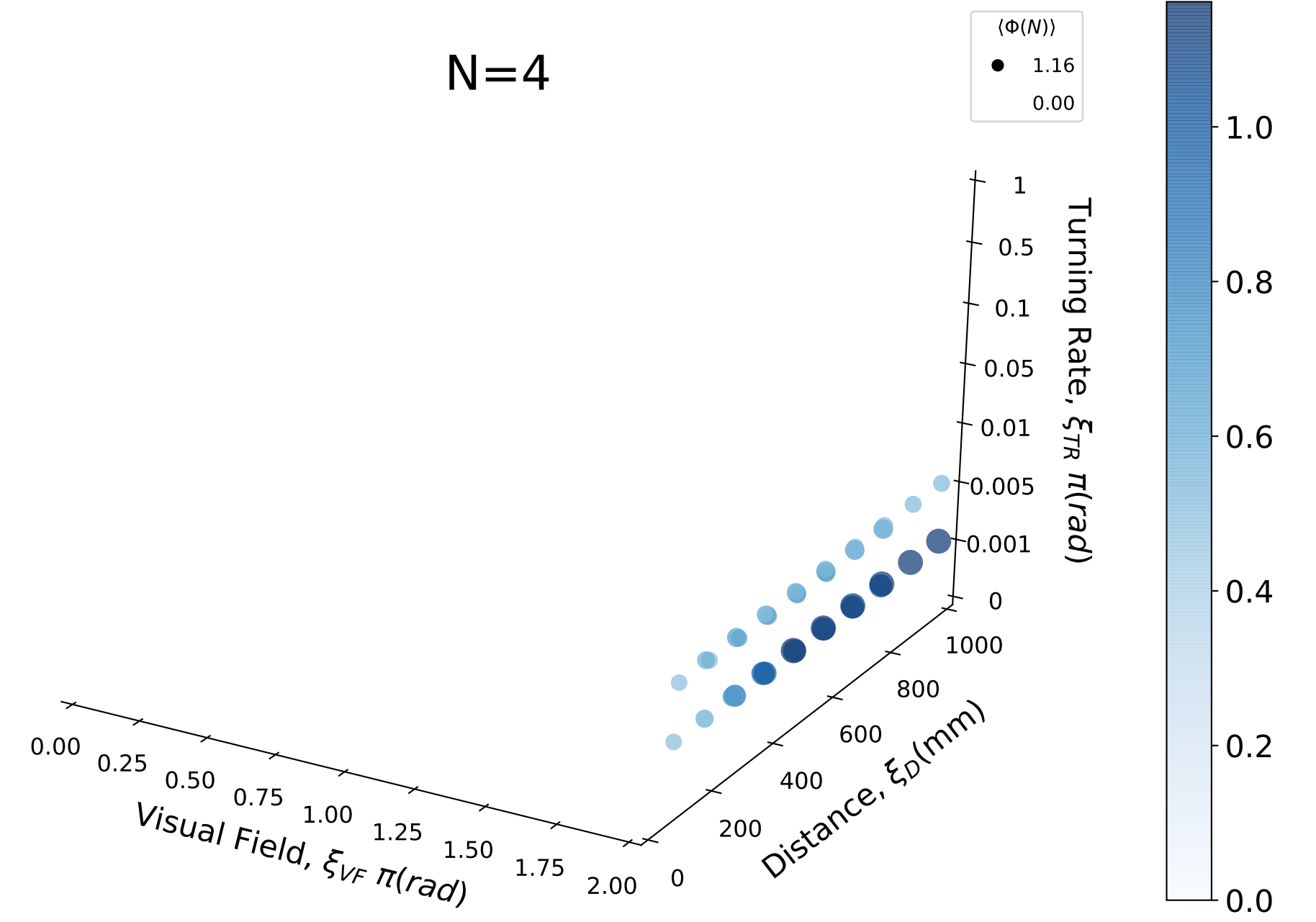

N=5

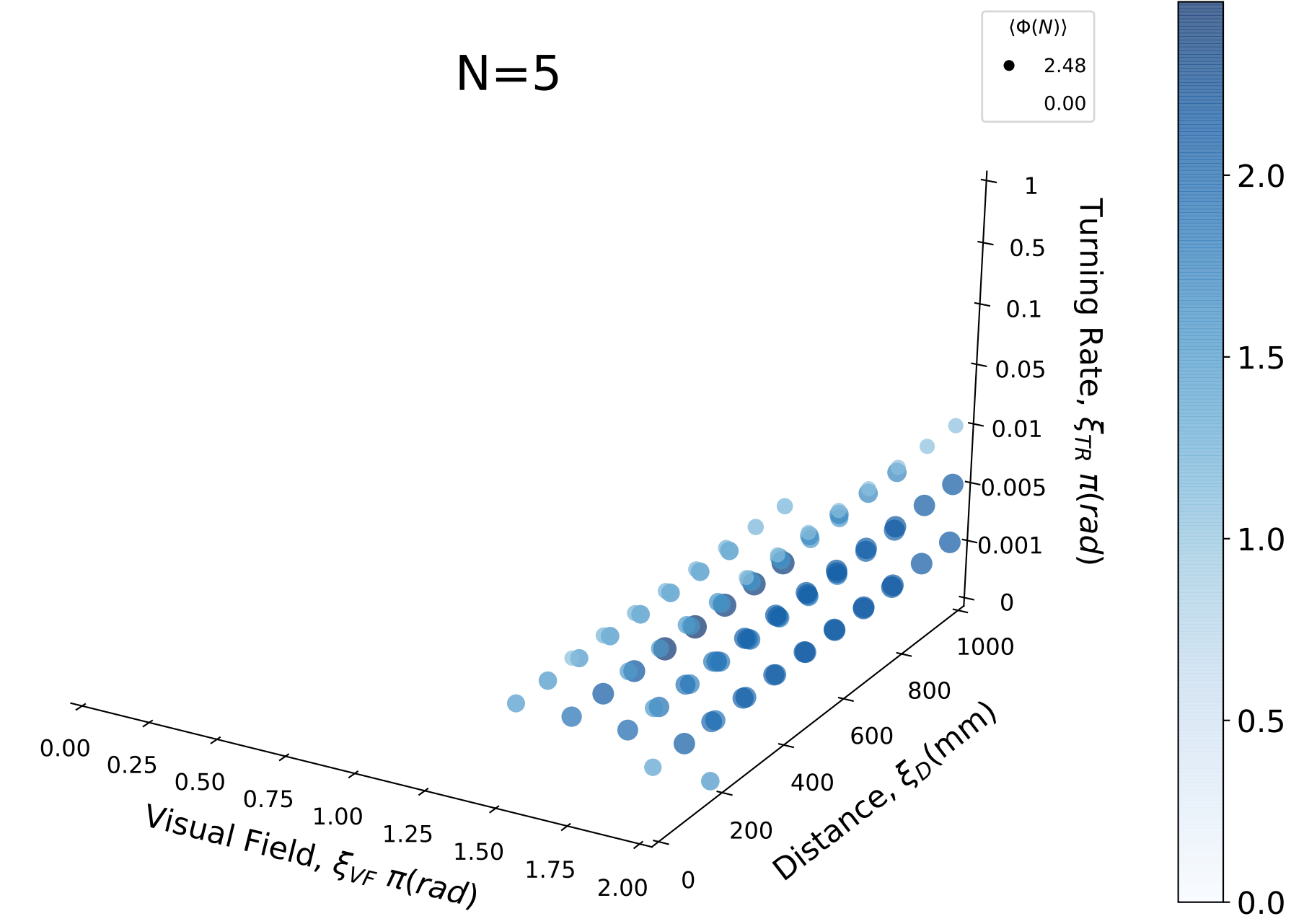

dt = 20/120 sec

N=2

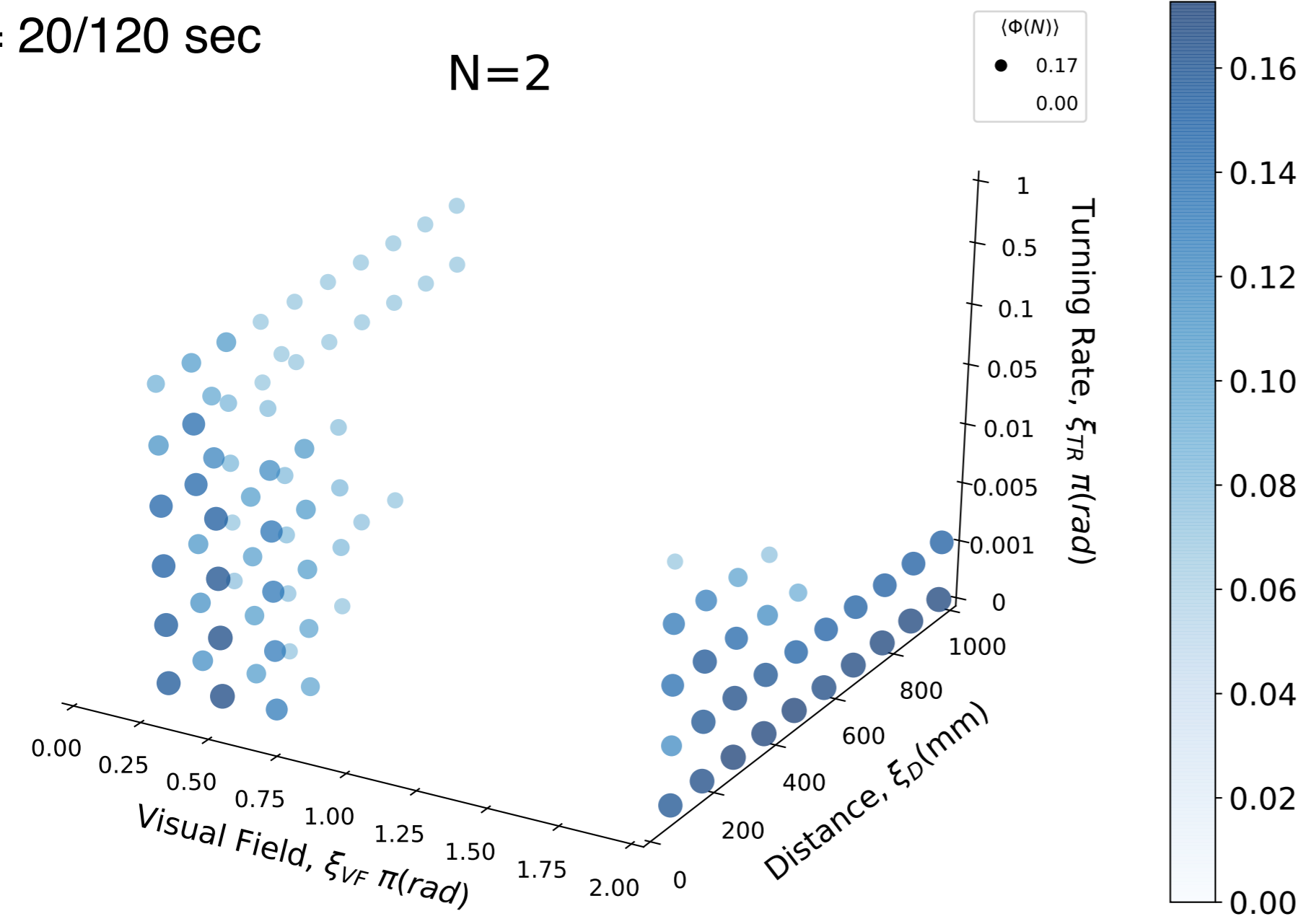

N=3

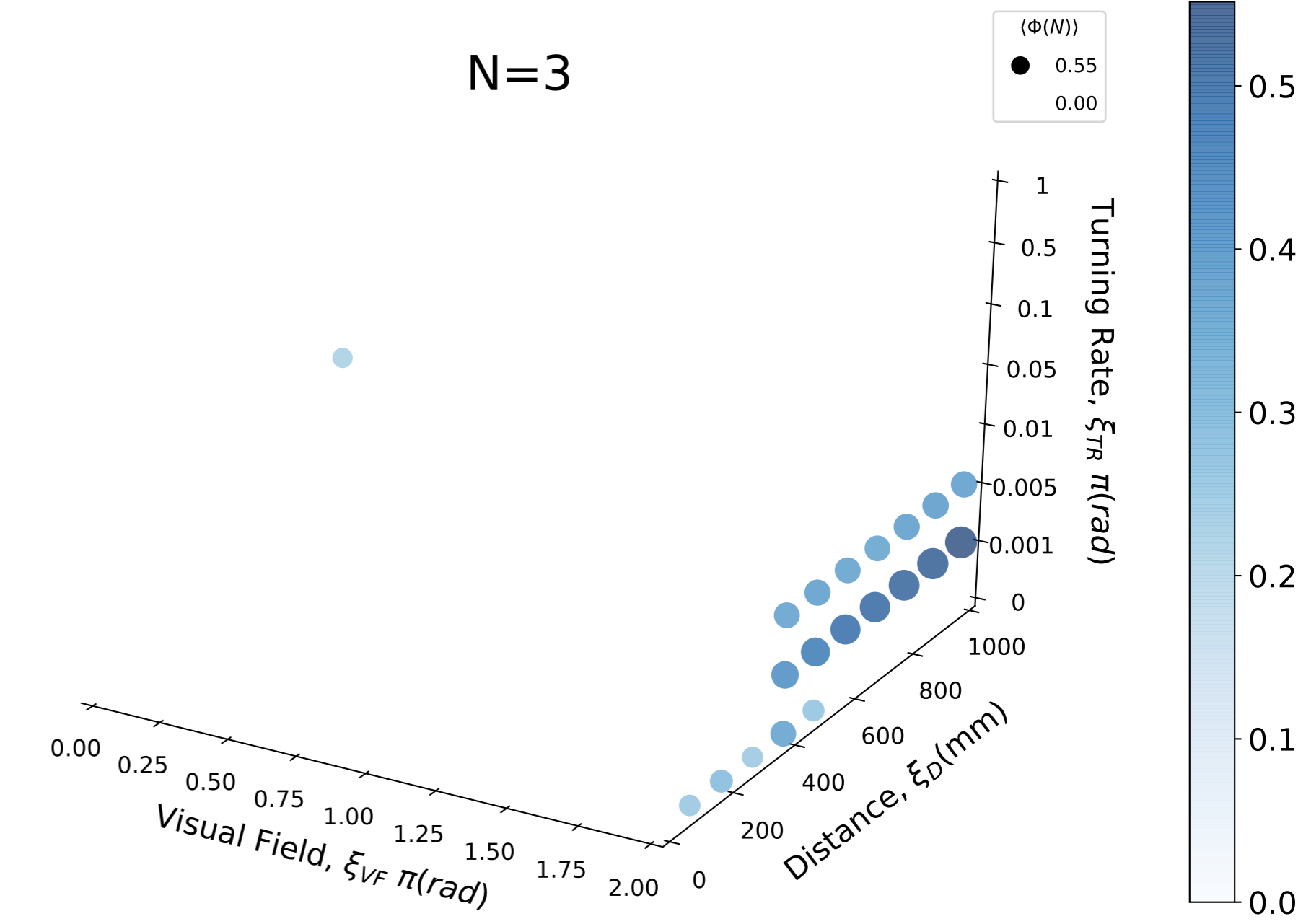

N=4

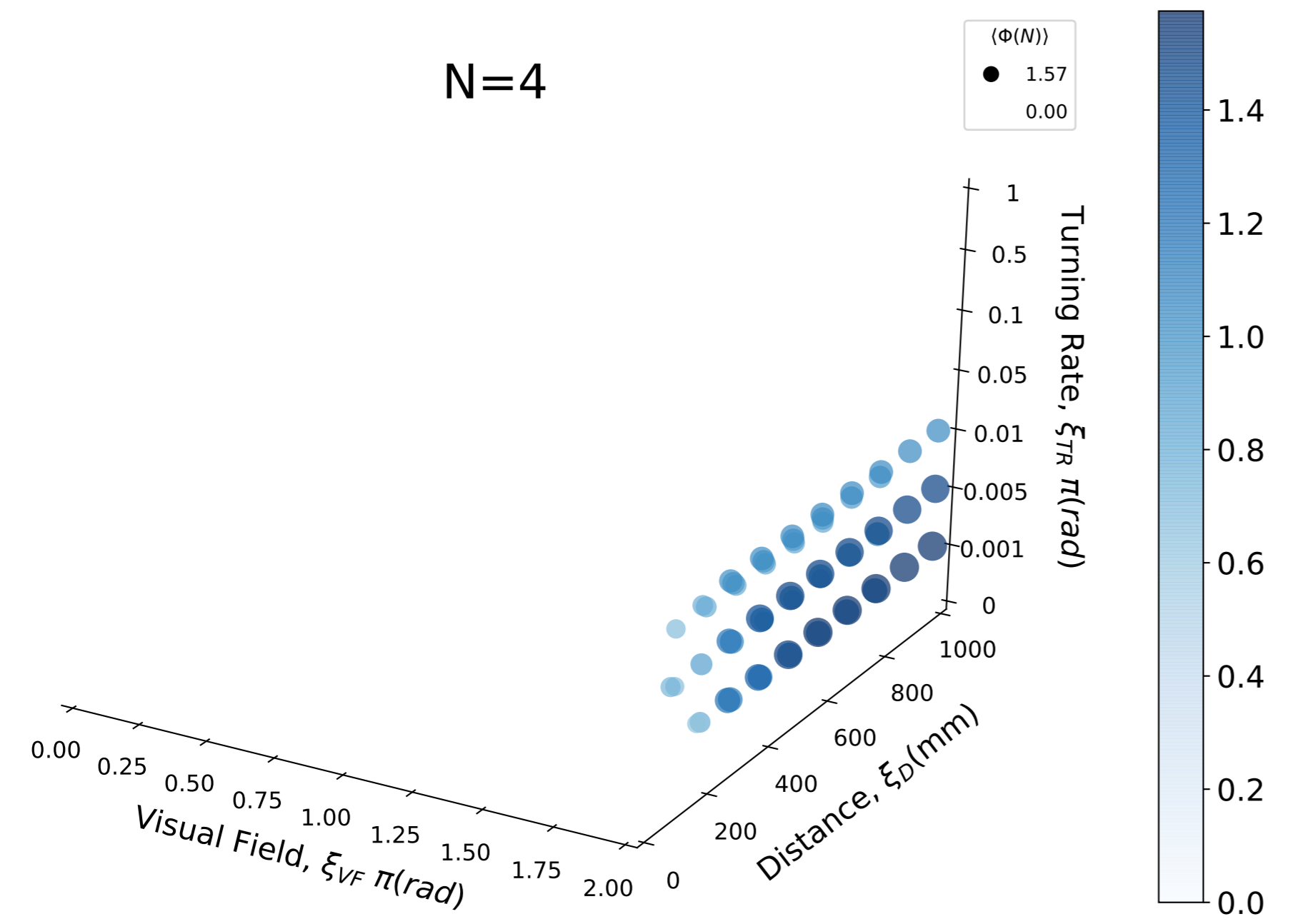

N=5

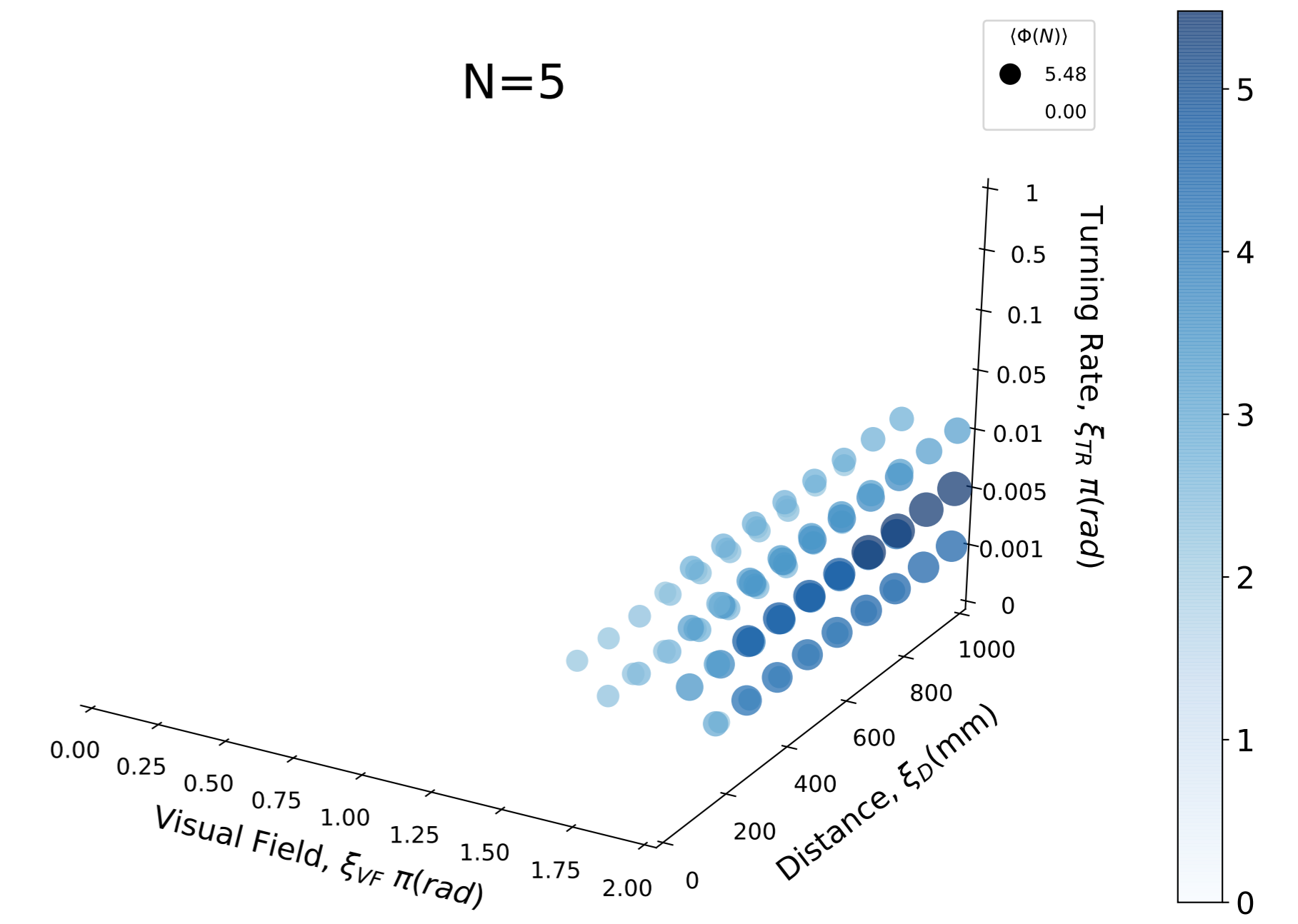

dt = 40/120 sec

N=2

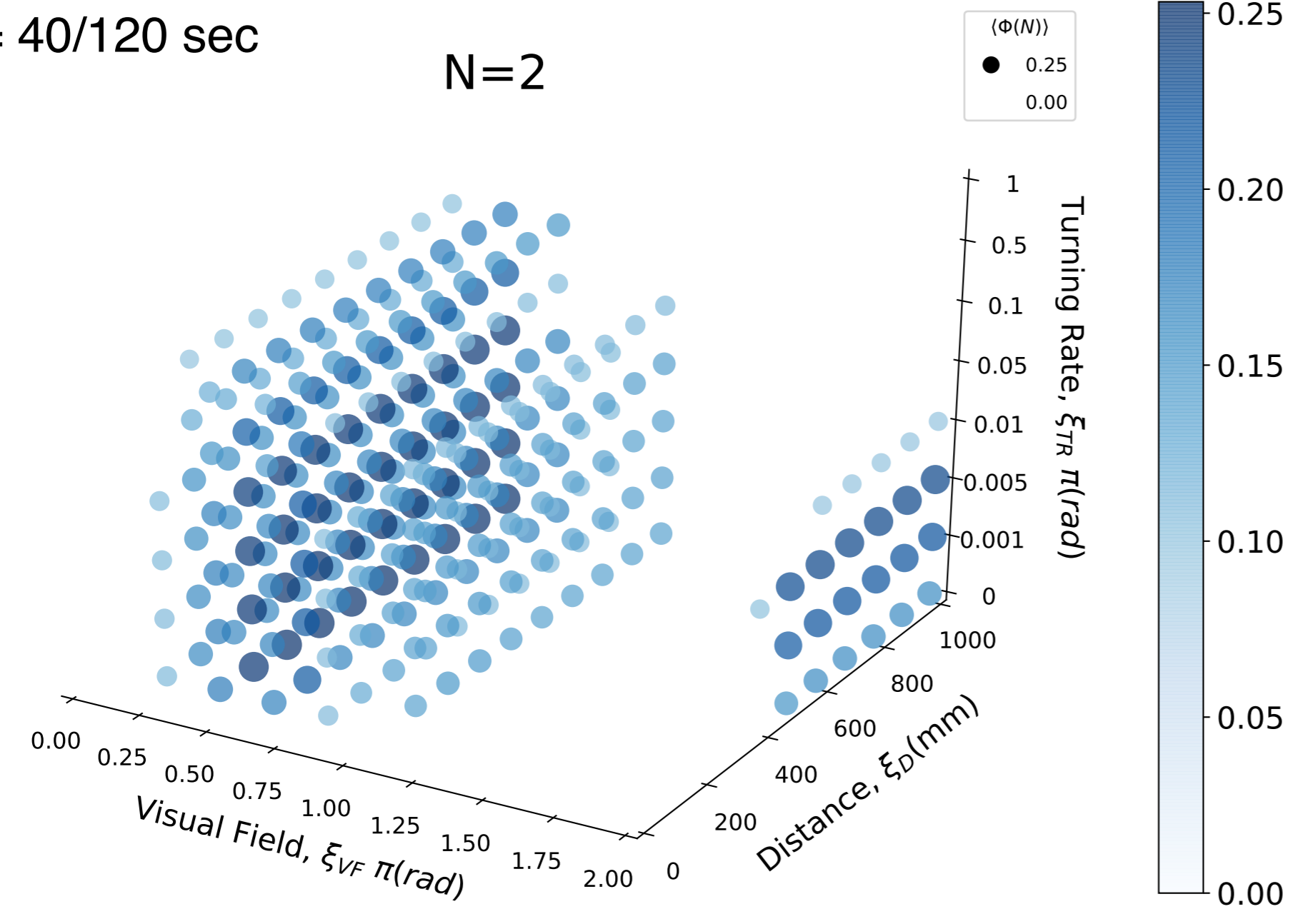

N=3

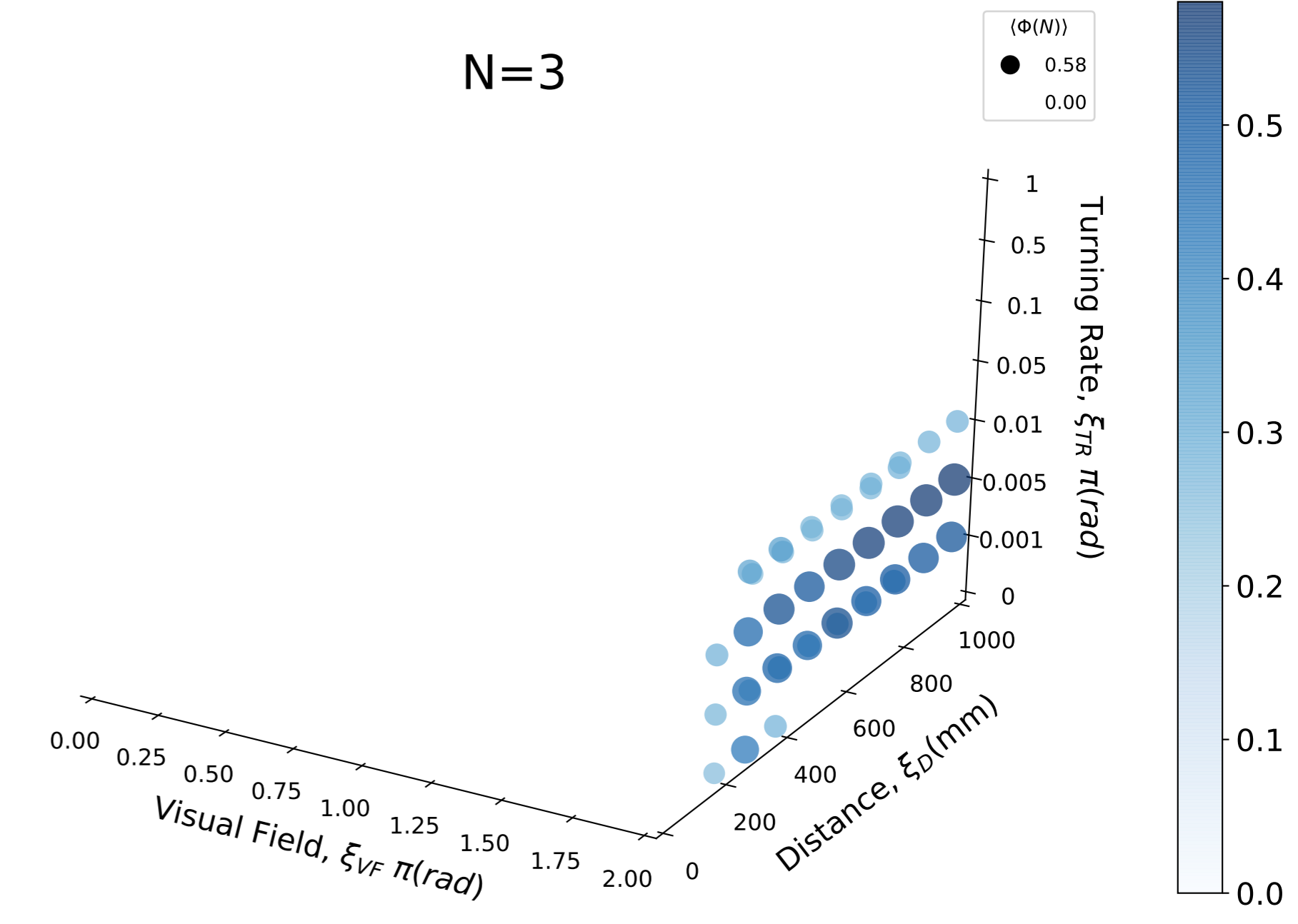

N=4

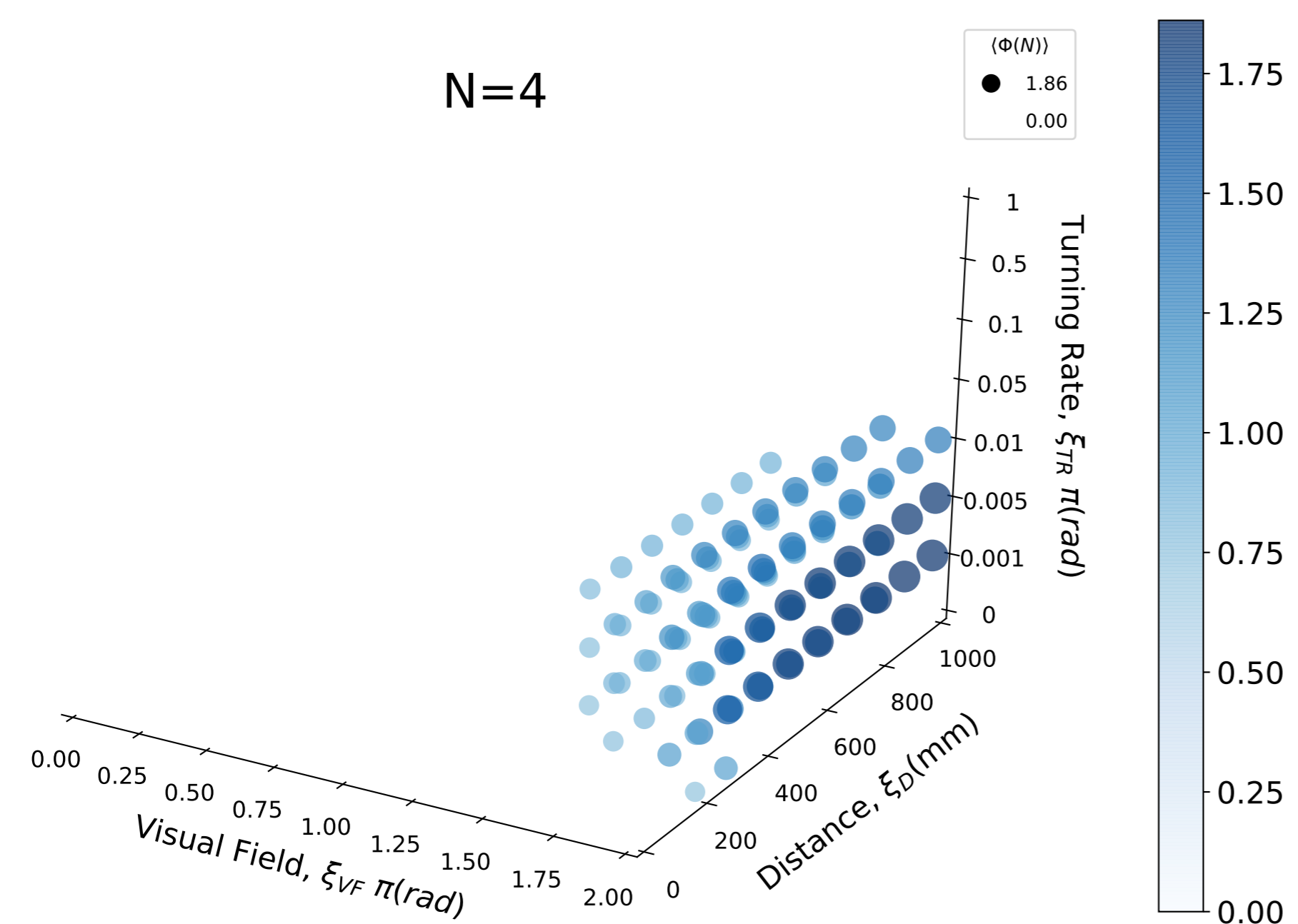

N=5

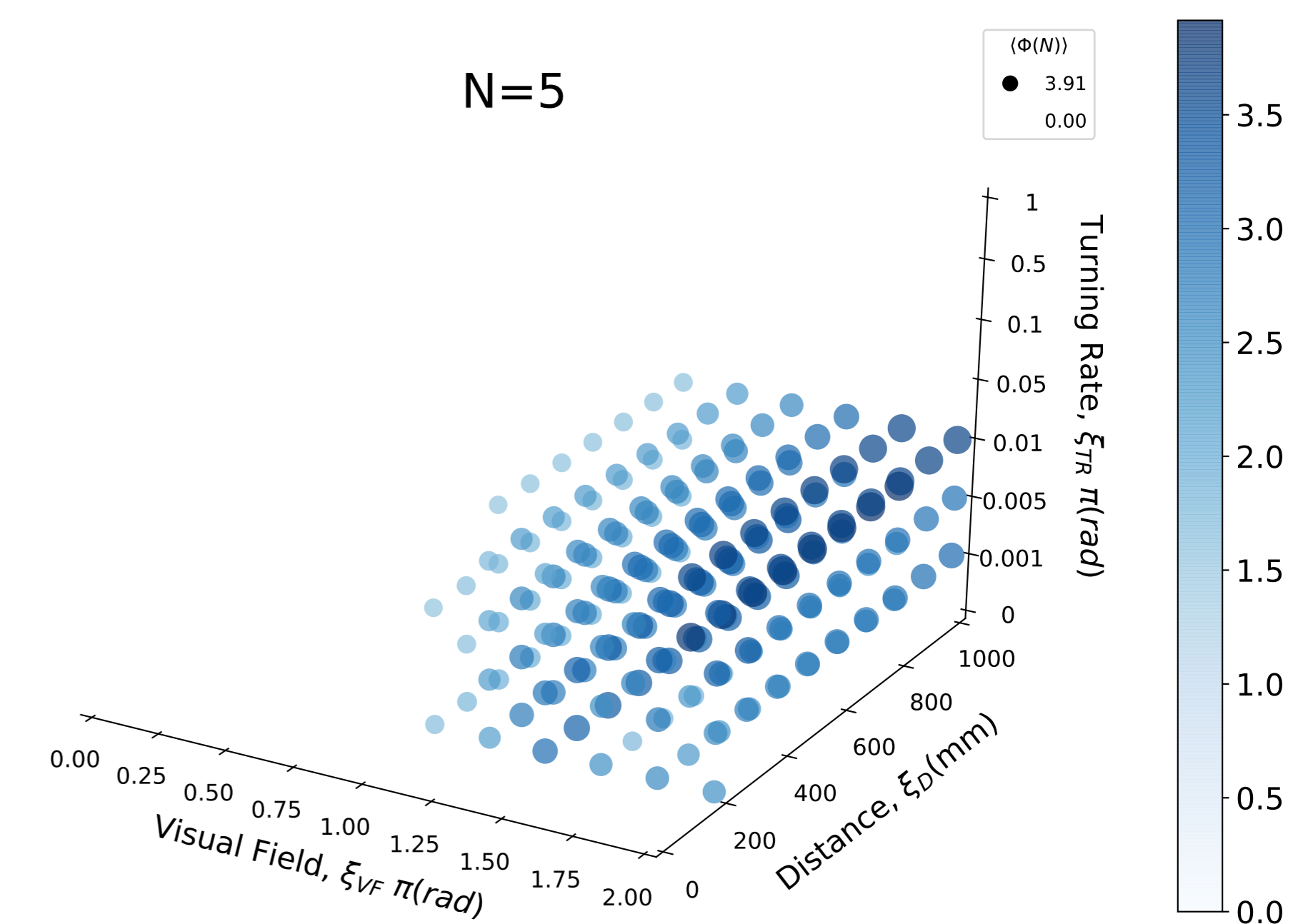

dt = 80/120 sec

N=2

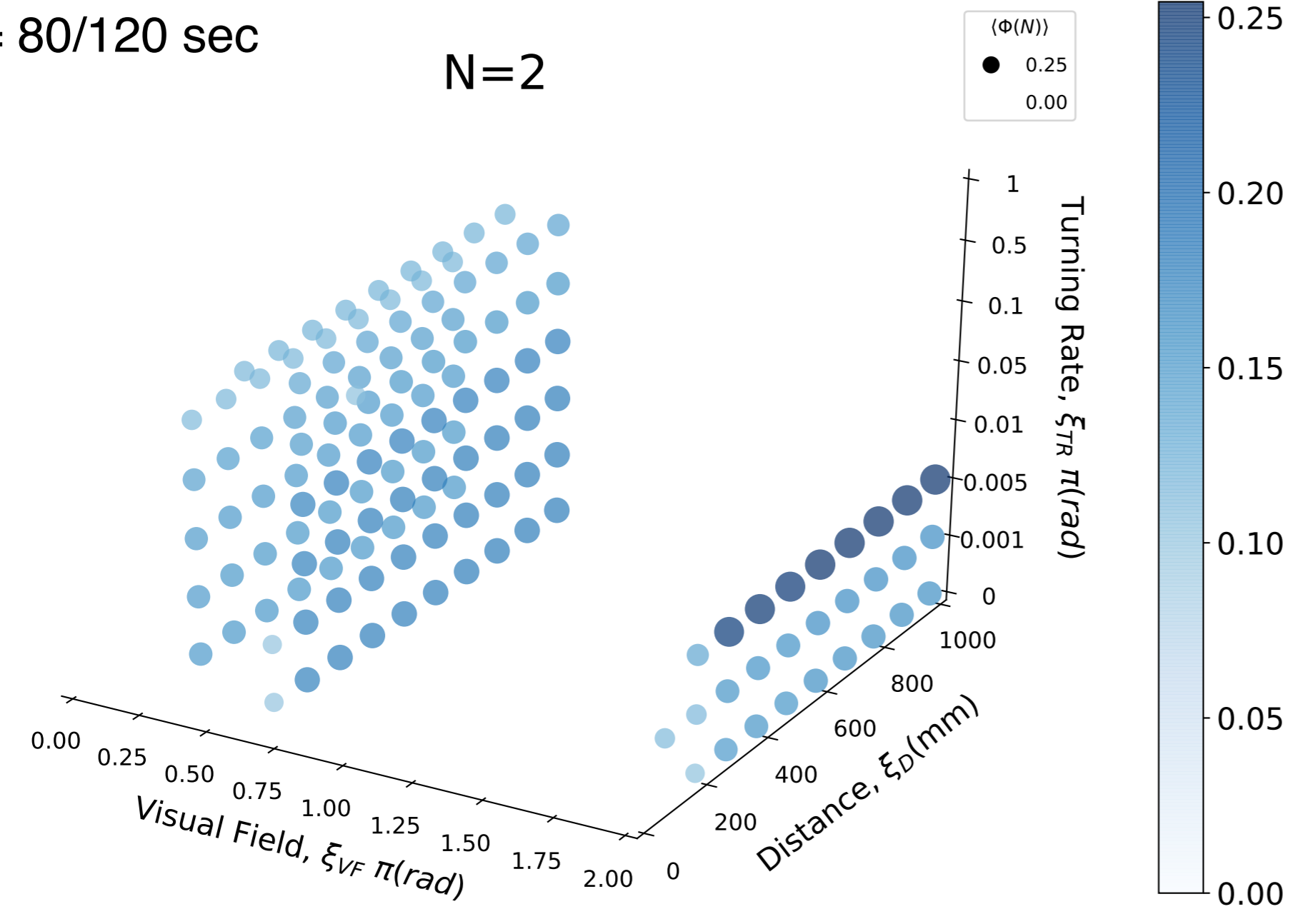

N=3

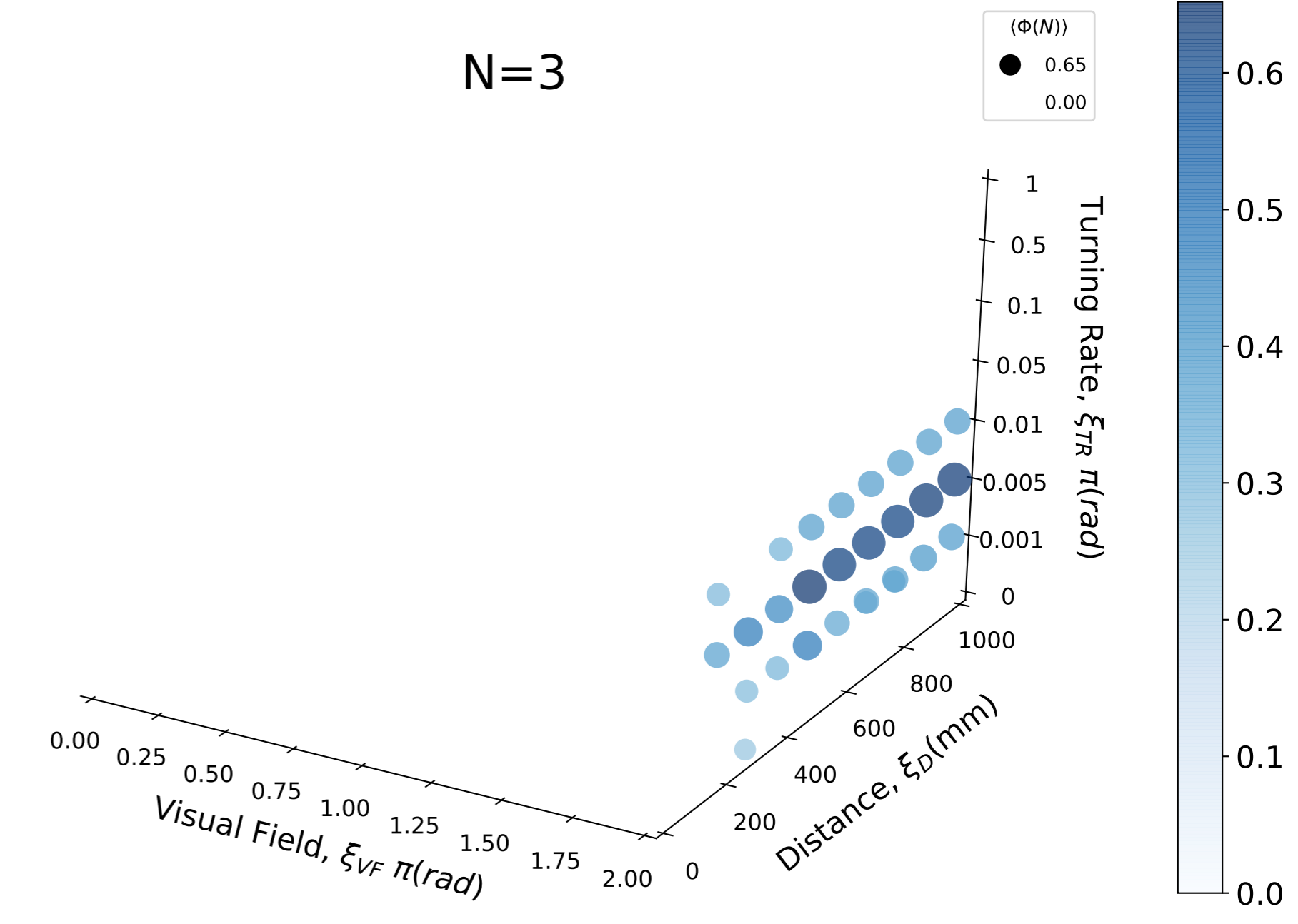

N=4

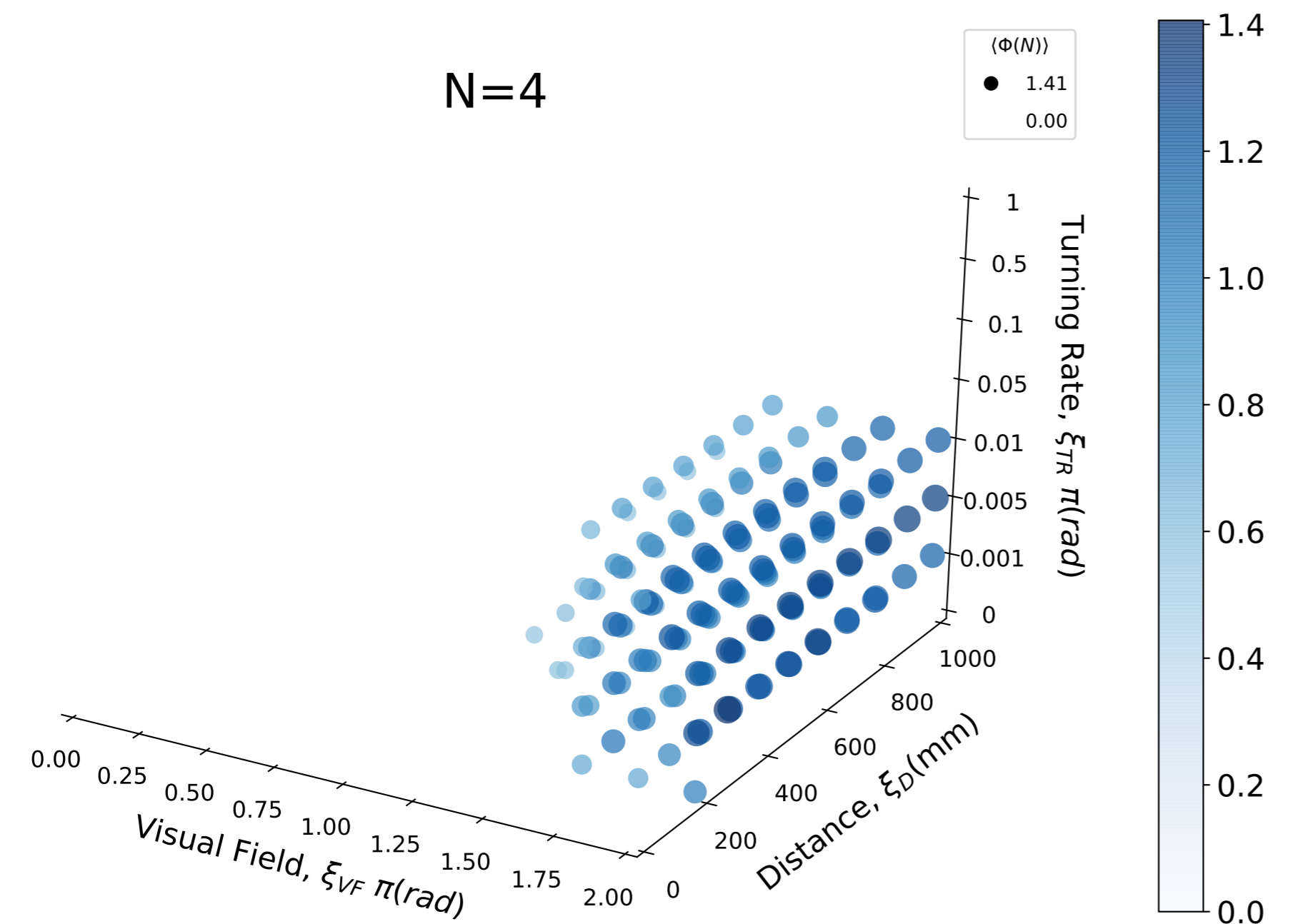

N=5

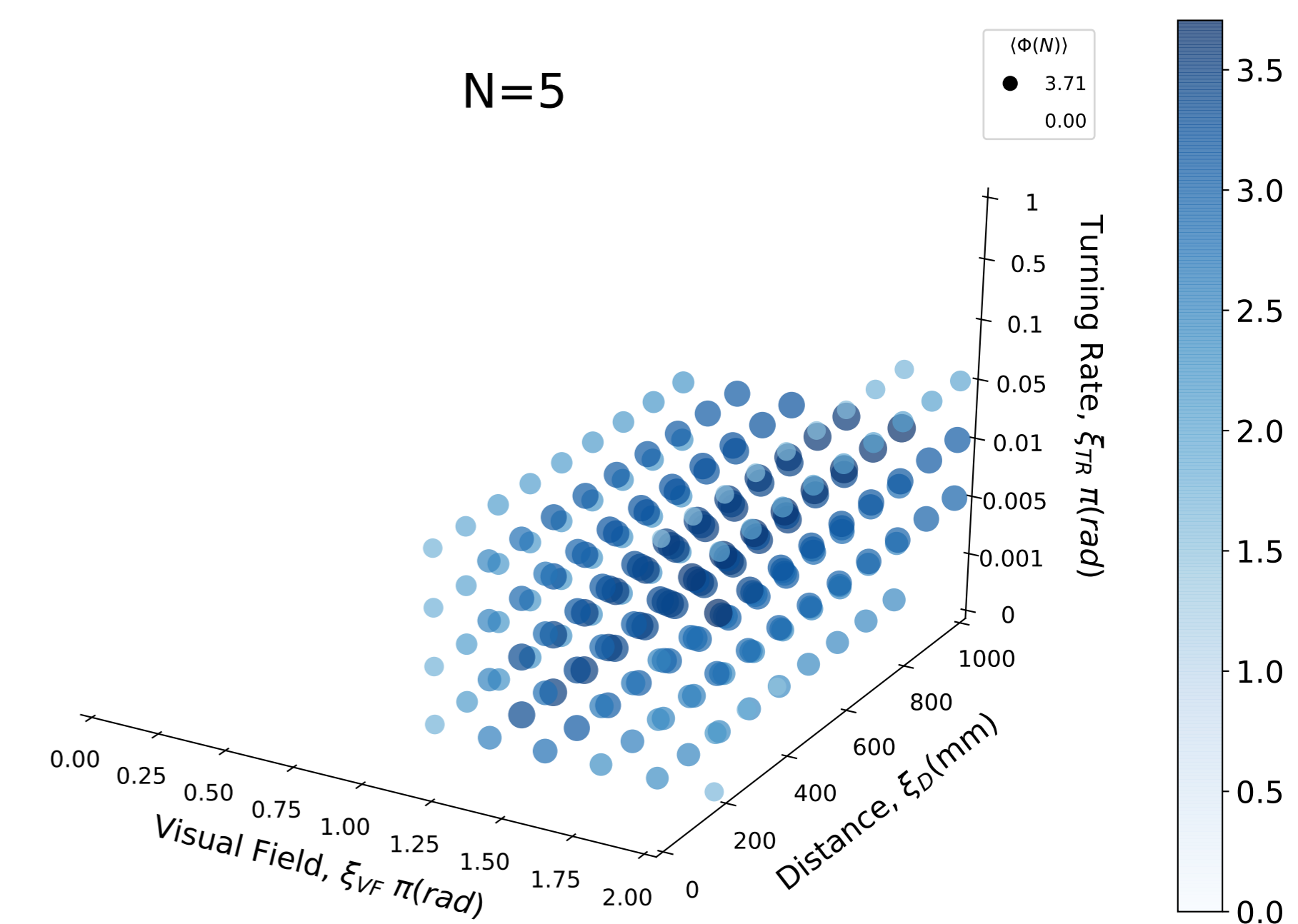

dt = 120/120 sec

N=2

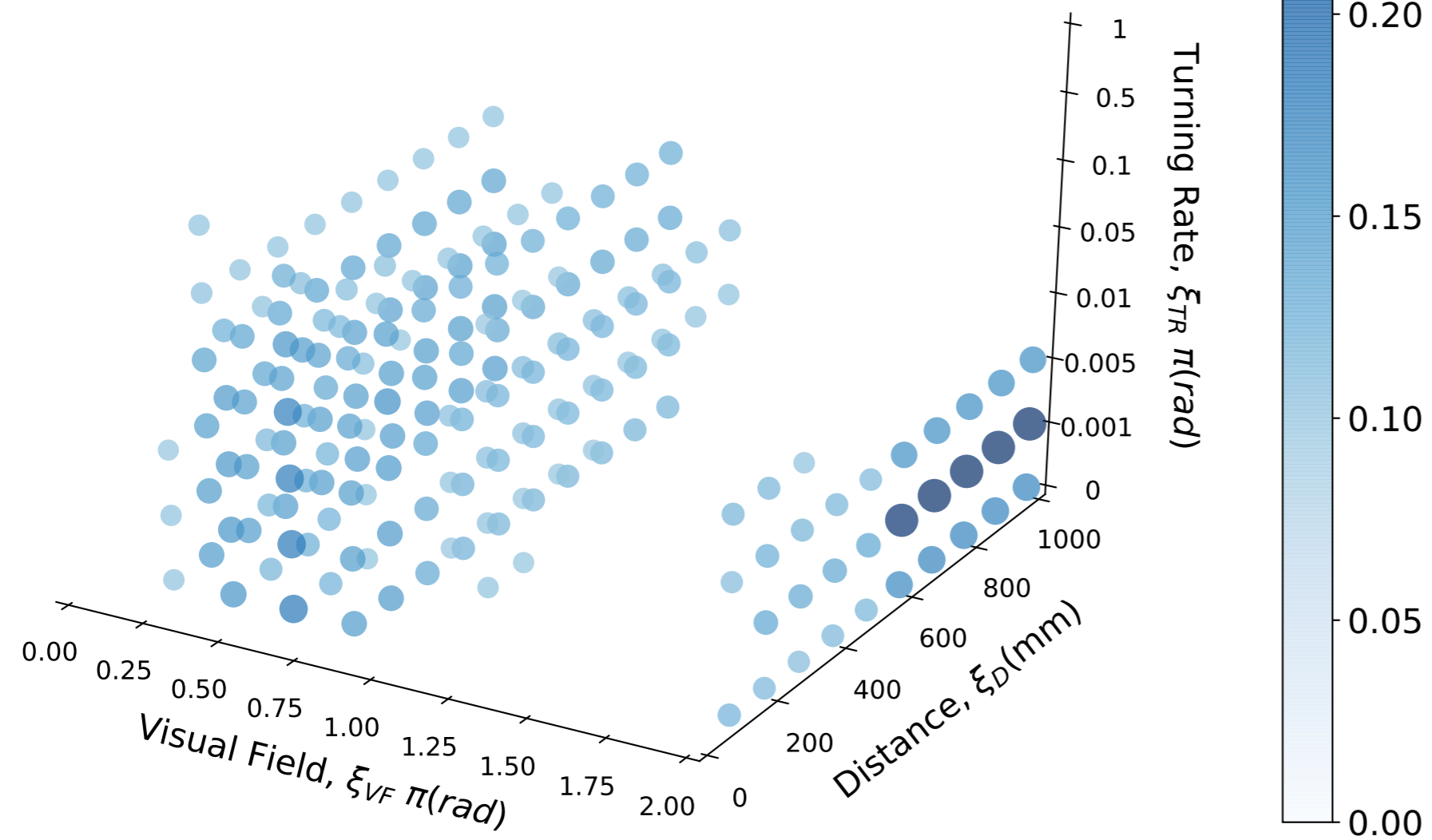

N=3

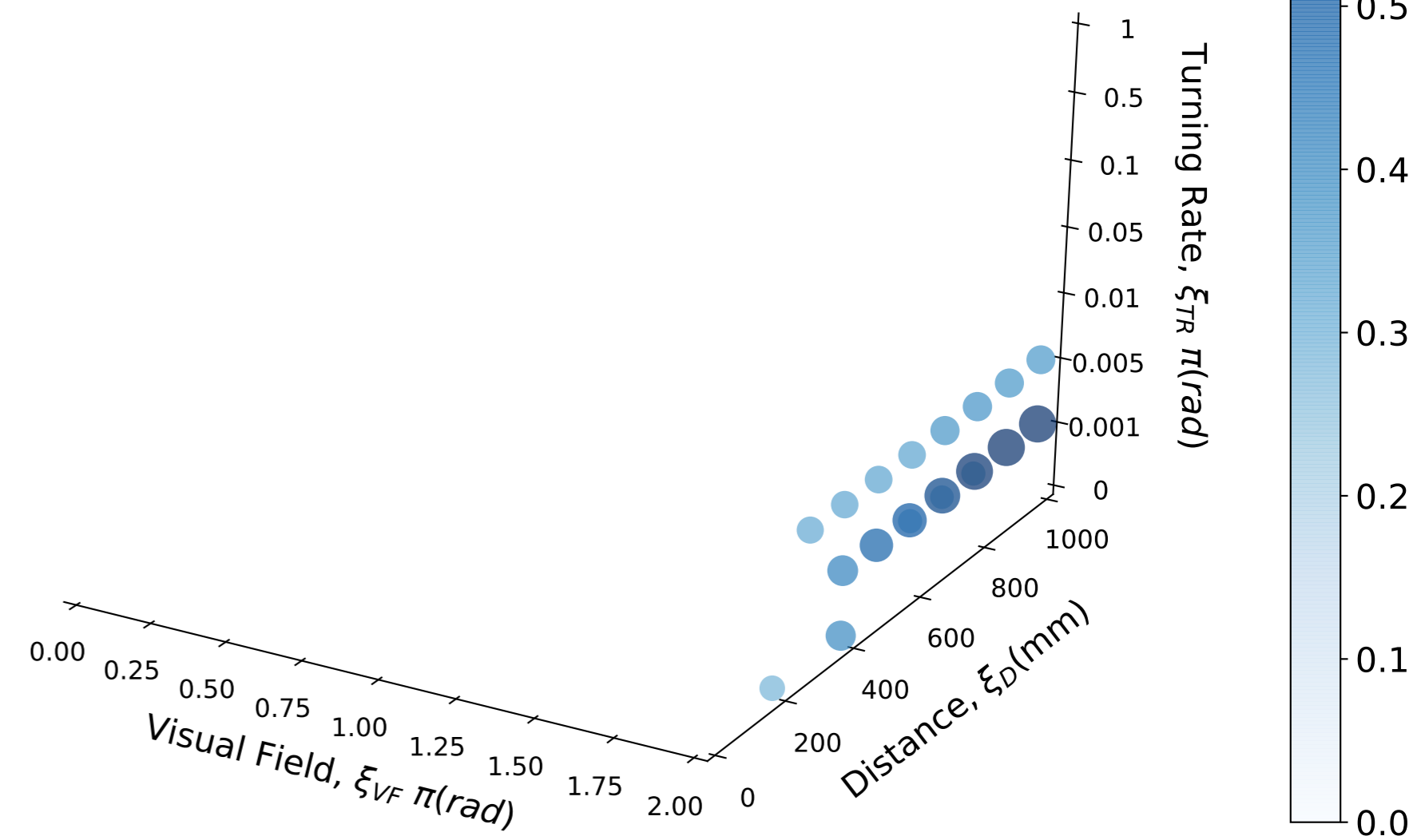

N=4

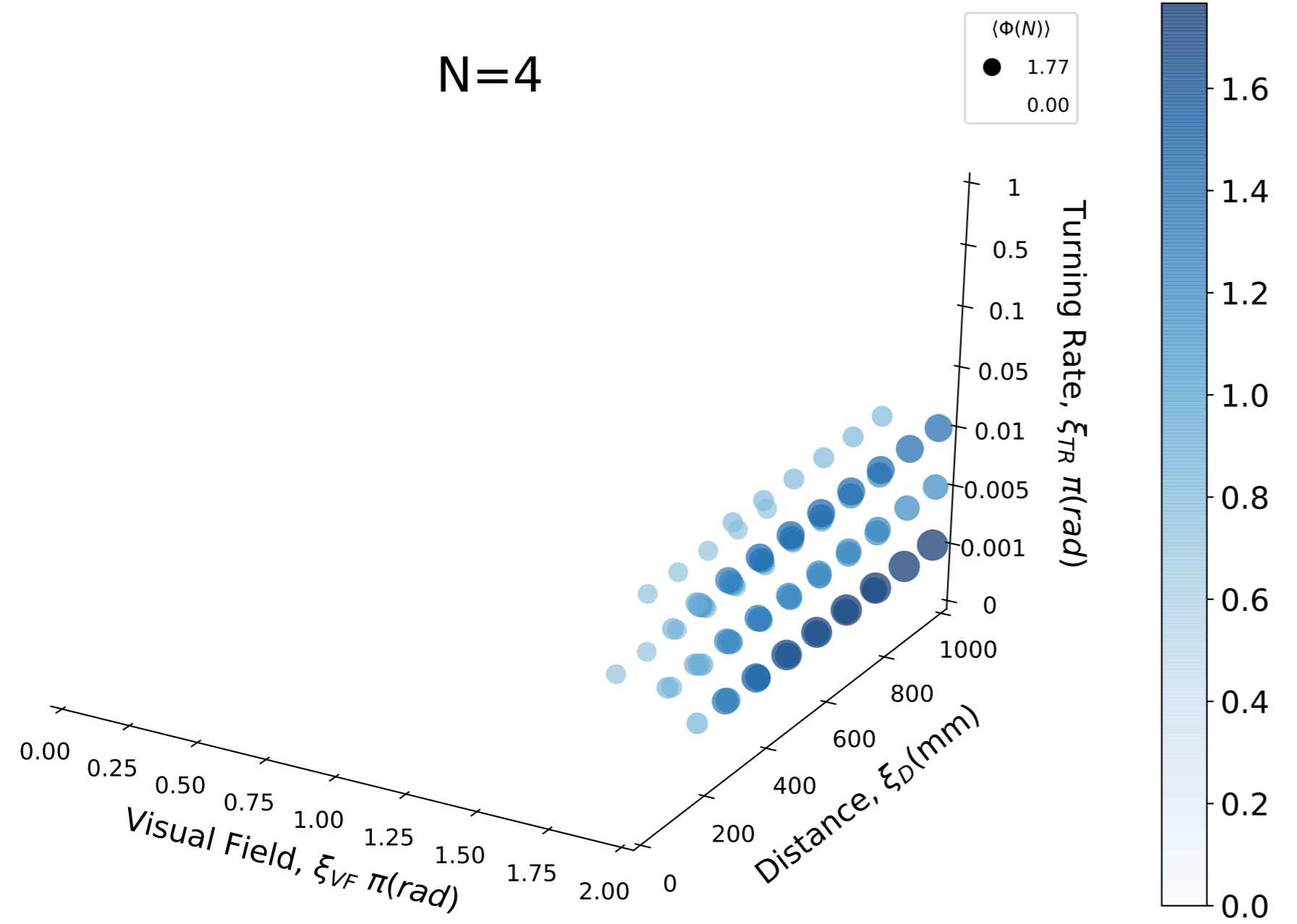

N=5

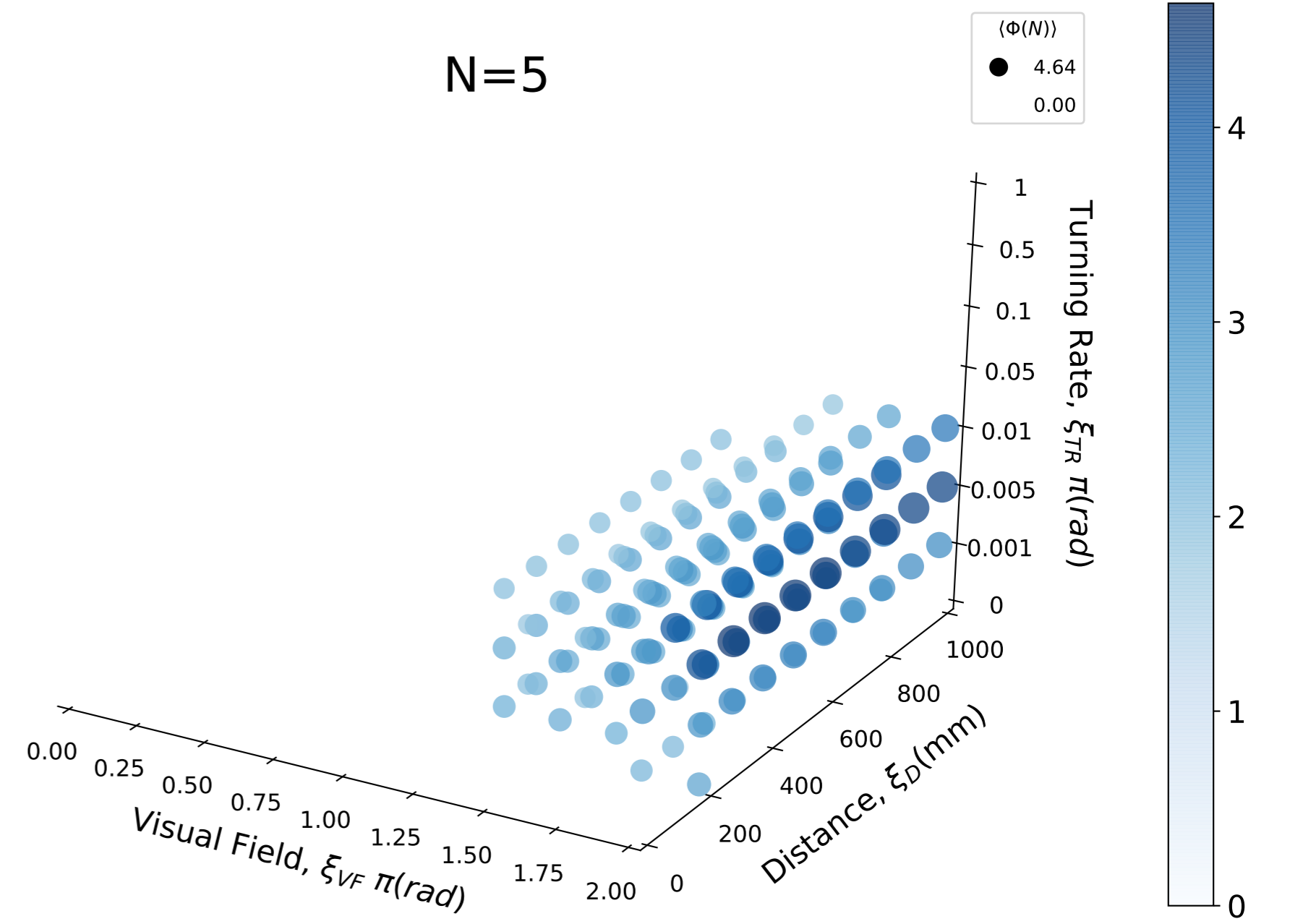

Supplement: Supplementary file 1 [file entropy-22-00726-s001.zip › Supporting Information/Figure_S1.pdf]

dt=5/120 s

**N = 2**

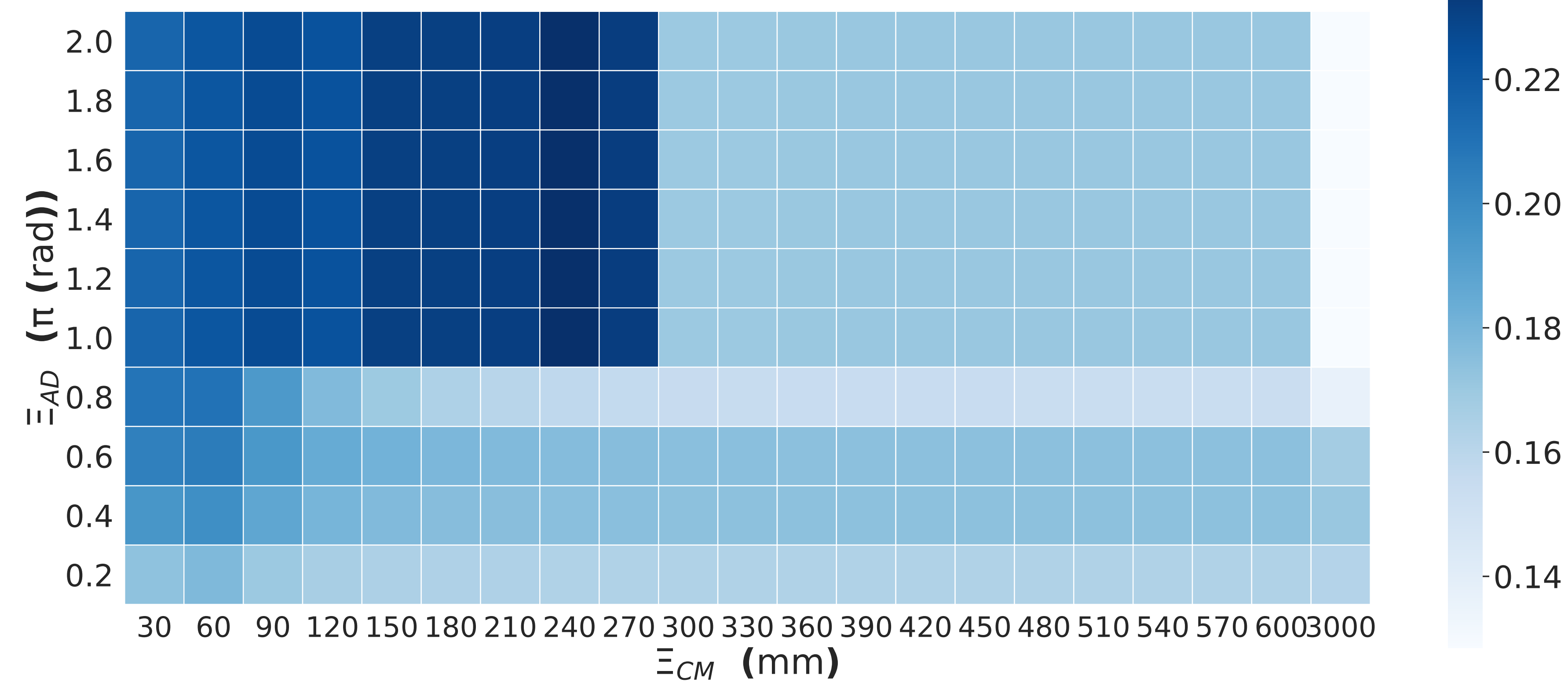

**N = 3**

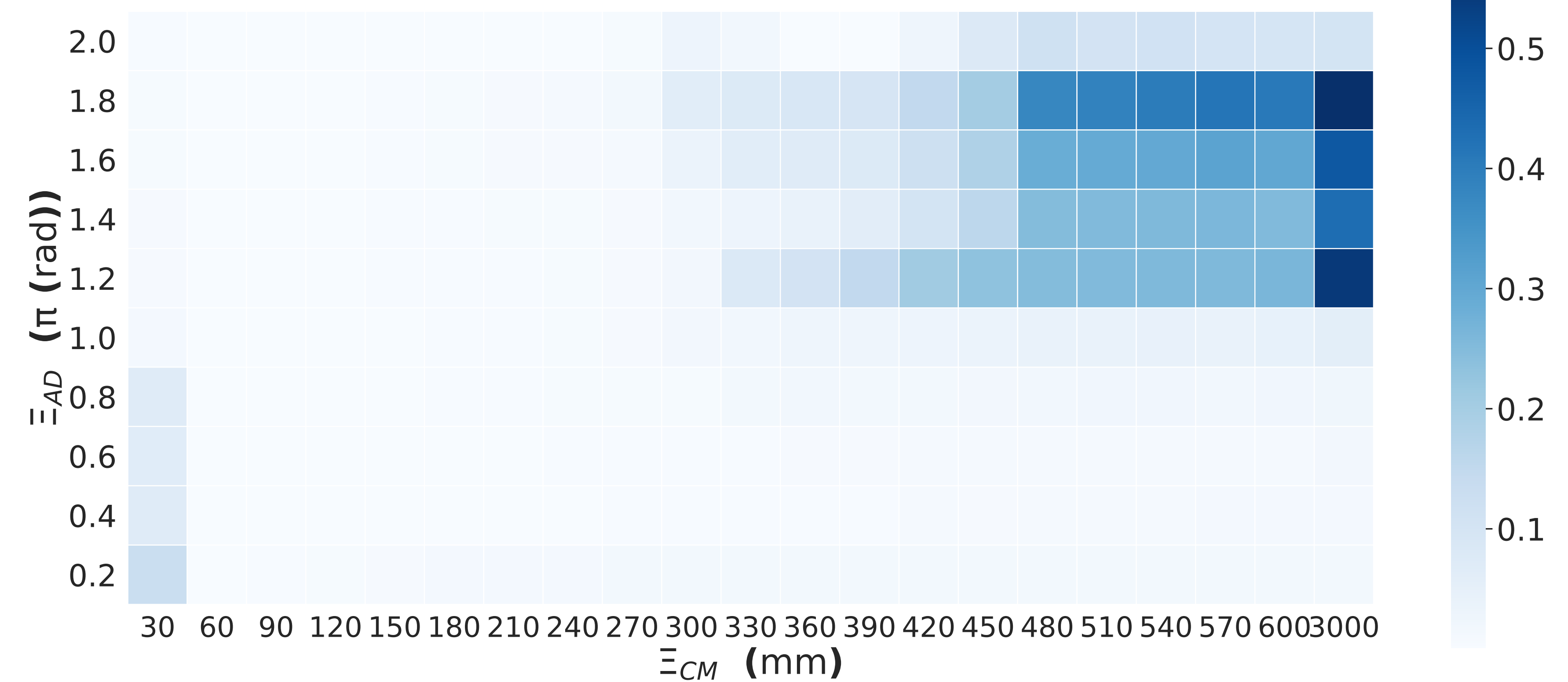

**N = 4**

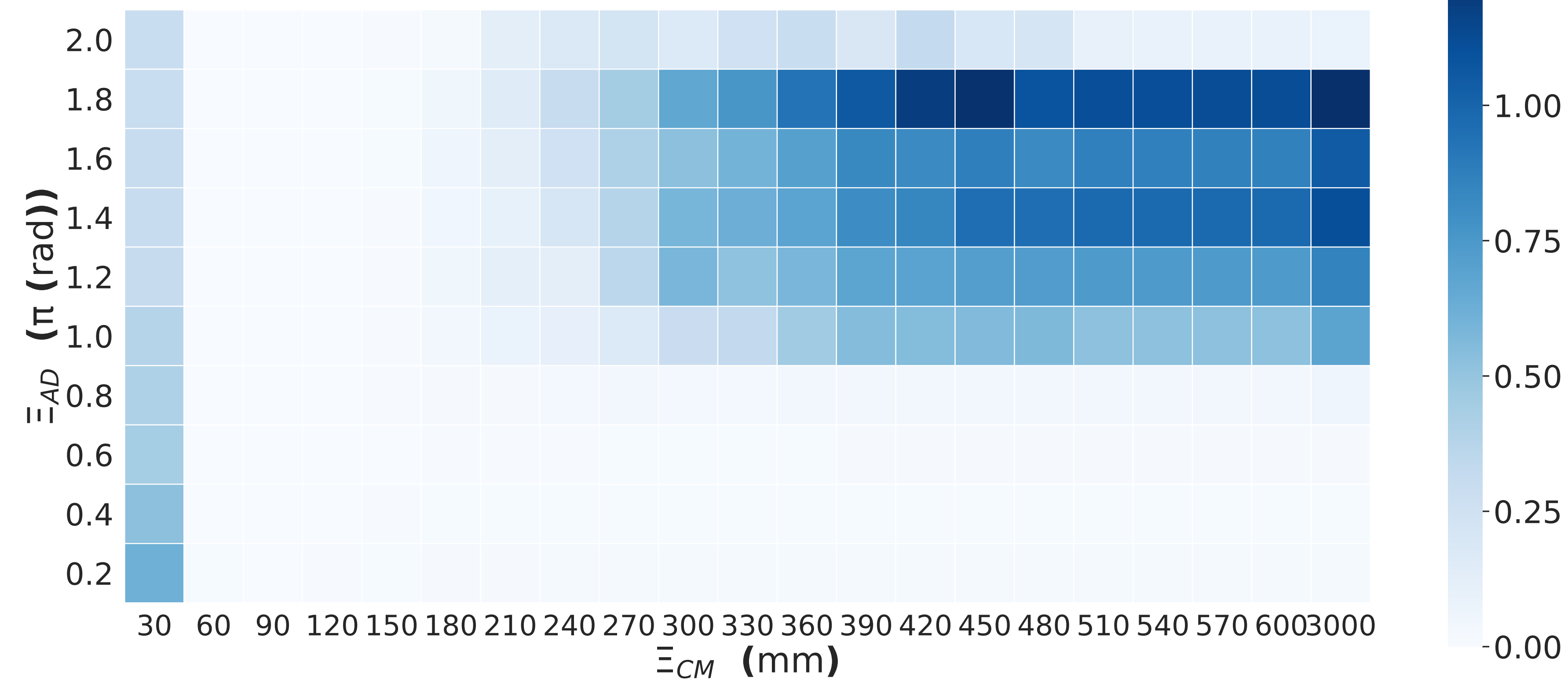

**N = 5**

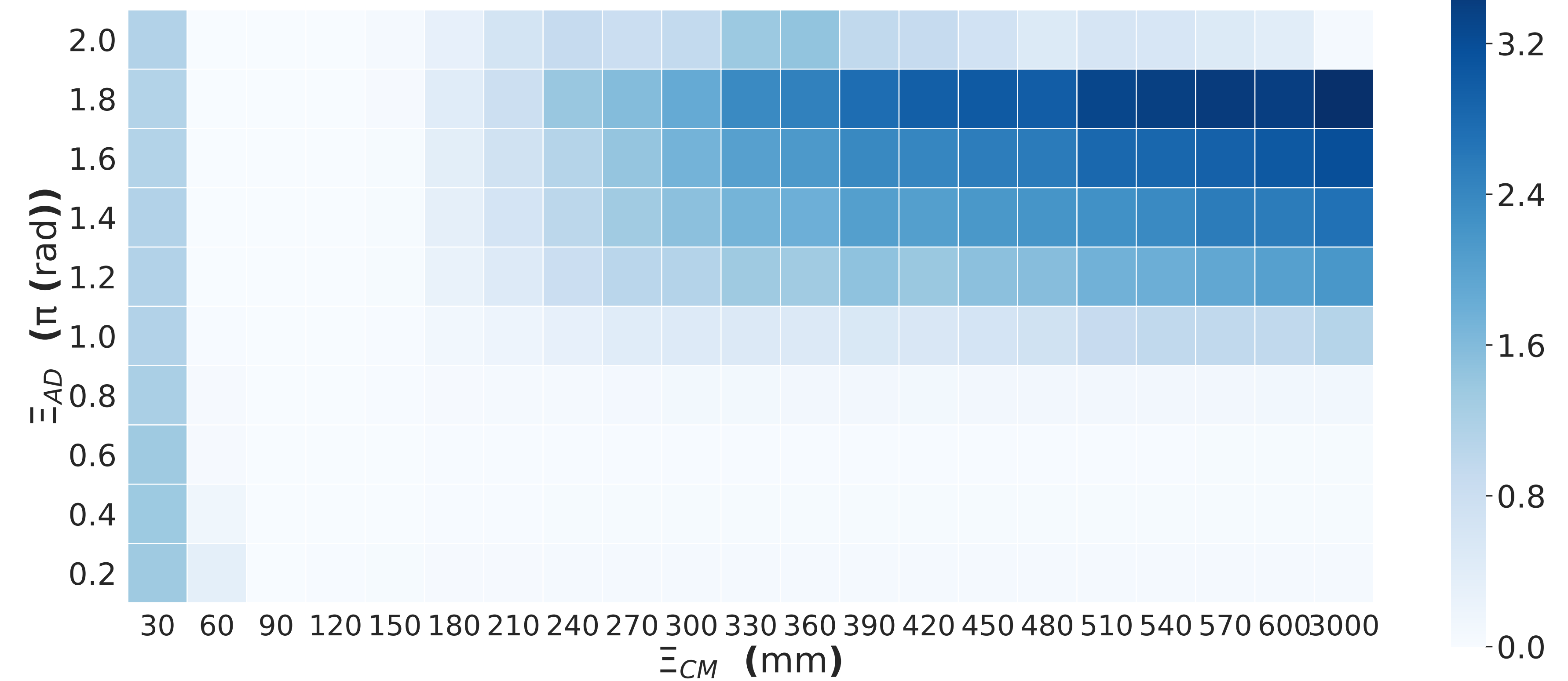

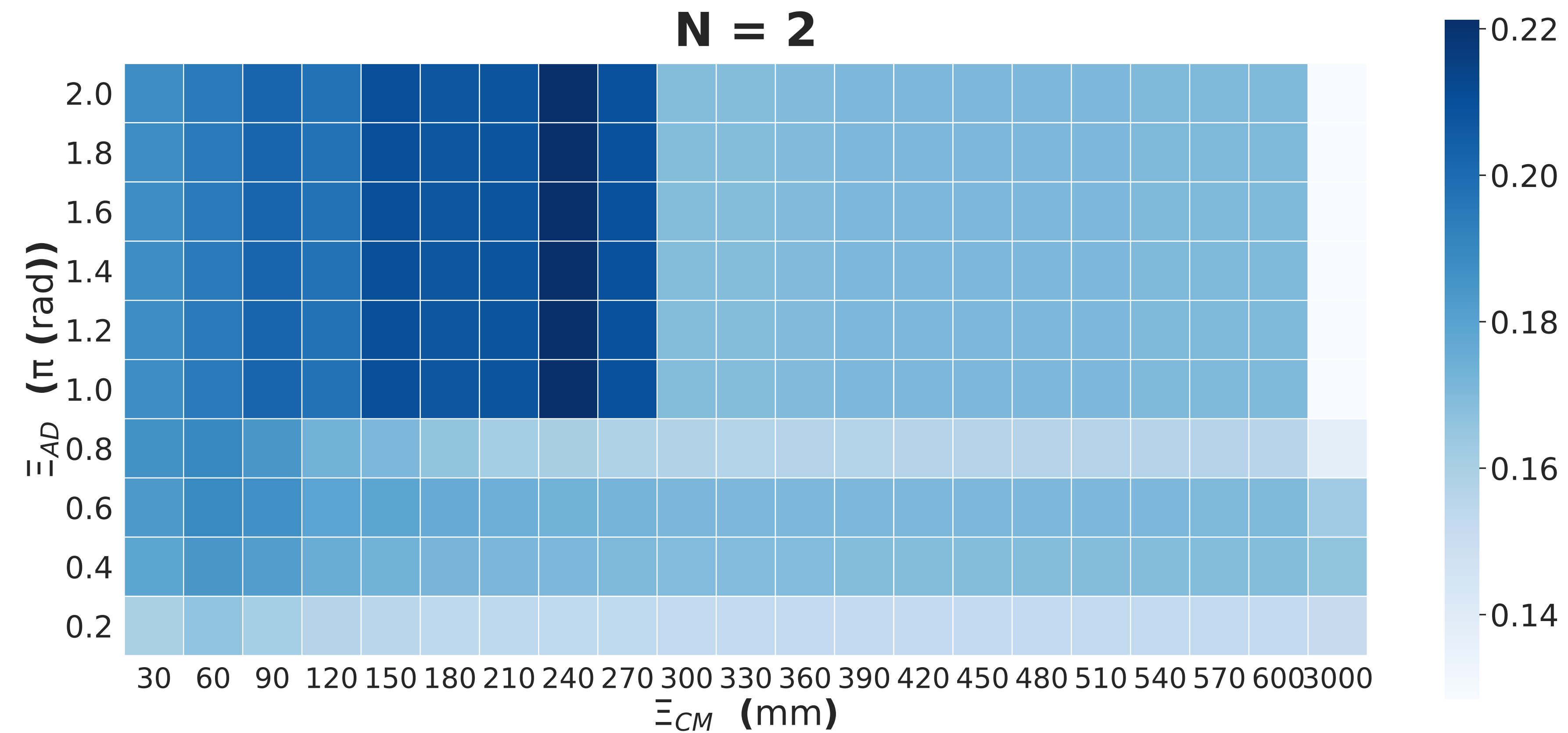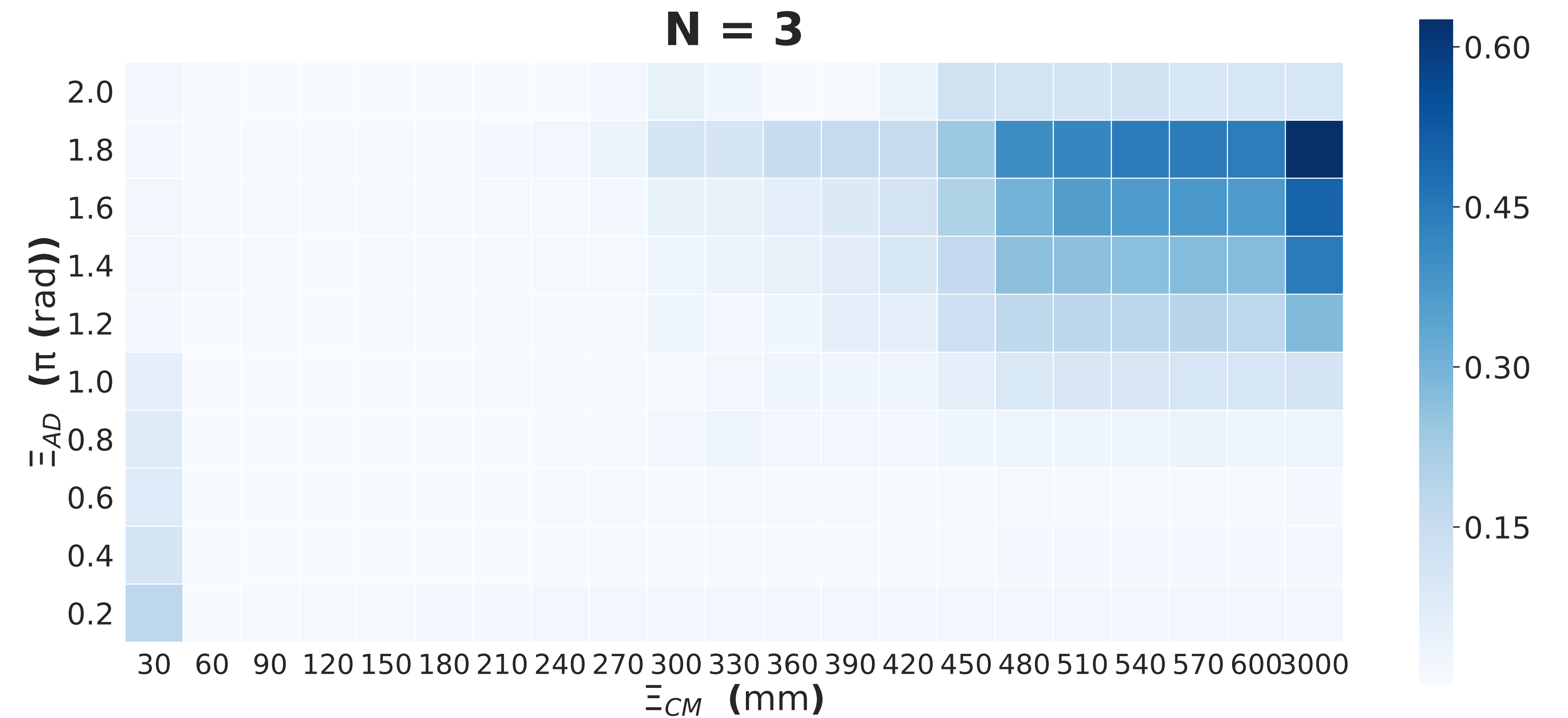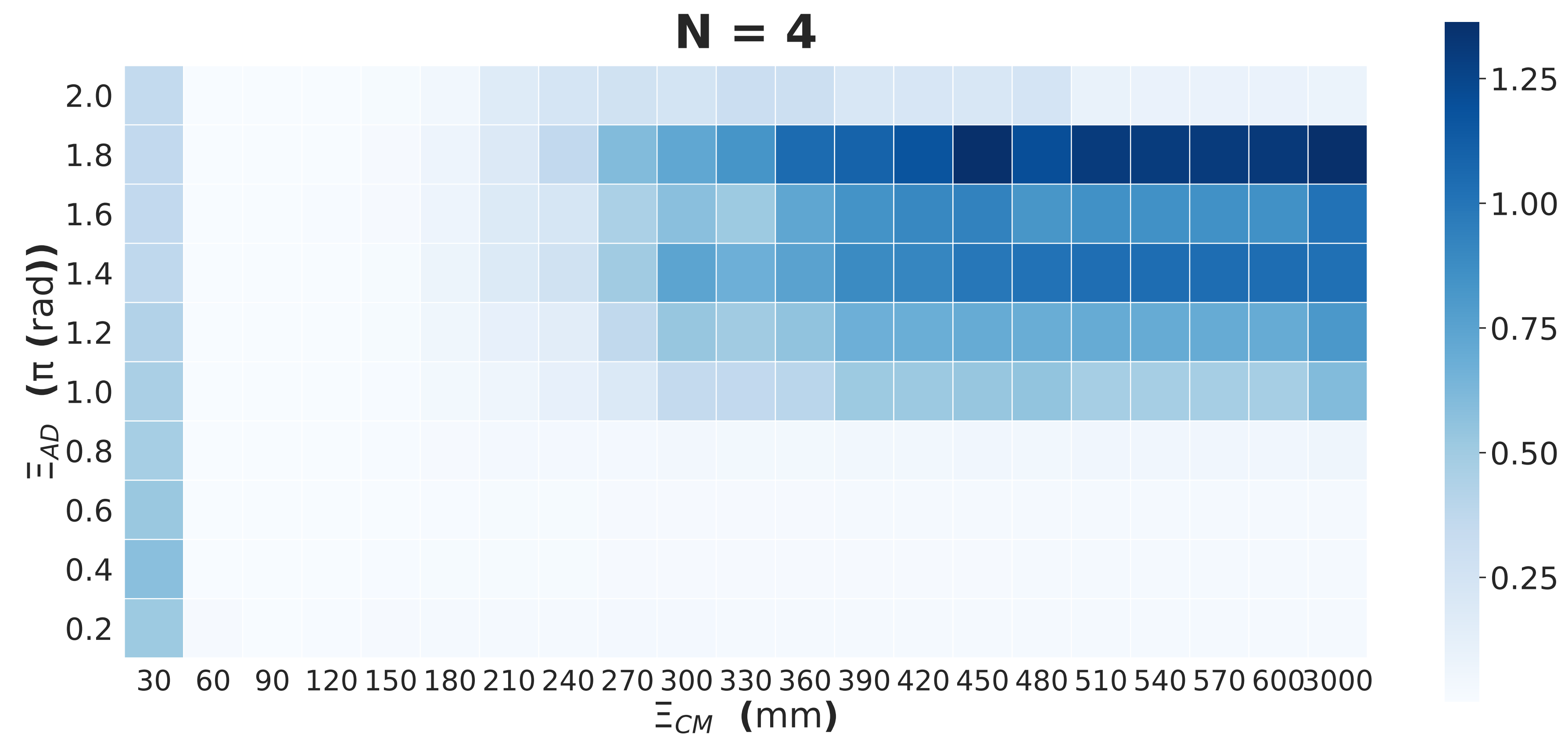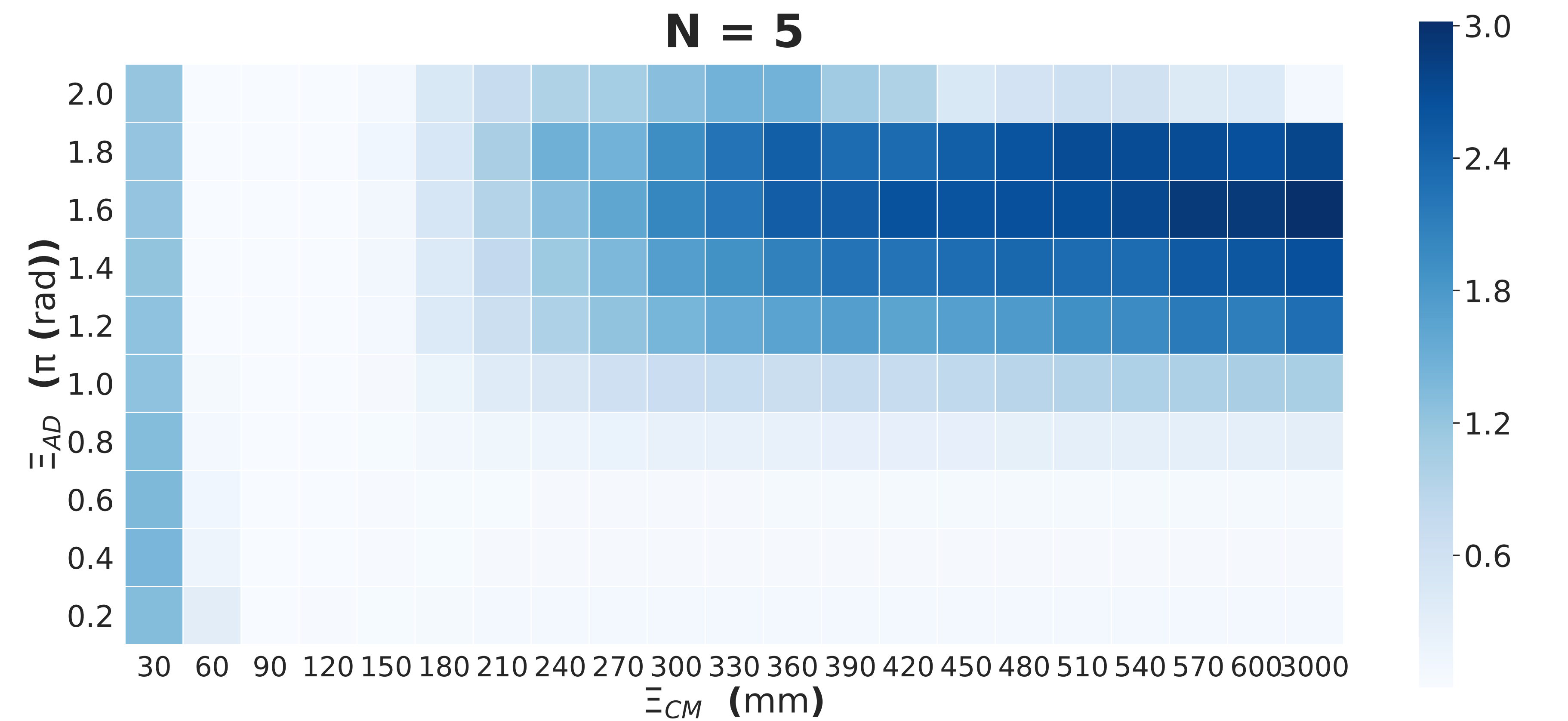

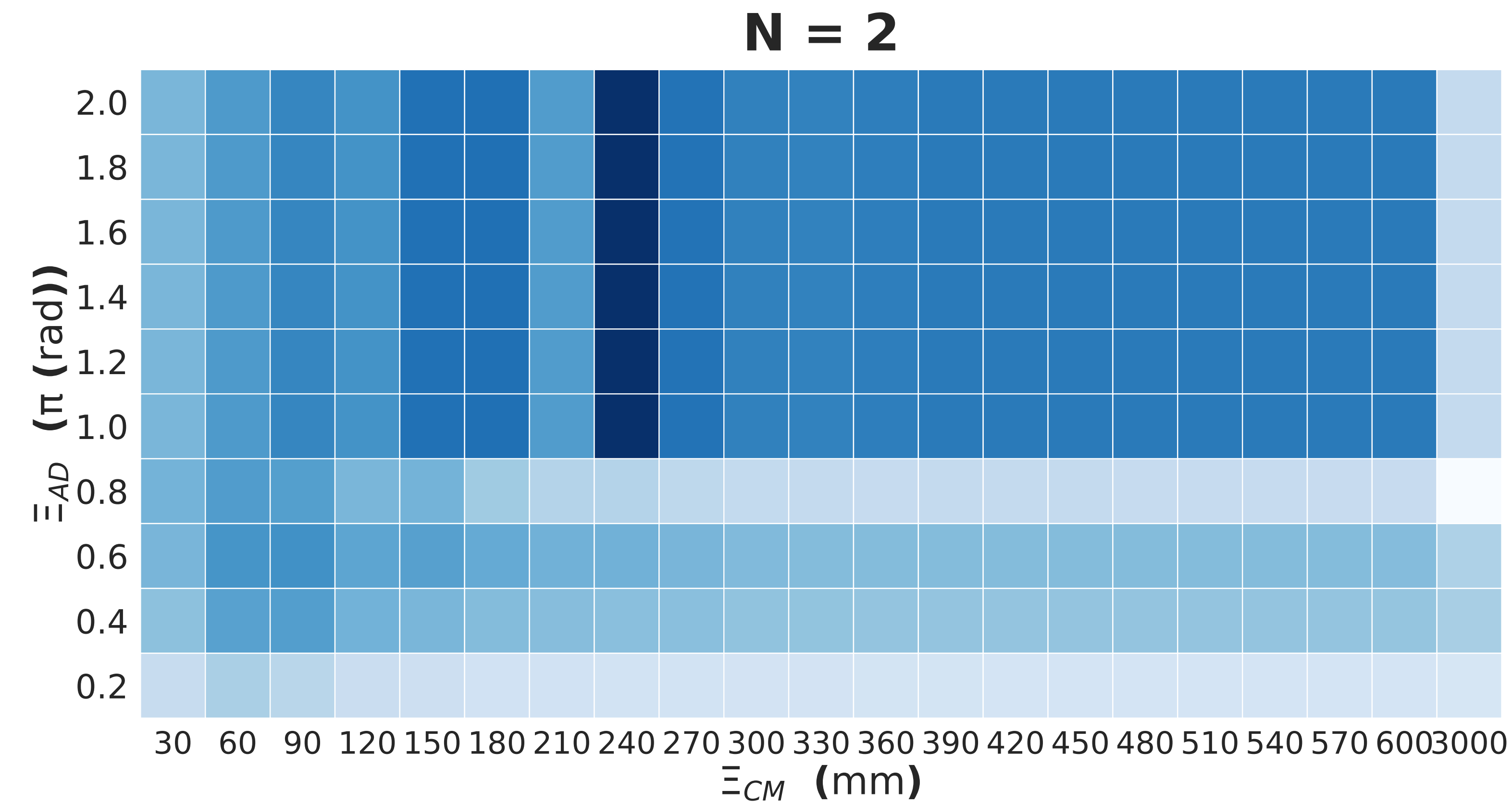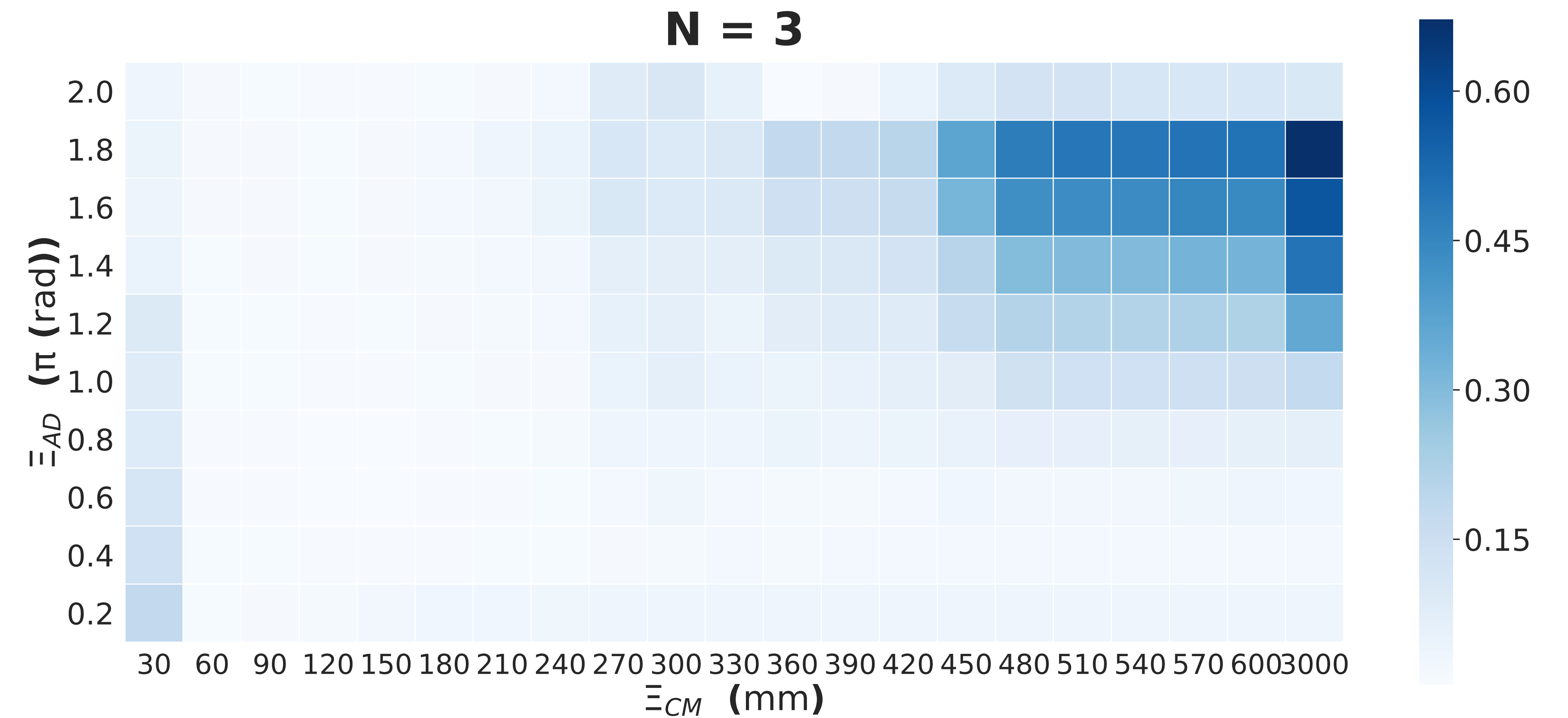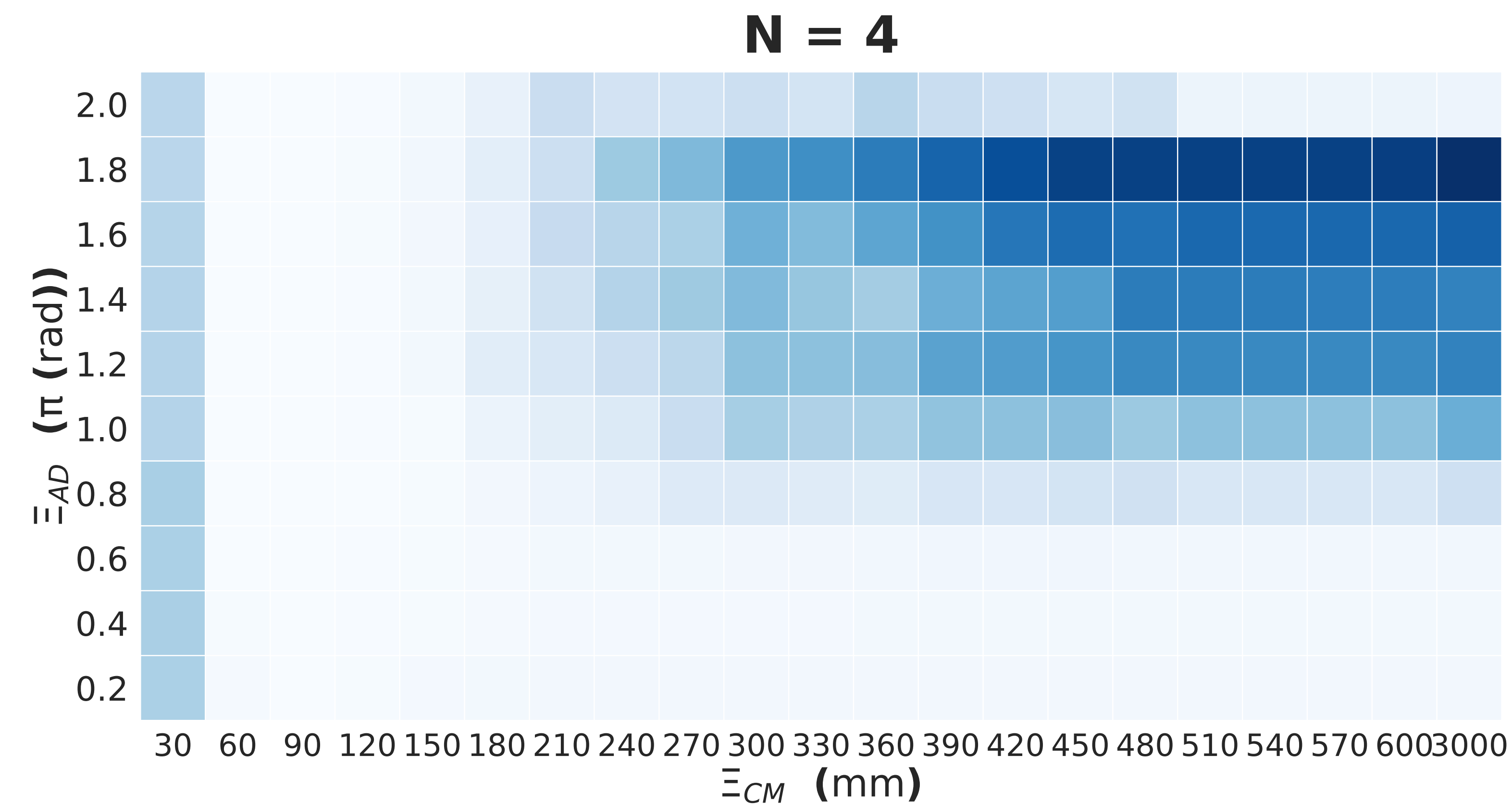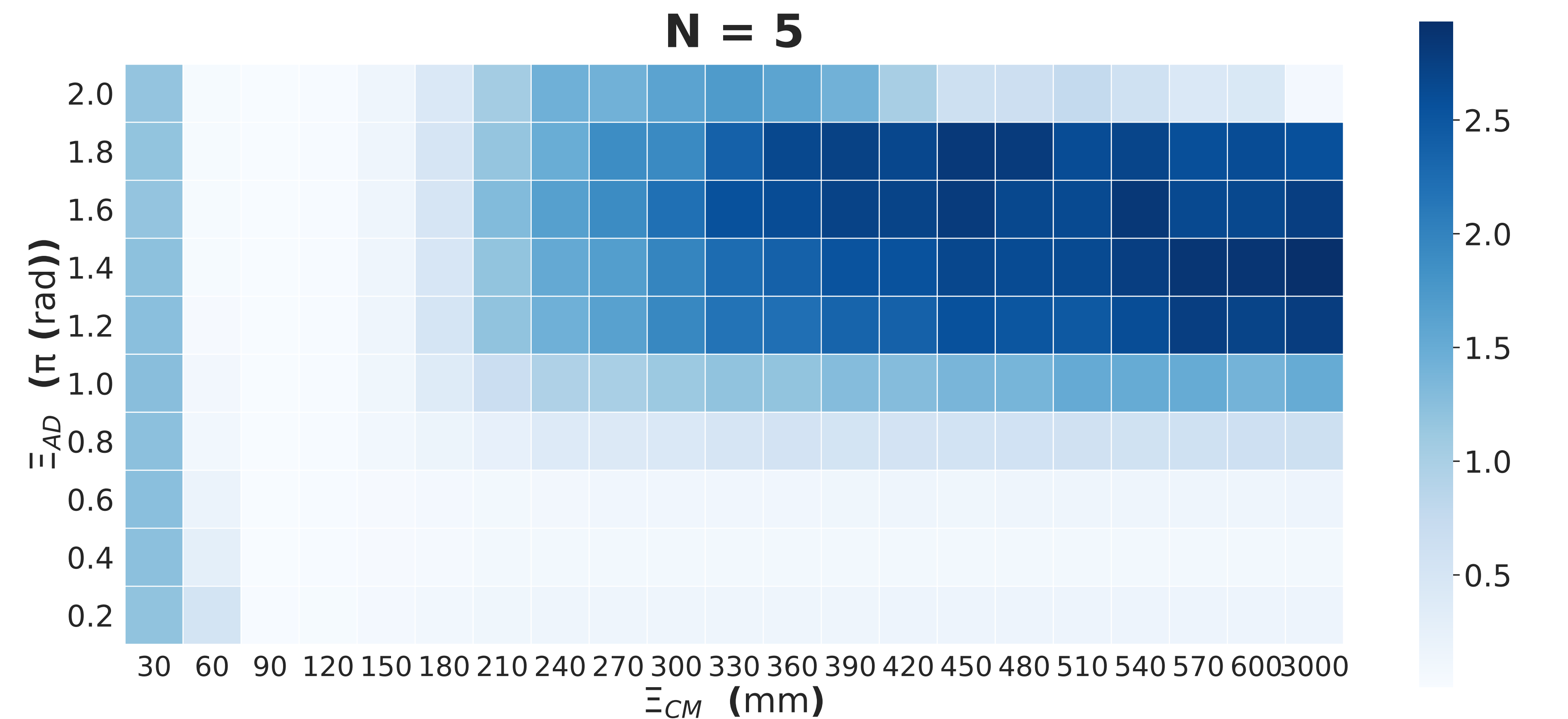

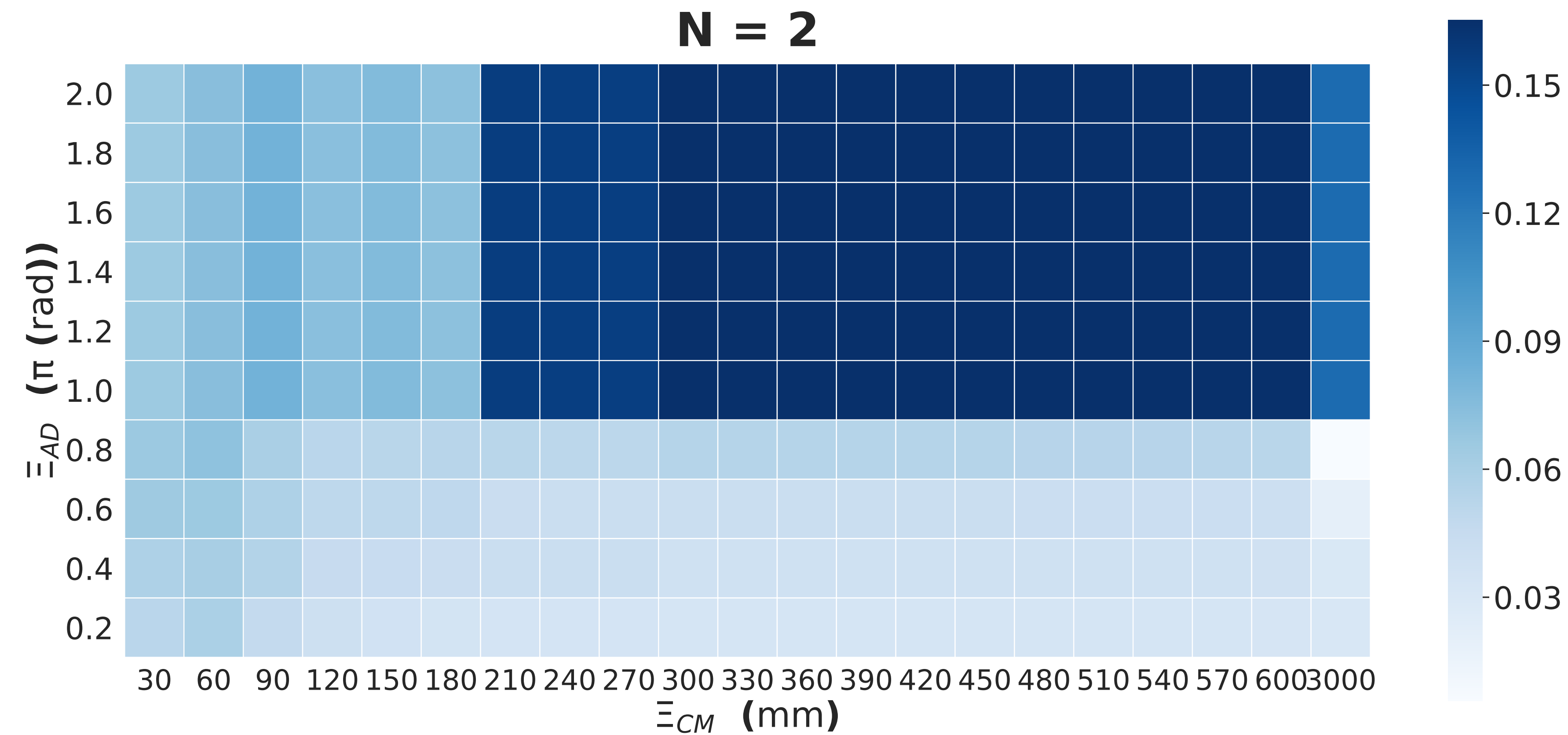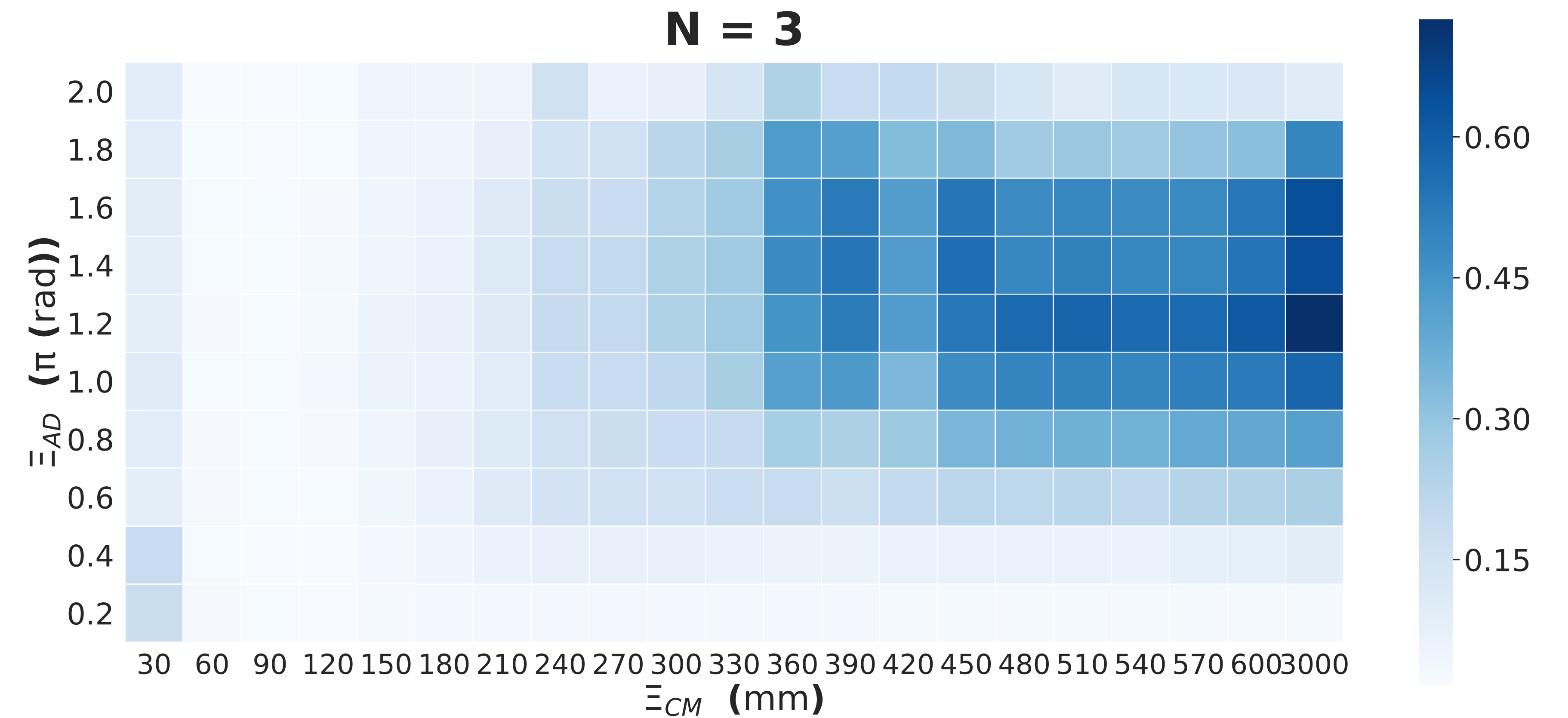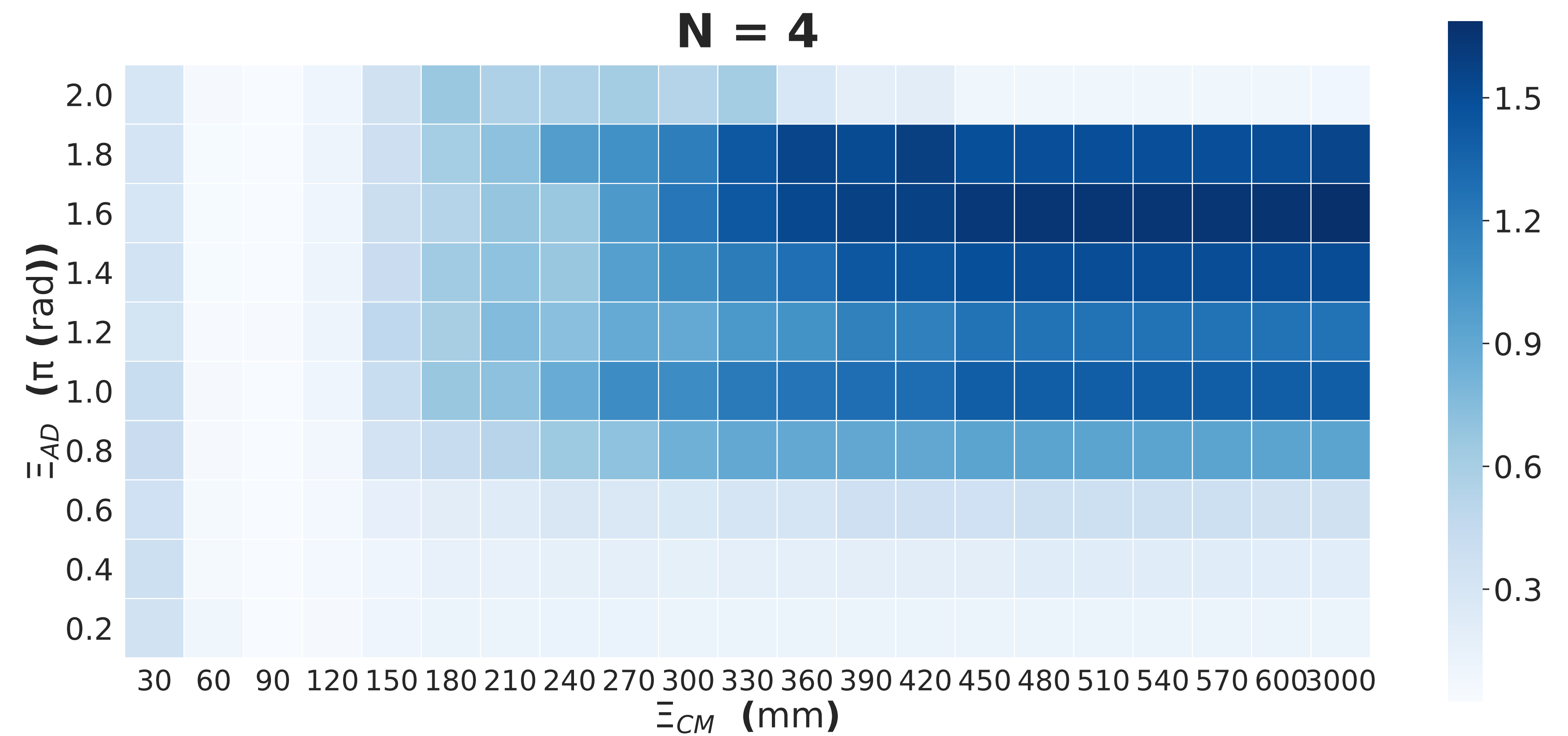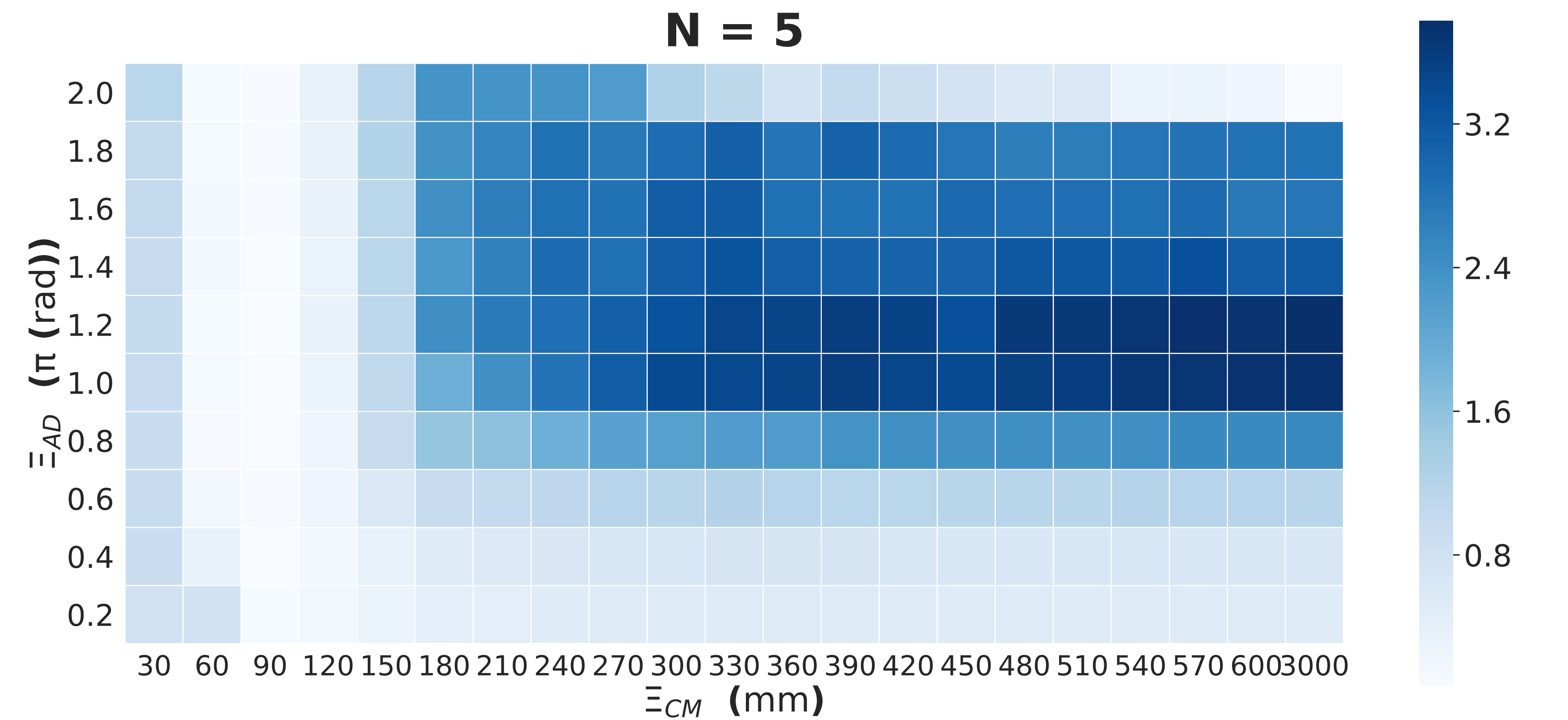

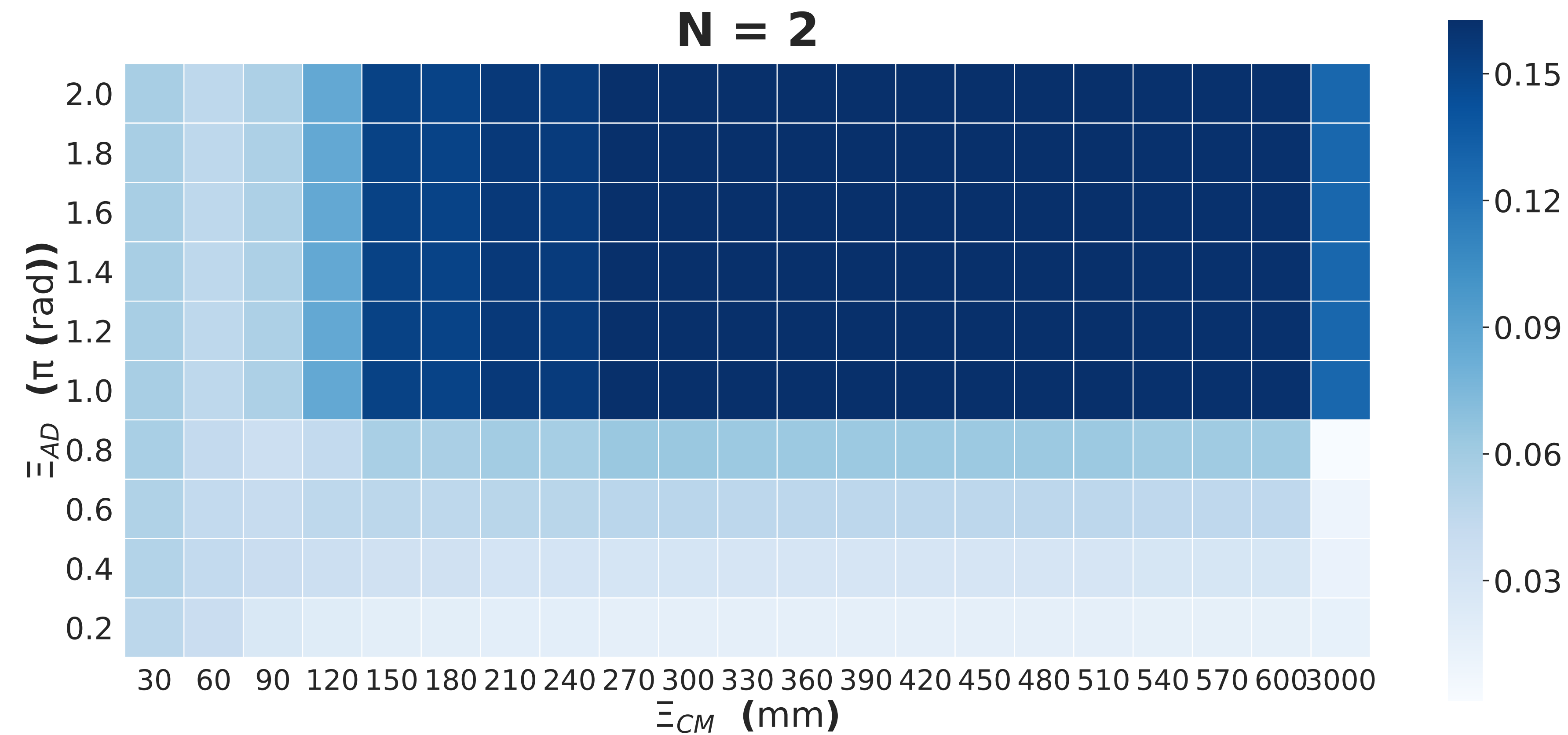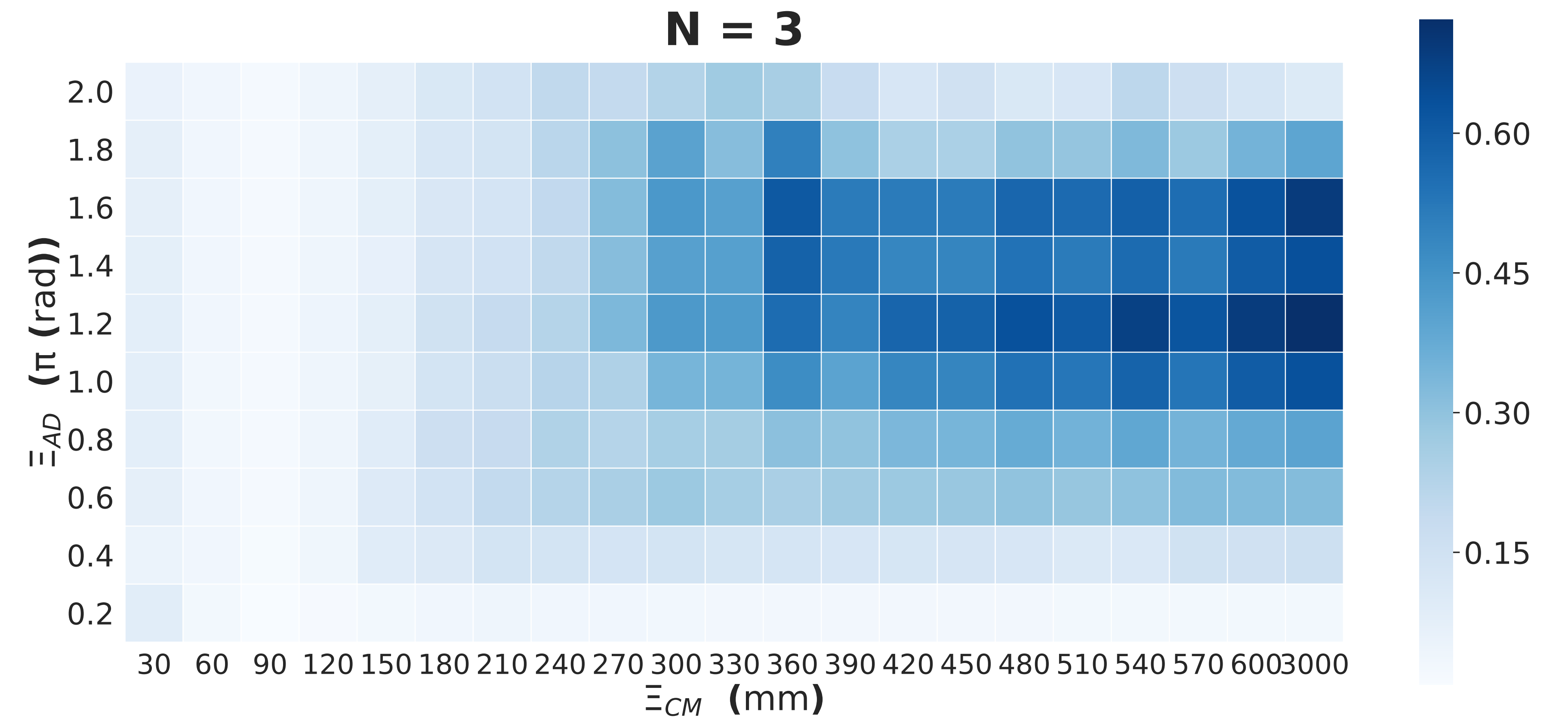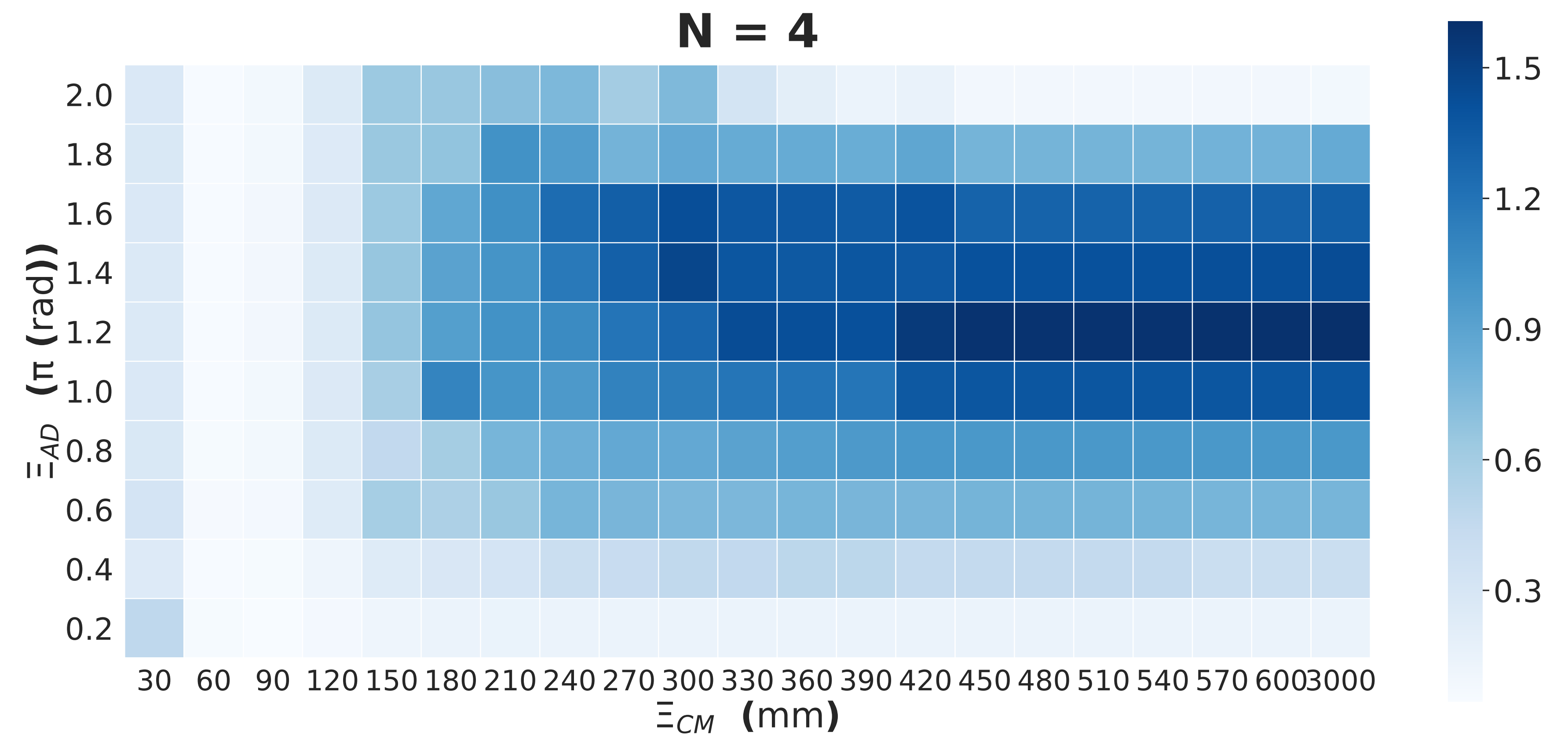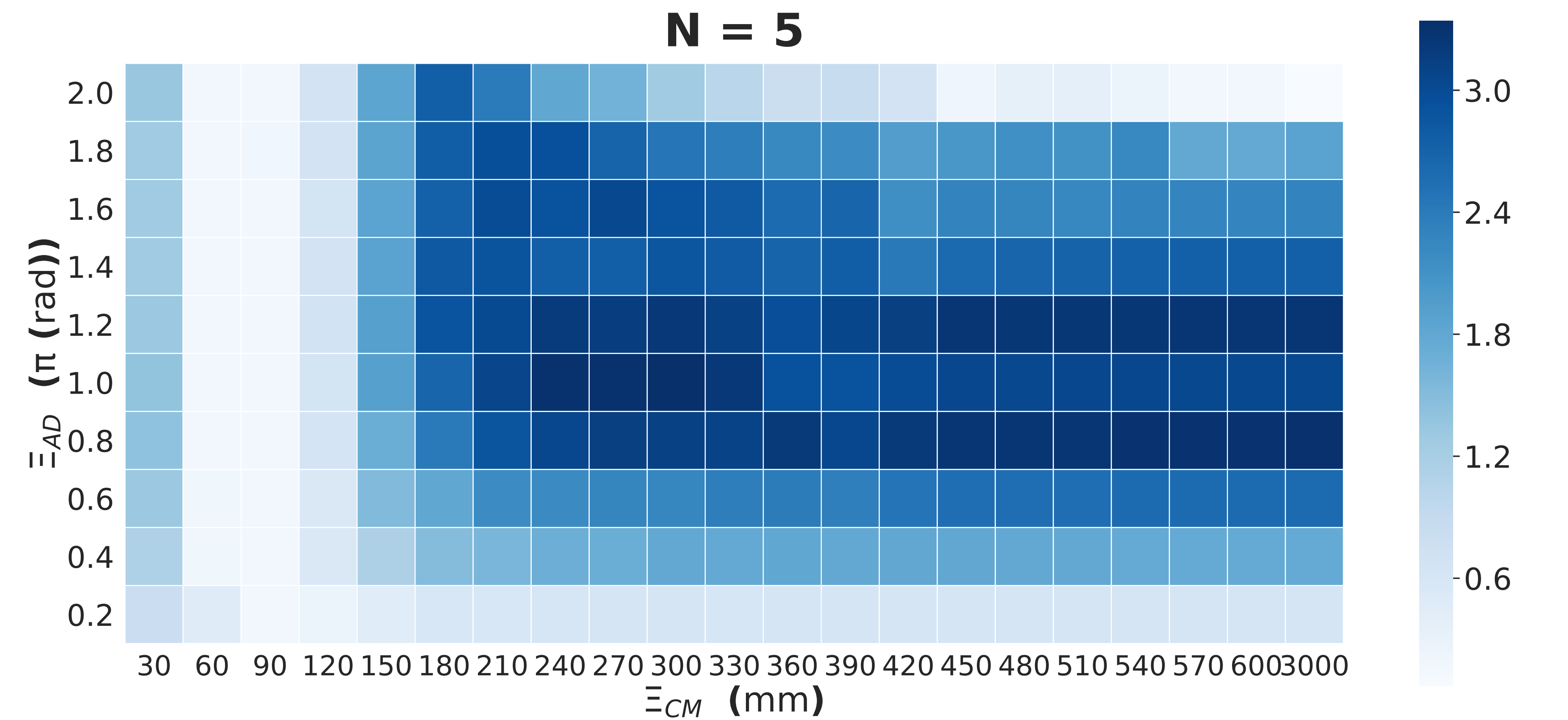

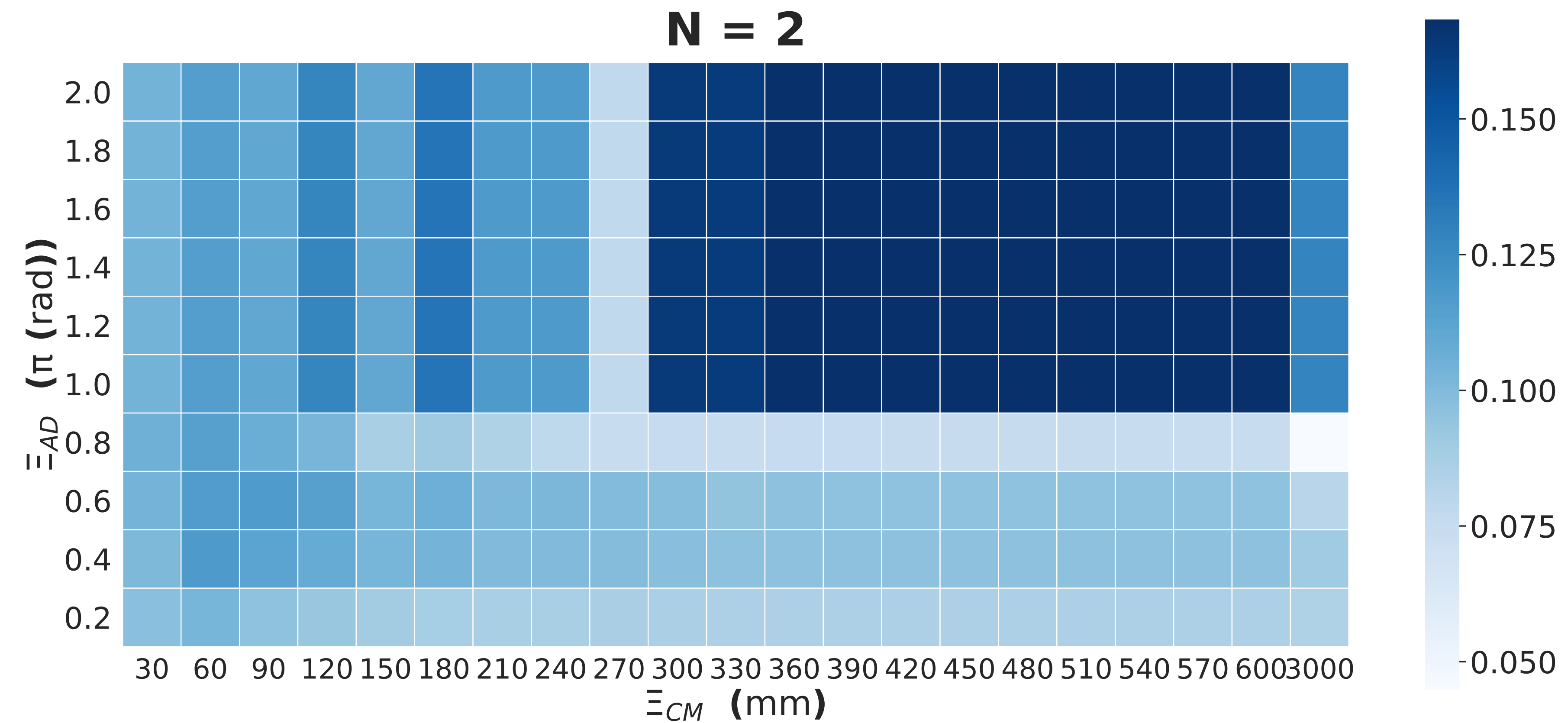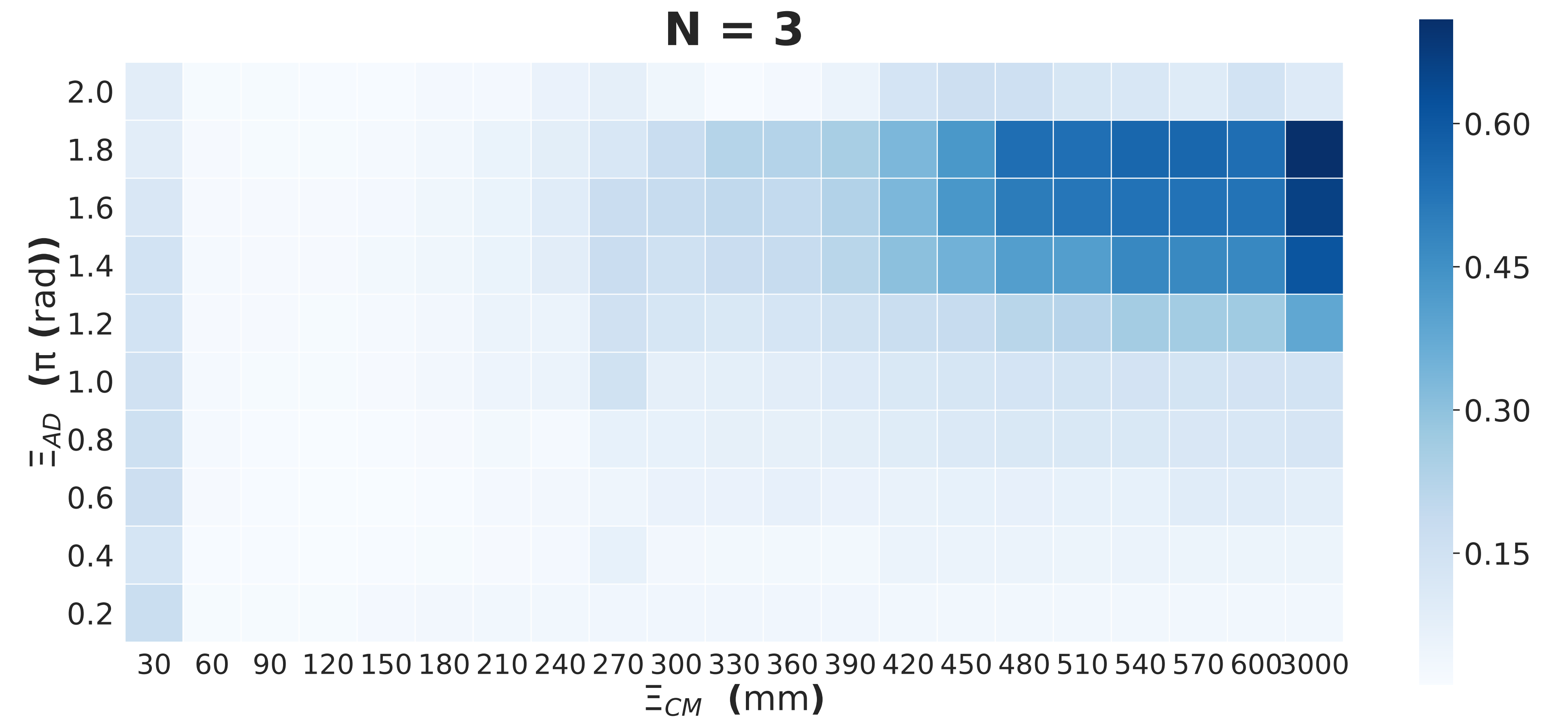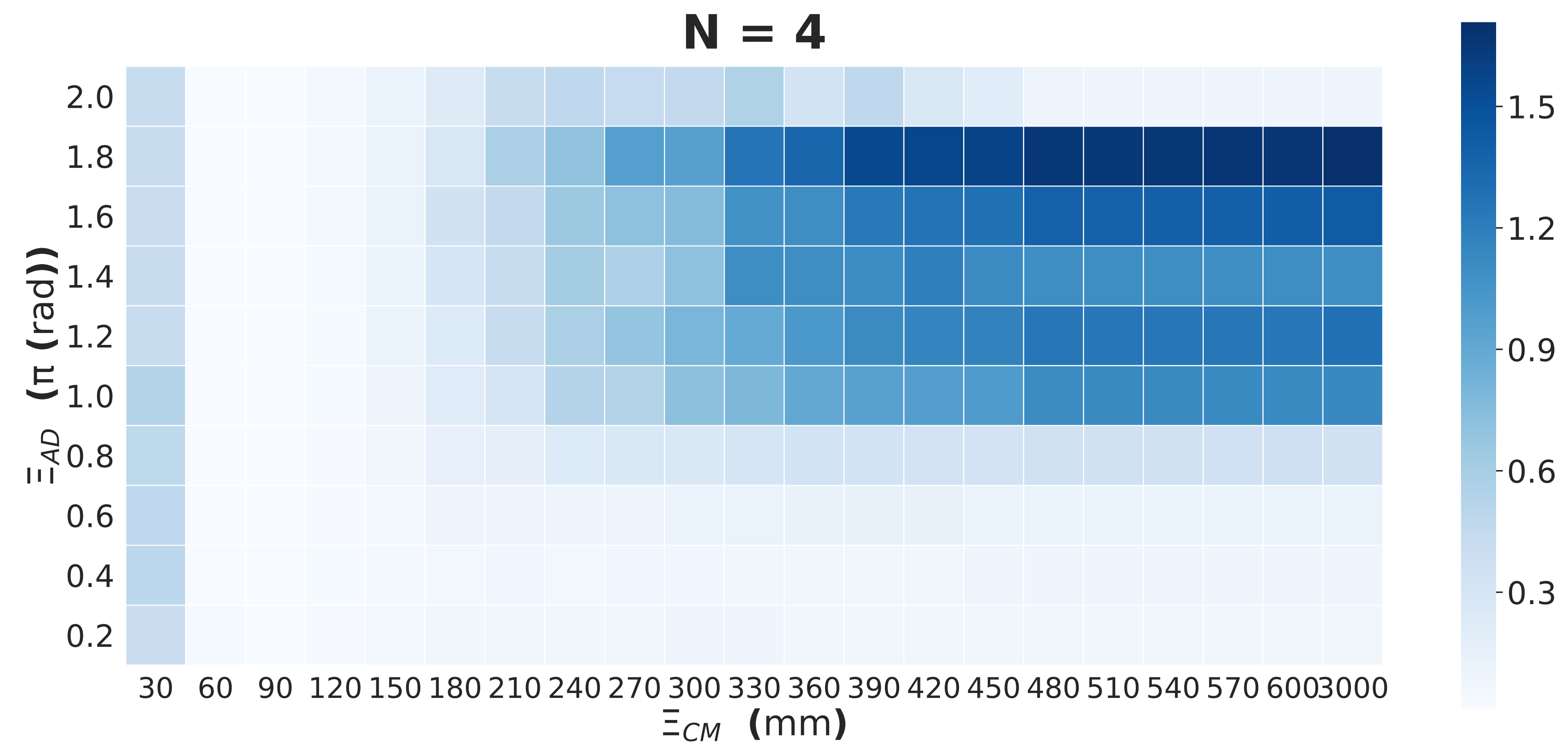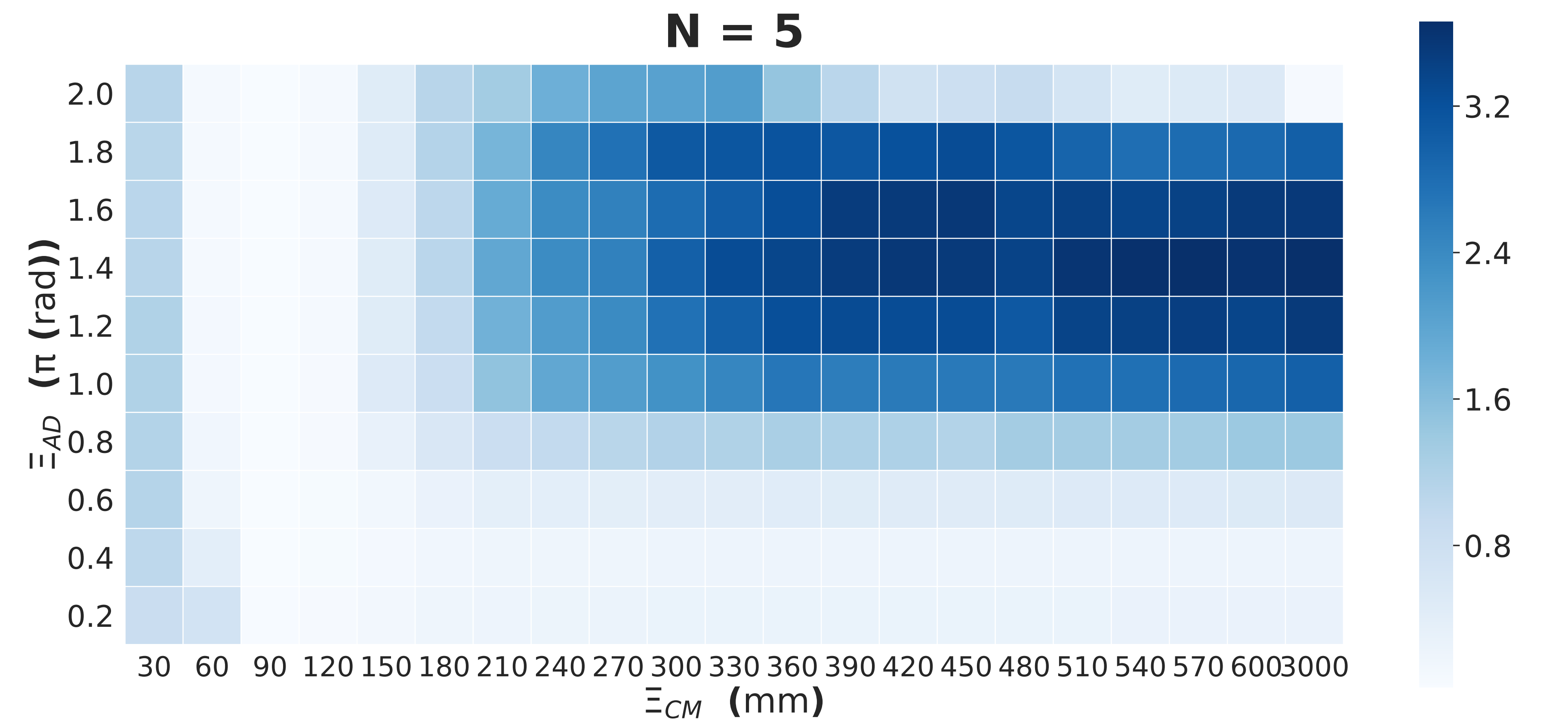

Supplement: Supplementary file 1 [file entropy-22-00726-s001.zip › Supporting Information/Figure_S5.pdf]

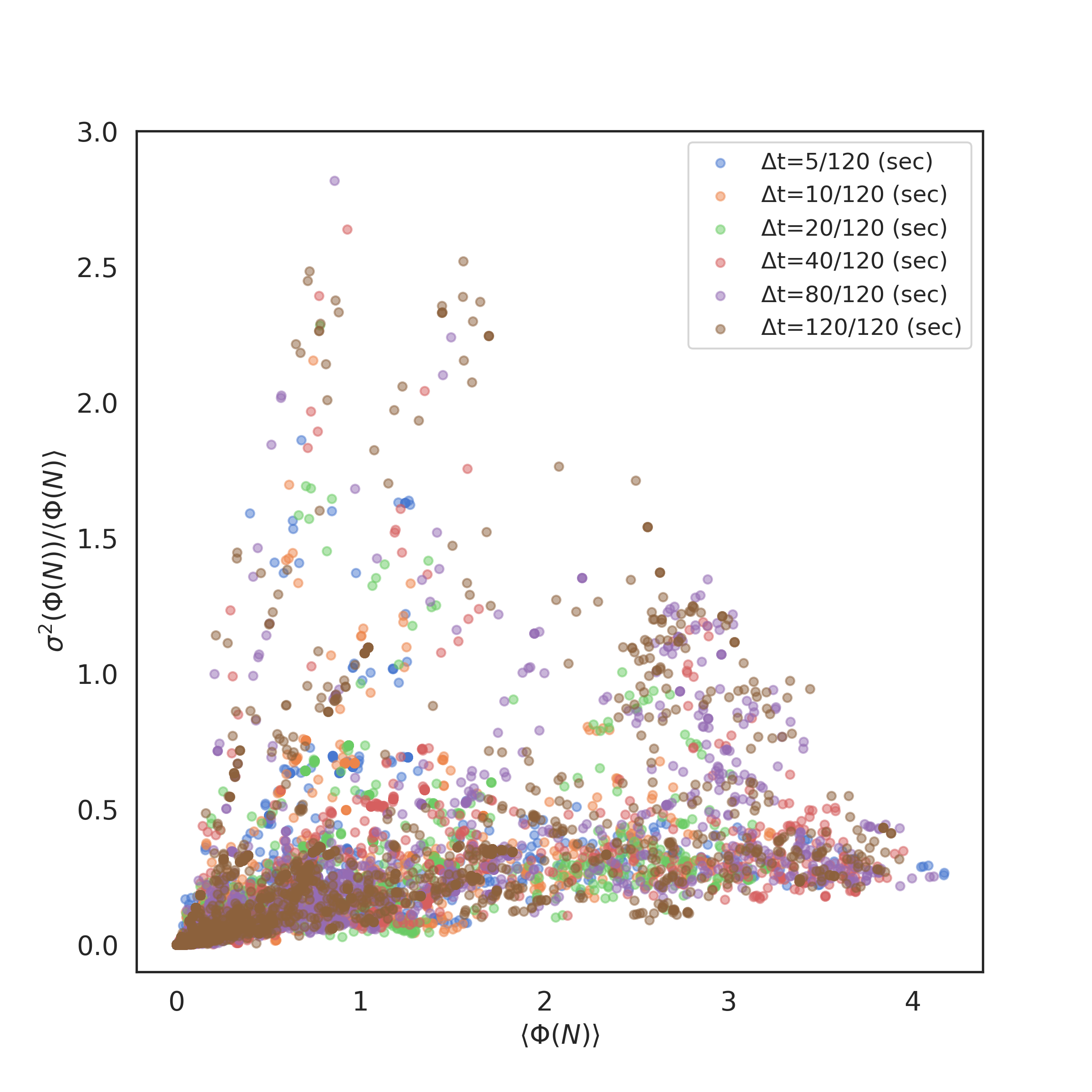

Supplement: Supplementary file 1 [file entropy-22-00726-s001.zip › Supporting Information/FIgure_S6.png]

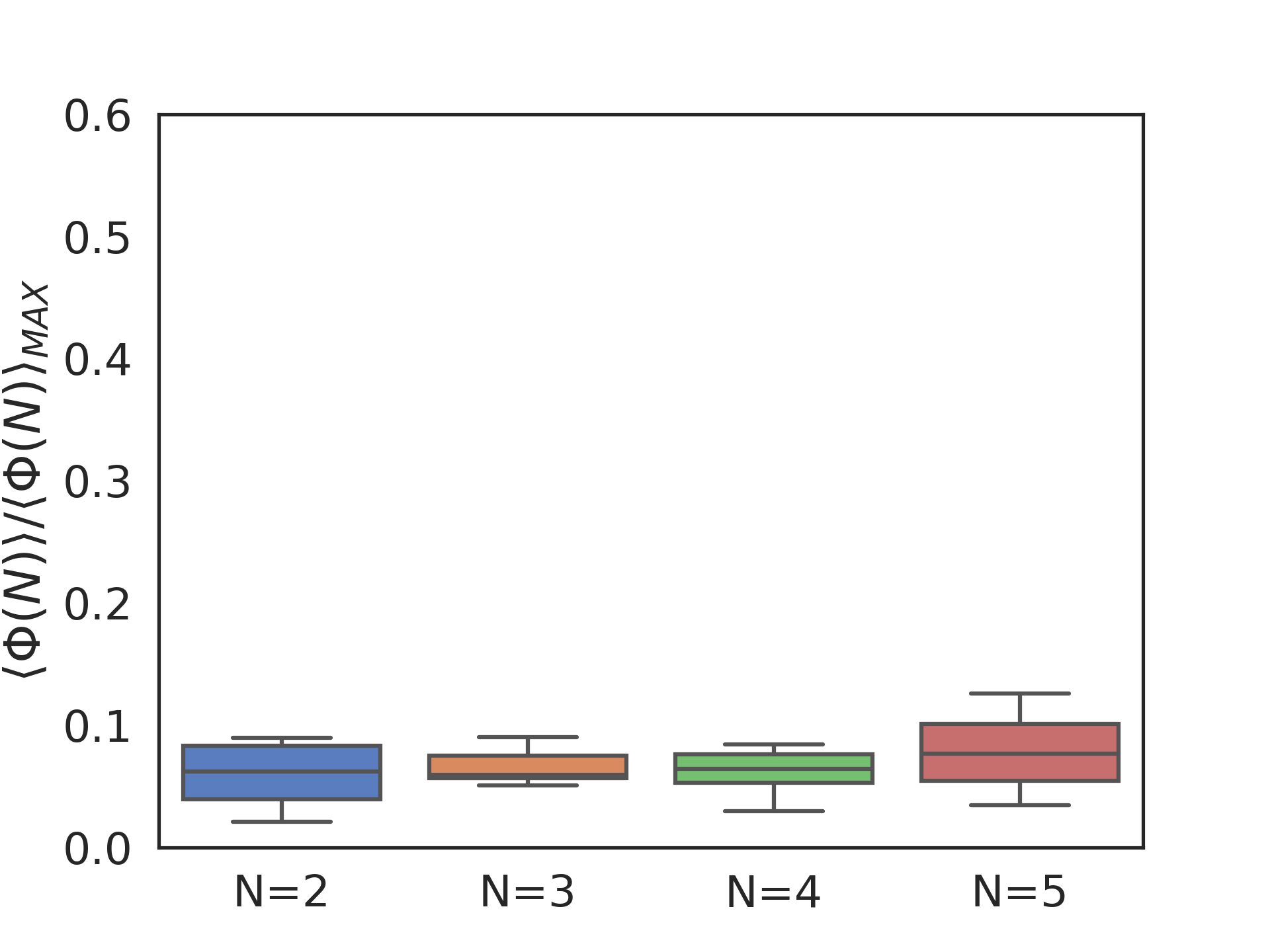

Supplement: Supplementary file 1 [file entropy-22-00726-s001.zip › Supporting Information/Figure_S4.png]

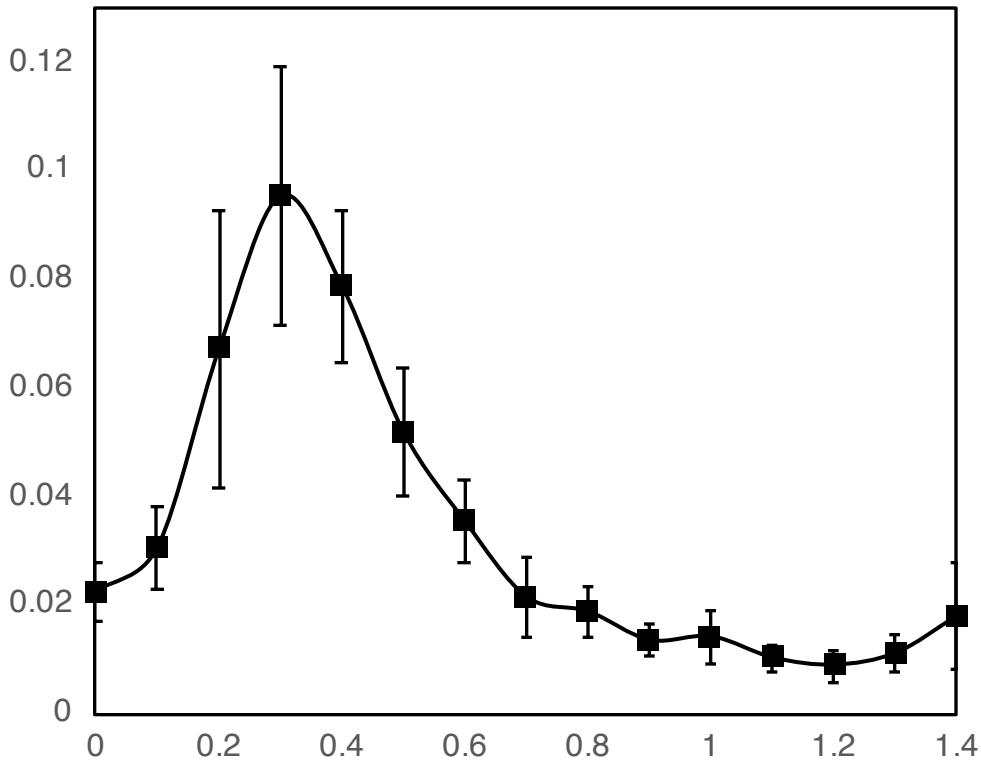

Supplement: Supplementary file 1 [file entropy-22-00726-s001.zip › Supporting Information/Figure_S7.pdf]
